# Evaluation of neoadjuvant immunotherapy and traditional neoadjuvant therapy for resectable esophageal cancer: a systematic review and single-arm and network meta-analysis

**DOI:** 10.3389/fimmu.2023.1170569

**Published:** 2023-05-12

**Authors:** Hesong Wang, Chunyang Song, Xiaohan Zhao, Wenzhao Deng, Jing Dong, Wenbin Shen

**Affiliations:** Department of Radiation Oncology, Fourth Hospital of Hebei Medical University, Shijiazhuang, Hebei, China

**Keywords:** neoadjuvant therapy, immunotherapy, neoadjuvant immunotherapy, curative resection, esophageal carcinoma, meta-analysis

## Abstract

**Objective:**

This systematic review and meta-analysis aimed to investigate the role of neoadjuvant immunochemotherapy with or without radiotherapy [NIC(R)T] compared to traditional neoadjuvant therapies, without immunotherapy [NC(R)T].

**Summary background data:**

NCRT followed by surgical resection is recommended for patients with early-stage esophageal cancer. However, it is uncertain whether adding immunotherapy to preoperative neoadjuvant therapy would improve patient outcomes when radical surgery is performed following neoadjuvant therapy.

**Methods:**

We searched PubMed, Web of Science, Embase, and Cochrane Central databases, as well as international conference abstracts. Outcomes included R0, pathological complete response (pCR), major pathological response (mPR), overall survival (OS) and disease-free survival (DFS) rates.

**Results:**

We included data from 5,034 patients from 86 studies published between 2019 and 2022. We found no significant differences between NICRT and NCRT in pCR or mPR rates. Both were better than NICT, with NCT showing the lowest response rate. Neoadjuvant immunotherapy has a significant advantage over traditional neoadjuvant therapy in terms of 1-year OS and DFS, with NICT having better outcomes than any of the other three treatments. There were no significant differences among the four neoadjuvant treatments in terms of R0 rates.

**Conclusions:**

Among the four neoadjuvant treatment modalities, NICRT and NCRT had the highest pCR and mPR rates. There were no significant differences in the R0 rates among the four treatments. Adding immunotherapy to neoadjuvant therapy improved 1-year OS and DFS, with NICT having the highest rates compared to the other three modalities.

**Systematic Review Registration:**

https://inplasy.com/inplasy-2022-12-0060/, identifier INPLASY2022120060.

## 1 Introduction

Esophageal cancer is the seventh most common malignant tumor and the sixth leading cause of cancer-related mortality worldwide (1). Surgical resection has advocated for the treatment of early-stage esophageal cancer (2). The CROSS trial showed that neoadjuvant chemoradiation followed by surgical resection was more beneficial for esophageal cancer (3). Accordingly, the National Comprehensive Cancer Network guidelines recommend it as the standard therapy (4). Nevertheless, the treatment efficacy for esophageal cancer remains poor, with a 5-year survival rate of approximately 20% (5, 6).

Immunotherapy has become an effective treatment for many malignancies including esophageal cancer (7–9). By rescuing the immune checkpoint pathway to resist carcinoma, the anti-tumor action of T cells is blocked by immune checkpoint blockade. Immunotherapy has proven beneficial as a third-, second-, and even first-line treatment for patients with esophageal cancer. However, it remains unclear whether adding immunotherapy therapy to preoperative neoadjuvant confers an overall benefit to patient outcomes when radical surgery is performed after neoadjuvant therapy. Several studies have documented benefits when immunotherapy is added to neoadjuvant therapy (10, 11); on the other hand, adding immunotherapy to neoadjuvant therapy increases the severity of toxic side effects (12, 13).

Therefore, this systematic review and meta-analysis was conducted to evaluate the outcomes of patients treated with either of two neoadjuvant immunotherapies – neoadjuvant immunotherapy combined with chemoradiotherapy (NICRT) and neoadjuvant immunochemotherapy (NICT) – compared with two traditional neoadjuvant therapies – neoadjuvant chemoradiotherapy (NCRT) and neoadjuvant chemotherapy (NCT).

## 2 Methods

This study was conducted using the Preferred Reporting Items for Systematic Reviews and Meta-Analyses (PRISMA) 2020 (14). The present study was registered in the INPLASY (identifier: INPLASY2022120060).

### 2.1 Search strategy and eligibility criteria

We searched PubMed, Web of Science, Embase, and Cochrane Central databases, as well as international conference abstracts from American Society of Clinical Oncology, European Society for Medical Oncology and American Association for Cancer Research, along with various other resources, until December 16, 2022. The detailed search strategies are summarized in **Supplementary Table 1**. We searched for studies that explored patients with histologically confirmed-, resectable-, esophageal carcinoma who received either NICRT or NICT followed by surgery. Meanwhile, the patients treated with traditional neoadjuvant therapy (NCRT or NCT) were all derived from control patients in these studies, rather than from other studies that did not involve NICRT or NICT. We followed the Population, Intervention, Comparison, Outcome, and Study Design (PICOS) principles (**Supplementary Table 2**). The detailed inclusion and exclusion criteria are shown in **Supplementary Table 3**.

### 2.2 Study selection and data extraction

Two authors (CS and XZ) independently assessed each study and extracted the pertinent information therefrom. Another author (WD) resolved any differences that might have arisen in the process. Relevant parameters were extracted from each included study: author, year, country, study type, registration number, intervention model, type of article, treatment modalities and side effects, sample size, age, sex, histologic subtype, relevant clinical characteristics, and outcome data of interest.

### 2.3 Outcomes

The outcome indicators in this study included direct measures of treatment efficacy – R0, pathological complete response (pCR), and major pathological response (mPR) rates – as well as survival-related indicators, including overall survival (OS), disease-free survival (DFS), and death within 30 days after surgery. We did not include treatment-related adverse events during neoadjuvant therapy or post-operative complications, as the evaluation criteria used to evaluate these indicators were not uniform across different studies. The primary goal of our study was to explore immediate post-treatment efficacy and subsequent survival outcomes, to investigate the effectiveness of neoadjuvant immunotherapy.

### 2.4 Quality assessment

Two authors (CS and XZ) independently evaluated the quality of each study. If there were any disagreements in the process, another author (JD) settled it. The Methodological Index for Non-randomized Studies (MINORS) was used to assess single-arm and retrospective dual-arm studies (15, 16). Each item was scored from 0 to 2. There were 8 items for non-comparative studies and 12 items for comparative studies. For non-comparative studies, an overall score > 12 was considered high, between 8 and 12 was considered intermediate, and < 8 was considered low. The Cochrane Risk of Bias tool was used to assess randomized controlled trials (RCT) (17, 18). The tool scores RCT studies according to five items. The overall bias included low risk of bias, some concerns, and high risk of bias. The quality of this systematic review and meta-analysis was evaluated according to the PRISMA 2020 Checklist (14) and the AMSTAR-2 Checklist (19).

### 2.5 Statistical analysis

All analyses were performed by STATA (STATA, version 14.0, College, TX),. Survival curve data from included studies which were not reported were extracted by Engauge Digitizer, version 12.1 (http://markummitchell.github.io/engauge-digitizer/). We performed a single-group meta-analysis of all included studies. In order to compare the four different neoadjuvant treatment modalities with each other and to rank their respective efficacies, we performed a network meta-analysis of the comparative studies among them. The significance level of the results was set at P <0.05, as per the convention. The combined risk ratio (RR) and 95% confidence interval (CI) were used as the outcome indicators. For OS and DFS rates, the number of events used to calculate the RR value was the number of survivors rather than the number of deaths. Therefore, in the present study, RR and 95% CI > 1 indicated that treatment was more conducive to survival, whereas RR and 95% CI < 1 indicated that treatment was more detrimental to survival (Detailed data synthesis are shown in **Supplementary Table 4**).

Subsequently, we merged NICRT and NICT into the neoadjuvant immunotherapy group and NCRT and NCT into the traditional neoadjuvant therapy group. We performed a traditional pairwise meta-analysis of these two groups, with head-to-head studies to explore the comparative advantages of neoadjuvant immunotherapy *vs.* traditional neoadjuvant therapy. Exploratory subgroup analyses were performed based on the study type (prospective or retrospective), intervention model (single-arm or dual-arm), immunotherapy drugs (PD-1 or PD-L1 inhibitors), and cancer type (squamous cell carcinoma [SCC] or adenocarcinoma [AC]). Additionally, sensitivity analyses were performed by omitting each study to evaluate the stability of the results. Publication bias was assessed using the Begg’s funnel plot (20).

## 3 Results

### 3.1 Characteristics

**Figure 1:**
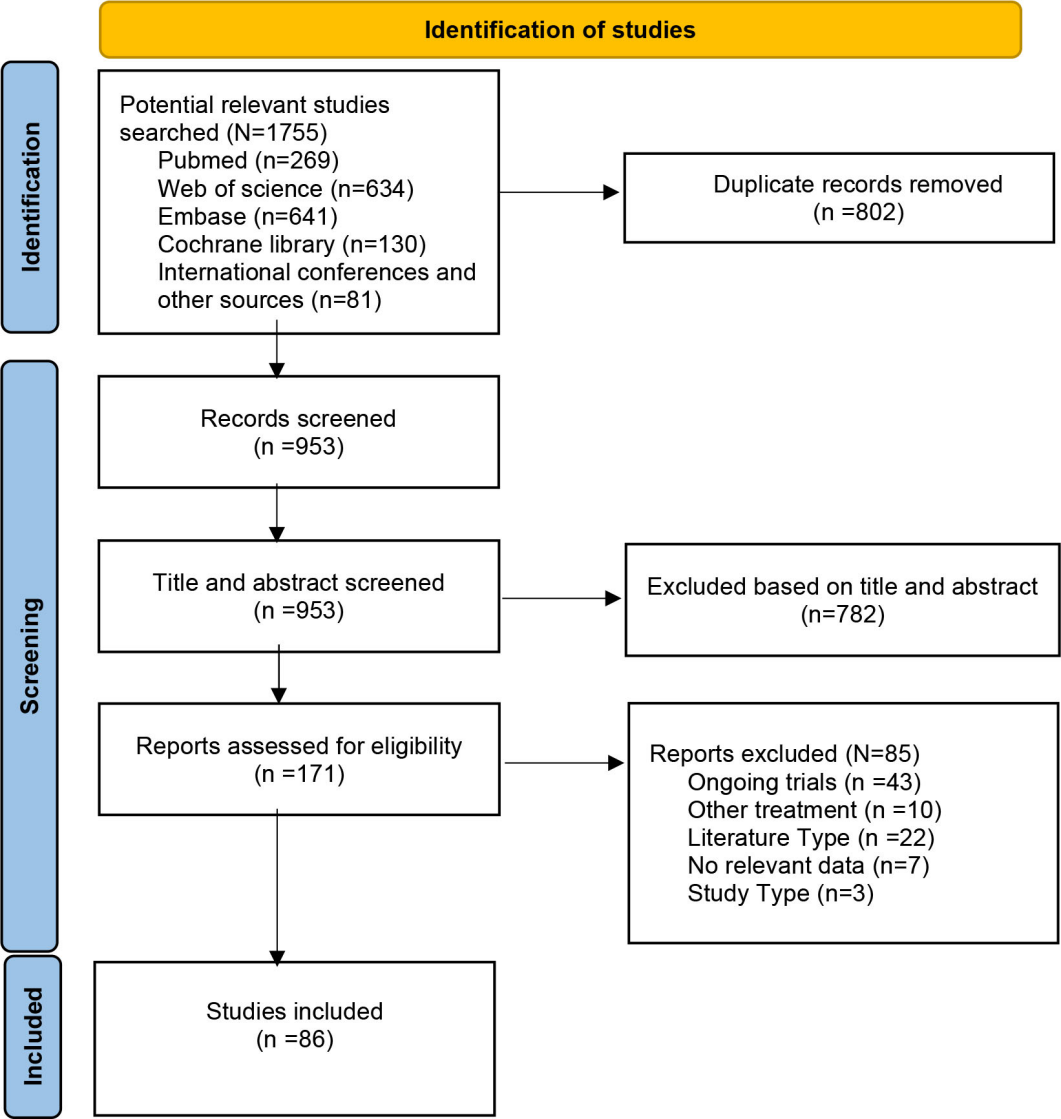
PRISMA flow diagram.

From the 1,755 considered studies, we eventually selected 86 studies (10–13, 21–102) describing a total of 5,034 patients (**Figure 1**). This number consisted of 16 dual-arm studies and 70 single-arm studies, five RCTs and 81 non-RCTs.

All studies were published between 2019 and 2022, most of which were conducted in China. Among these studies, the number of patients who received NICRT, NICT, NCRT, and NCT were 427, 3508, 701, and 398, respectively. The median age of all patients ranged from 42.7 to 68.8. For cancer type, the studies included SCC only (n=73), AC only (n=6), mixed SCC and AC (n=5), and undetailed pathology (n=2). For neoadjuvant immunotherapy, PD-1 inhibitors were the most common, with only 6 studies using PD-L1 inhibitors. The radiation doses ranged from 30 Gy to 56 Gy. All neoadjuvant chemotherapy regimens were conventional treatment regimens. Detailed characteristics of each study are shown in **Table 1** and **Supplementary Tablea 5**-**8**.

**Table 1:** Study Characteristics.

Author	Year	Country	Study Type	Registration Number	Study Title	Intervention Model	Type of Article	Treatment	Sample Size, No	Age, y	Gender, No. (%)	Histologic Subtype, No. (%)	ICI Drugs	CT Regimen	RT Dose, Gy
M.Zhu	2022	USA	prospective	NCT02730546	MC1541	dual-arm	full text	NICRT	31	62.0 (44.0-76.0)	male:30 (96.8%) female:1 (3.2%)	AC:31 (100.0%)	Pembrolizumab	TC	41.4
								NCRT	93	–	–	–	–	TC	41.1-50.4
Y.Zhou	2022	China	retrospective	–	–	dual-arm	full text	NICT	14	>60:6 (42.9%) ≤60:8 (57.1%)	male:9 (64.3%) female:5 (35.7%)	SCC:14 (100.0%)	Toripalimab	TC	–
								NCRT	14	>60:9 (64.3%) ≤60:5 (35.7%)	male:14 (100%) female:0 (0%)	SCC:14 (100.0%)	–	TP	40.0
Zh.Zhang	2022	China	prospective	ChiCTR1900026593	–	single-arm	full text	NICT	47	66.0 (64.0-70.0)	male:36 (76.6%) female:11 (23.4%)	SCC:47 (100.0%)	Sintilimab	TC	–
Y.Zhang	2022	China	prospective	ChiCTR2000039170	–	single-arm	Conference abstract	NICT	166	62.9 (53.7-72.1)	male:140 (84.2%) female:26 (15.8%)	–	Camrelizumab	TP	–
X.K.Zhang	2022	China	retrospective	–	–	single-arm	full text	NICT	64	62.0	male:50 (78.1%) female:14 (21.9%)	SCC:64 (100.0%)	Camrelizumab	TP/FP	–
G.Q.Zhang	2022	China	prospective	–	–	single-arm	Conference abstract	NICT	54	–	–	SCC:54 (100.0%)	Toripalimab	T+S1	–
Y.Yu	2022	China	retrospective	–	–	single-arm	full text	NICT	79	62.0 (48.0-78.0)	male:58 (73.4%) female:21 (26.6%)	SCC:79 (100.0%)	Tislelizumab Camrelizumab Toripalimab Keytruda Sintilimab	TP	–
G.Yin	2022	China	retrospective	–	–	single-arm	full text	NICT	34	59.0 (52.0-69.0)	male:30 (88.2%) female:4 (11.8%)	SCC:34 (100.0%)	Camrelizumab	TC	–
Y.Yang	2022	China	retrospective	–	–	dual-arm	full text	NICRT	30	62.0 (42.0-68.0)	male:28 (93.3%) female:2 (6.7%)	SCC+AC	Pembrolizumab Camrelizumab Toripalimab Tislelizumab	TP/TC/FP	41.4
								NICT	299	64.0 (43.0-81.0)	male:249 (83.3%) female:50 (16.7%)	SCC+AC	Pembrolizumab Camrelizumab Sintilimab Toripalimab Tislelizumab	TP/TC/FP	–
W.Yang	2022	China	prospective	ChiCTR2000028900	–	single-arm	full text	NICT	23	58.6 (48.6-68.7)	male:22 (95.7%) female:1 (4.3%)	SCC:23 (100.0%)	Camrelizumab	TC	–
G.Yang	2022	China	retrospective	–	–	single-arm	Conference abstract	NICT	47	–	–	SCC:47 (100.0%)	Camrelizumab	T+capecitabine	–
X.Yan	2022	China	prospective	ChiCTR2000037488	TD-NICE	single-arm	full text	NICT	45	68.8 (56.9-70.7)	male:27 (60.0%) female:18 (40.0%)	SCC:45 (100.0%)	Tislelizumab	TC	–
X.Xu	2022	China	prospective	NCT04437212	–	single-arm	Conference abstract	NICRT	20	–	–	SCC:20 (100.0%)	Toripalimab	TP	41.4
W.Xu	2022	China	prospective	–	–	single-arm	Conference abstract	NICT	46	–	–	SCC:46 (100.0%)	Camrelizumab	TC	–
L.Xu	2022	China	retrospective	–	–	dual-arm	full text	NICT	314	>60:184 (58.6%) ≤60:130 (41.4%)	male:263 (83.8%) female:51 (16.2%)	SCC:314 (100.0%)	Camrelizumab Sintilimab Tislelizumab Pembrolizumab	TP/TC/DP/FP	–
								NCRT	154	>60:78 (50.6%) ≤60:76 (49.4%)	male:132 (85.7%) female:22 (14.3%)	SCC:154 (100.0%)	–	TP/TC/DP/FP	32.4-50.4
L.W.Xu	2022	China	prospective	NCT04506138	–	single-arm	full text	NICT	46	63.3 (57.6-70.0)	male:44 (95.7%) female:2 (4.3%)	SCC:46 (100.0%)	Camrelizumab	–	–
X.Xiao	2022	China	retrospective	–	–	dual-arm	full text	NICT	55	66.0 (61.0-71.0)	male:46 (83.6%) female:9 (16.4%)	SCC:55 (100.0%)	Pembrolizumab Sintilimab Tislelizumab Camrelizumab	TP	–
								NCRT	94	64.0 (57.0-69.0)	male:77 (81.9%) female:17 (18.1%)	SCC:94 (100.0%)	–	TP	40-50.4
P.Xia	2022	China	retrospective	–	–	single-arm	full text	NICT	66	67.5 (59.0-71.0)	male:60 (90.9%) female:6 (9.1%)	SCC:66 (100.0%)	Camrelizumab	TP/TC	–
X.Wang	2022	China	prospective	–	–	single-arm	full text	NICT	59	59.0 (43.0-79.0)	male:46 (79.3%) female:12 (20.7%)	SCC:59 (100.0%)	Camrelizumab	TC	–
W.Wang	2022	China	prospective	–	–	single-arm	Conference abstract	NICT	22	–	–	SCC:22 (100.0%)	Pembrolizumab	DP	–
R.Wang	2022	China	prospective	ChiCTR2000033252	–	single-arm	Conference abstract	NICT	30	–	–	SCC:30 (100.0%)	Camrelizumab	DP	–
N.V.Uboha	2022	USA	prospective	NCT03490292	–	single-arm	Conference abstract	NICRT	22	64.0	male:20 (90.9%) female:2 (9.1%)	SCC:3 (13.6%) ACC:19 (86.4%)	Avelumab	TC	41.4
Y.Qiao	2022	China	retrospective	–	–	dual-arm	full text	NICT	48	64.2 (56.9-71.4)	male:38 (79.2%) female:10 (20.8%)	SCC:48 (100.0%)	Camrelizumab	–	–
								NCT	206	62.2 (55.1-69.3)	male:147 (71.4%) female:59 (28.6%)	SCC:206 (100.0%)	–	–	–
Y.Qi	2022	China	prospective	NCT03917966	–	single-arm	Conference abstract	NICT	62	66.0	–	SCC:62 (100.0%)	Camrelizumab	DP	–
S. Matsuda	2022	Japan	prospective	NCT03914443	JCOG1804E	single-arm	Conference abstract	NICT	12	–	–	SCC:12 (100.0%)	Nivolumab	DCF	–
X.Ma	2022	China	retrospective	–	–	single-arm	full text	NICT	34	61.0 (47.0-74.0)	male:31 (91.2%) female:3 (8.8%)	SCC:34 (100.0%)	Pembrolizumab Camrelizumab Sintilimab Toripalimab Tislelizumab	TP	–
H.Lv	2022	China	retrospective	–	–	single-arm	full text	NICT	96	65.0 (60.0-69.0)	male:67 (69.8%) female:29 (30.2%)	SCC:96 (100.0%)	Sintilimab	TP/DP	–
Jun.Liu	2022	China	prospective	ChiCTR1900026240	NICE study	single-arm	full text	NICT	60	65.0 (48.0-74.0)	male:50 (83.3%) female:10 (16.7%)	SCC:60 (100.0%)	Camrelizumab	TC	–
J.Liu	2022	China	prospective	NCT04225364	NIC-ESCC2019	single-arm	full text	NICT	56	61.0 (40.0-70.0)	male:42 (75.0%) female:14 (25.0%)	SCC:56 (100.0%)	Camrelizumab	TP	–
Z.Li	2022	China	prospective	–	–	single-arm	Conference abstract	NICT	20	67.5 (47.0-75.0)	male:16 (80.0%) female:4 (20.0%)	SCC:20 (100.0%)	Sintilimab	TP	–
Y.Li	2022	China	prospective	NCT04460066	–	dual-arm	Conference abstract	NICT	32	–	–	SCC:32 (100.0%)	Socazolimab	TP	–
								NCT	32	–	–	SCC:32 (100.0%)	–	TP	–
A.H.Ko	2022	USA	prospective	NCT03165994	–	single-arm	Conference abstract	NICRT	34	–	–	SCC:8 (23.5%) AC:26 (76.5%)	Sotigalimab	TC	50.4
R.J.Kelly	2022	USA	prospective	NCT03044613	–	single-arm	Conference abstract	NICRT	32	65.0 (39.0-73.0)	male:26 (81.3%) female:6 (18.7%)	SCC:4 (12.5%) AC:28 (87.5%)	Nivolumab Relatlimab	TC	41.1
S.Jing	2022	China	retrospective	–	–	dual-arm	full text	NICT	47	>60:34 (72.3%) ≤60:13 (27.7%)	male:30 (63.8%) female:17 (36.2%)	SCC:47 (100.0%)	Pembrolizumab Camrelizumab Toripalimab Sintilimab	TP/FP	–
								NCT	47	>60:35 (74.5%) ≤60:12 (25.5%)	male:33 (70.2%) female:14 (29.8%)	SCC:47 (100.0%)	–	TP/FP	–
N.Jiang	2022	China	prospective	ChiCTR2100045104	SCALE-1	single-arm	Conference abstract	NICRT	23	–	–	SCC:23 (100.0%)	Toripalimab	TC	30.0
B.Jiang	2022	China	prospective	–	–	single-arm	Conference abstract	NICT	10	–	–	SCC:10 (100.0%)	Camrelizumab Sintilimab Tislelizumab	TP	–
S.J.Huang	2022	China	retrospective	–	–	single-arm	full text	NICT	51	60.0 (54.0-65.0)	male:41 (80.4%) female:10 (19.6%)	SCC:51 (100.0%)	Camrelizumab Nivolumab Pembrolizumab Sintilimab Tislelizumab	TP/DP	–
S.Huang	2022	China	retrospective	NCT04822103	RICE-Retro	single-arm	full text	NICT	155	61.0 (55.0-66.0)	male:121 (78.1%) female:34 (21.9%)	SCC:155 (100.0%)	Camrelizumab Pembrolizumab Sintilimab Tislelizumab Toripalimab Nivolumab	TP/DP	–
Z.Hong	2022	China	retrospective	–	–	dual-arm	full text	NICT	26	68.5 (51.1-65.9)	male:22 (84.6%) female:4 (15.4%)	SCC:26 (100.0%)	Camrelizumab Pembrolizumab Sintilimab	TP	–
								NCT	52	61.0 (54.6-67.4)	male:42 (80.8%) female:10 (19.2%)	SCC:48 (92.3%) non-SCC:4 (7.7%)	–	TP/FP	–
Z.N.Hong	2022	China	retrospective	–	–	dual-arm	full text	NICT	32	62.0 (55.0-67.0)	male:21 (65.5%) female:11 (34.5%)	SCC:32 (100.0%)	Camrelizumab Pembrolizumab Sintilimab	TP	–
								NCRT	32	60.0 (54.0-65.0)	male:27 (84.3%) female:5 (15.7%)	SCC:32 (100.0%)	–	TP/FP	40.0-56.0
W.He	2022	China	prospective	NCT04177797	–	single-arm	full text	NICT	20	62.1 (51.5-72.3)	male:15 (75.0%) female:5 (25.0%)	SCC:20 (100.0%)	Toripalimab	TC	–
J.Guo	2022	China	prospective	ChiCTR2000040345	–	single-arm	Conference abstract	NICT	15	–	–	SCC:15 (100.0%)	Sintilimab	TP	–
Y.M.Gu	2022	China	retrospective	–	–	single-arm	full text	NICT	38	66.0 (46.0-80.0)	male:27 (71.1%) female:11 (28.9%)	SCC:38 (100.0%)	Pembrolizumab Tislelizumab Camrelizumab Sintilimab Toripalimab	TP	–
T.Gong	2022	China	retrospective	–	–	single-arm	Conference abstract	NICT	37	62.0 (47.0-76.0)	male:30 (81.1%) female:7 (18.9%)	SCC:37 (100.0%)	Sintilimab	–	–
L.Gao	2022	China	prospective	ChiCTR2100052784	ESONICT-2	single-arm	full text	NICT	20	58.3 (49.0-69.0)	male:17 (85.0%) female:3 (15.0%)	SCC:20 (100.0%)	Toripalimab	DP	–
J.Feng	2022	China	retrospective	–	–	single-arm	full text	NICT	285	63.5 (56.9-70.1)	male:267 (93.7%) female:18 (6.3%)	SCC:285 (100.0%)	Nivolumab Pembrolizumab Camrelizumab Tislelizumab Sintilimab	TC	–
H.Duan	2022	China	prospective	ChiCTR2100048917	PEN-ICE	single-arm	full text	NICT	18	64.0 (35.0-78.0)	male:14 (77.8%) female:4 (22.2%)	SCC:18 (100.0%)	Pembrolizumab	TP/DP	–
Y.Dong	2022	China	prospective	ChiCTR2100050057	–	single-arm	Conference abstract	NICT	28	–	–	SCC:28 (100.0%)	Camrelizumab	TP	–
J.Cheng	2022	China	retrospective	–	–	dual-arm	full text	NICT	40	64.3 (55.4-73.2)	male:30 (75.0%) female:10 (25.0%)	SCC:40 (100.0%)	Pembrolizumab Tislelizumab Camrelizumab Sintilimab Toripalimab	TP/FP	–
								NCRT	109	62.7 (55.4-69.9)	male:93 (85.3%) female:16 (14.7%)	SCC:109 (100.0%)	–	TP/FP	40.0-50.0
F.Chen	2022	China	retrospective	–	–	single-arm	full text	NICRT	38	60.2 (54.4-66.0)	male:31 (81.6%) female:7 (18.4%)	SCC:38 (100.0%)	Camrelizumab	TC	–
L.Zhao	2021	China	prospective	–	–	single-arm	Conference abstract	NICT	30	–	–	SCC:30 (100.0%)	Toripalimab	TP	–
Z.Zhang	2021	China	prospective	–	–	single-arm	Conference abstract	NICT	40	–	–	SCC:40 (100.0%)	Sintilimab	TC	–
Z.Y.Zhang	2021	China	prospective	ChiCTR2100045659	ESONICT-1	single-arm	full text	NICT	30	58.3 (51.2-65.4)	male:26 (86.7%) female:4 (13.3%)	SCC:30 (100.0%)	Sintilimab	TP	–
X.Zhang	2021	China	prospective	ChiCTR2000029807	–	single-arm	Conference abstract	NICT	25	–	–	SCC:25 (100.0%)	Camrelizumab	T+S1	–
P.Yang	2021	China	prospective	ChiCTR2100051903	–	single-arm	full text	NICT	16	60.5 (56.0-67.3)	male:14 (87.5%) female:2 (12.5%)	SCC:16 (100.0%)	Camrelizumab	TC	–
G.Z.Yang	2021	China	retrospective	–	–	single-arm	full text	NICT	12	56.0 (50.0-65.0)	male:7 (58.3%) female:5 (41.7%)	SCC:12 (100.0%)	Camrelizumab	T+S1	–
S.Yamamoto	2021	Japan	prospective	NCT03914443	FRONTIER	single-arm	Conference abstract	NICT	13	62.0 (34.0-75.0)	–	–	Nivolumab	CF	–
W.Xing	2021	China	prospective	NCT03985670	–	single-arm	full text	NICT	30	63.8 (57.7-69.9)	male:22 (73.3%) female:8 (26.7%)	SCC:30 (100.0%)	Toripalimab	TP	–
Y.Xiao	2021	China	prospective	–	–	dual-arm	full text	NICT	30	42.7 (27.1-58.2)	male:15 (50.0%) female:15 (50.0%)	SCC:30 (100.0%)	Camrelizumab	Oxaliplatin + docetaxel	–
								NCT	30	43.6 (31.1-56.2)	male:14 (46.7%) female:16 (53.3%)	SCC:30 (100.0%)	–	Oxaliplatin + docetaxel	–
Z.Wu	2021	China	retrospective	–	–	single-arm	full text	NICT	38	61.0 (57.0-75.0)	male:36 (94.7%) female:2 (5.3%)	SCC:38 (100.0%)	Pembrolizumab Camrelizumab Sintilimab	TP/TC	–
P.Wu	2021	China	retrospective	–	–	single-arm	Conference abstract	NICT	20	65.0	male:17 (85.0%) female:3 (15.0%)	SCC:20 (100.0%)	Toripalimab Sintilimab Pembrolizumab Camrelizumab Tislelizumab	TP	–
F.Wang	2021	China	prospective	–	–	single-arm	Conference abstract	NICT	26	63.0	male:17 (65.3%) female:9 (34.7%)	SCC:26 (100.0%)	Camrelizumab	DP	–
T.V.D.Ende	2021	Netherlands	prospective	NCT03087864	PERFECT	single-arm	full text	NICRT	40	63.0 (40.0-75.0)	male:35 (87.5%) female:5 (12.5%)	AC:40 (100.0%)	Atezolizumab	TC	41.4
S.Sihag	2021	USA	retrospective	NCT02962063	–	dual-arm	full text	NICRT	25	61.5 (53.0-67.0)	male:22 (88.0%) female:3 (12.0%)	AC:25 (100.0%)	Durvalumab	FOLFOX	–
								NCRT	143	64.0 (56.0-70.0)	male:123 (86.0%) female:20 (14.0%)	AC:143 (100.0%)	–	–	–
D.Shen	2021	China	prospective	–	–	single-arm	full text	NICT	28	62.2 (48.0-79.0)	male:27 (96.4%) female:1 (3.6%)	SCC:28 (100.0%)	Nivolumab Pembrolizumab Camrelizumab	TC	–
X.Shang	2021	China	prospective	NCT04389177	Keystone-001	single-arm	Conference abstract	NICT	42	–	–	SCC:42 (100.0%)	Pembrolizumab	TP	–
M.A.Shah	2021	USA	prospective	NCT02998268	–	dual-arm	Conference abstract	NICRT	40	68.0 (38.0-81.0)	male:32 (80.0%) female:8 (20.0%)	AC:40 (100.0%)	Pembrolizumab	TC	41.4
								NCRT	40	–	–	AC:40 (100.0%)	–	TC	41.4
J.Ma	2021	China	prospective	ChiCTR2000033761	ESPRIT	single-arm	Conference abstract	NICT	48	62.0	–	SCC:48 (100.0%)	Camrelizumab	TP	–
Lv	2021	China	retrospective	–	–	single-arm	Conference abstract	NICT	28	–	–	SCC:28 (100.0%)	Camrelizumab	TP	–
Hui.Lv	2021	China	retrospective	–	–	single-arm	Conference abstract	NICT	101	65.0 (43.0-78.0)	male:71 (70.3%) female:30 (29.7%)	SCC:101 (100.0%)	–	–	–
H.L.Lv	2021	China	retrospective	–	–	single-arm	Conference abstract	NICT	16	–	–	SCC:16 (100.0%)	Camrelizumab	TP	–
D.Liu	2021	China	prospective	ChiCTR1900025318	–	single-arm	Conference abstract	NICT	23	–	–	SCC:23 (100.0%)	Toripalimab	TP	–
C.Li	2021	China	prospective	NCT03792347	PALACE-1	single-arm	full text	NICRT	20	62.0 (42.0-66.0)	male:19 (95.0%) female:1 (5.0%)	SCC:20 (100.0%)	Pembrolizumab	TC	41.4
G.Y.Ku	2021	USA	prospective	–	–	single-arm	Conference abstract	NICRT	36	–	–	AC:36 (100.0%)	Durvalumab	mFOLFOX6	50.4
B.Huang	2021	China	prospective	–	–	dual-arm	full text	NICT	23	59.2 (51.9-66.5)	male:21 (91.3%) female:2 (8.7%)	SCC:23 (100.0%)	Pembrolizumab	DP	–
								NCT	31	58.9 (52.5-65.3)	male:30 (96.7%) female:1 (3.3%)	SCC:31 (100.0%)	–	DP	–
Hong	2021	China	retrospective	–	–	single-arm	full text	NICT	38	58.8 (51.2-66.4)	male:22 (57.9%) female:16 (42.1%)	SCC:38 (100.0%)	Sintilimab Pembrolizumab Camrelizumab	TP	–
H.T.Duan	2021	China	prospective	–	SIN-ICE	single-arm	full text	NICT	23	63.5 (56.0-81.0)	male:21 (91.3%) female:2 (8.7%)	SCC:23 (100.0%)	Sintilimab	DP/TP	–
C.Cheng	2021	China	prospective	–	–	single-arm	Conference abstract	NICT	20	–	–	SCC:20 (100.0%)	Camrelizumab	TC	–
A.Athauda	2021	UK	prospective	NCT03399071	ICONIC	single-arm	Conference abstract	NICT	15	63.0 (25.0-73.0)	–	AC:15 (100.0%)	Avelumab	FLOT	–
G.Zhang	2020	China	prospective	ChiCTR1900027160	–	single-arm	Conference abstract	NICT	24	–	–	SCC:24 (100.0%)	Toripalimab	T+S1	–
W.Qi	2020	China	prospective	–	–	single-arm	Conference abstract	NICRT	40	61.2 (39.0-66.0)	male:19 (95.0%) female:1 (5.0%)	SCC:40 (100.0%)	Pembrolizumab	TC	41.4
S.Y.Park	2020	Korea	retrospective	NCT02844075	–	dual-arm	full text	NICRT	16	58.5 (56.5-66.0)	male:13 (81.3%) female:3 (18.7%)	SCC:16 (100.0%)	Pembrolizumab	TC	44.1
								NCRT	22	61.5 (56.3-66.0)	male:18 (81.8%) female:4 (18.2%)	SCC:22 (100.0%)	–	FP	44.1
K.Li	2020	China	prospective	–	–	single-arm	Conference abstract	NICT	17	–	–	SCC:17 (100.0%)	Toripalimab	TC	–
H.Li	2020	China	prospective	NCT03604991	–	single-arm	Conference abstract	NICRT	20	–	–	SCC:20 (100.0%)	Pembrolizumab	TC	41.4
Y.Gu	2020	China	prospective	NCT03946969	KEEP-G 03	single-arm	Conference abstract	NICT	17	65.0 (42.0-69.0)	male:13 (76.4%) female:4 (23.6%)	SCC:17 (100.0%)	Sintilimab	TP+S1	–
S.Lee	2019	Korea	prospective	–	–	single-arm	Conference abstract	NICRT	28	60.0	–	SCC:28 (100.0%)	Pembrolizumab	TC	44.1

ICI, immune checkpoint inhibitor; CT, chemotherapy; RT, radiotherapy; NICRT, neoadjuvant immunotherapy combined with chemoradiotherapy; NICT, neoadjuvant immunochemotherapy; NCRT, neoadjuvant chemoradiotherapy; NCT, neoadjuvant chemotherapy; AC, adenocarcinoma; SCC, squamous cell carcinoma.

### 3.2 Clinical outcomes of NICRT, NICT, NCRT and NCT

**Figure 2:**
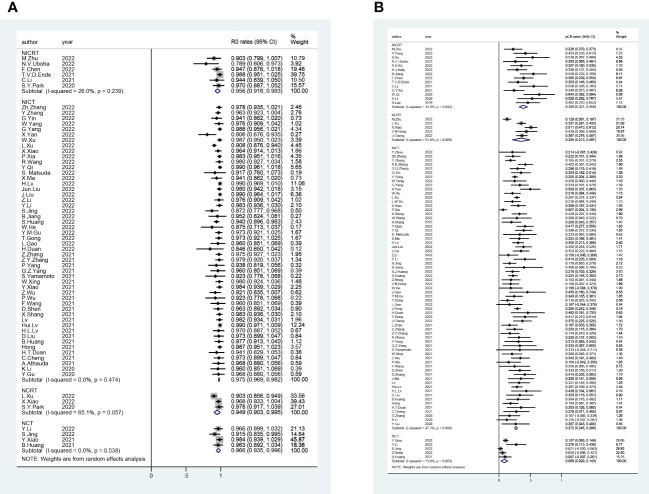
Forest Plot of **(A)** R0 and **(B)** Pathological Complete Response (pCR).

**Figure 3:**
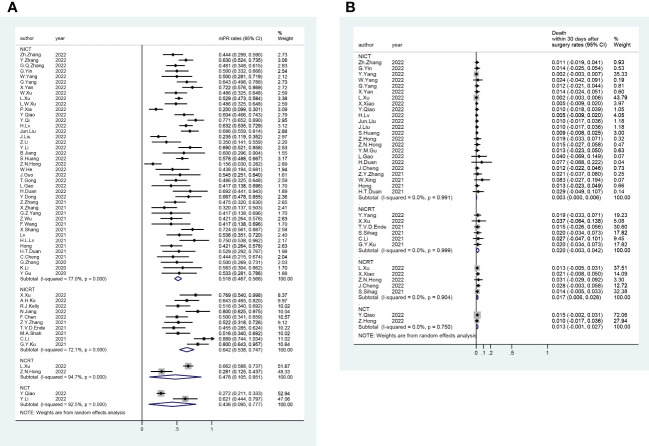
Forest Plot of **(A)** Major Pathological Response (mPR) and **(B)** Death within 30 Days after Surgery.

**Figure 4:**
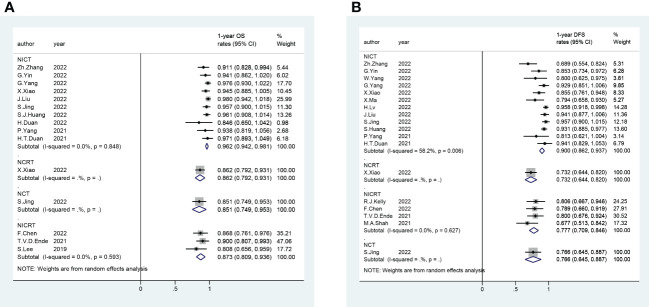
Forest Plot of **(A)** 1-year Overall Survival (OS) and **(B)** 1-year Disease Free Survival (DFS).

A total of 56 trials reported R0 rates (pooled R0 rate and 95% CI: NICRT - 95.6% [91.8%-99.3%]; NICT - 97.5% [96.9%-98.2%]; NCRT - 94.9% [90.3%-99.5%]; NCT - 96.6% [93.5%-99.6%]) (**Figure 2**). Overall, 80 trials provided pCR rates (pooled pCR rate and 95% CI: NICRT - 38.9% [32.1%-45.6%]; NICT -27.2% [24.8%-29.6%]; NCRT - 35.5% [21.3%-49.7%]; NCT - 8.6% [2.9%-14.3%]) (**Figure 2**). Totally, 51 trials analyzed mPR rates (pooled mPR rate and 95% CI: NICRT - 64.2% [53.8%-74.7%]; NICT - 51.8% [46.7%-56.8%]; NCRT - 47.8% [10.5%-85.1%]; NCT - 43.6% [9.5%-77.7%]) (**Figure 3**). In terms of survival outcomes, 28 trials reported death within 30 days after surgery (pooled rate and 95% CI: NICRT - 2.0% [0.0%-4.2%]; NICT - 0.3% [0.0%-0.6%]; NCRT - 1.7% [0.6%-2.8%]; NCT - 1.3% [0.0%-2.7%]) (**Figure 3**). Thirteen trials provided 1-year OS rates (pooled 1-year OS rate and 95% CI: NICRT - 87.3% [80.9%-93.6%]; NICT - 96.2% [94.2%-98.1%]; NCRT - 86.2% [79.2%-93.1%]; NCT - 85.1% [74.9%-95.3%]) (**Figure 4**). And a total of 16 trials analyzed 1-year DFS rates (pooled 1-year DFS rate and 95% CI: NICRT - 77.7% [70.9%-84.6%]; NICT - 90.0% [86.2%-93.7%]; NCRT - 73.2% [64.4%-82.0%]; NCT - 76.6% [64.5%-88.7%]) (**Figure 4**).

**Figure 5:**
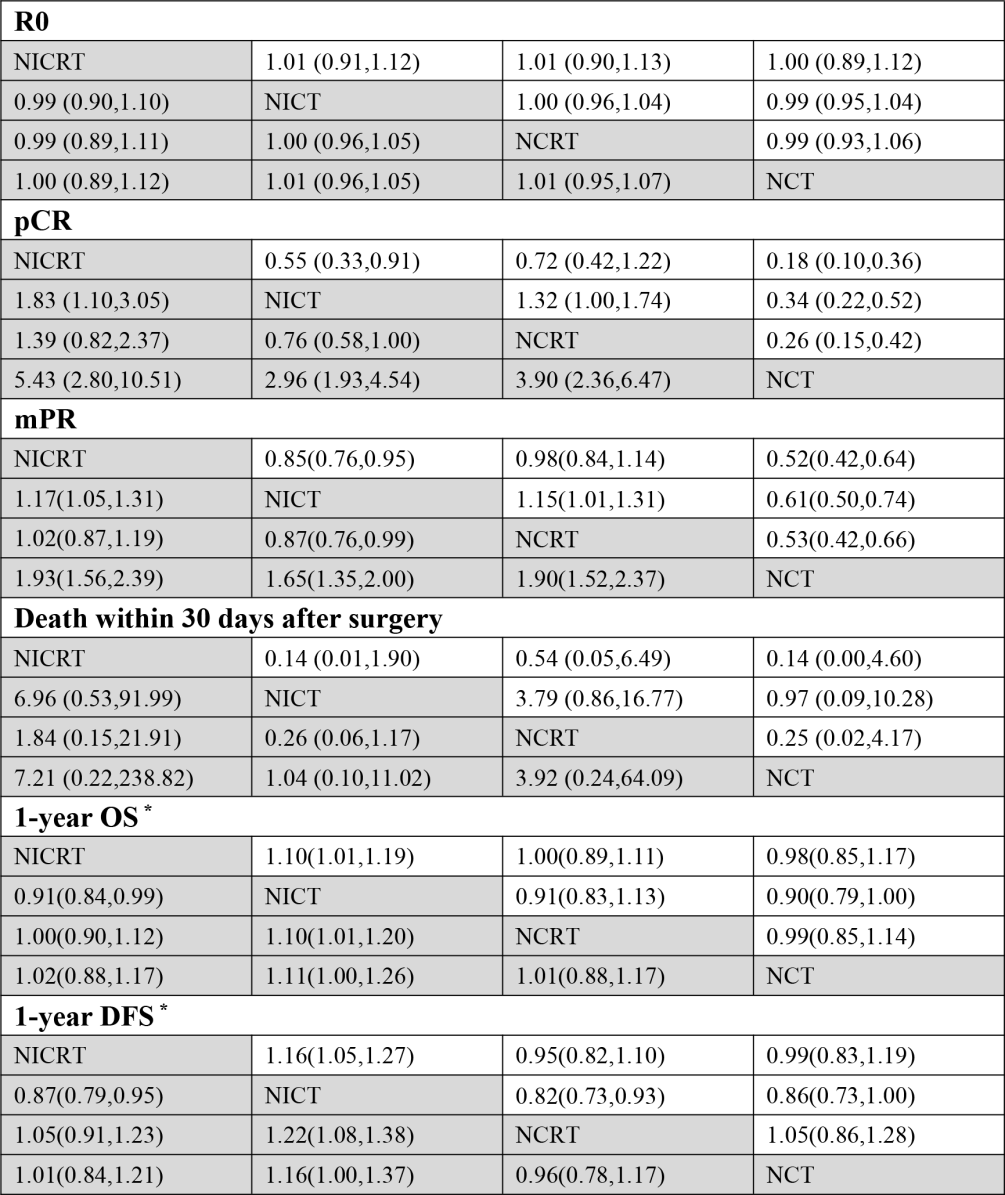
Results of Comparisons by Risk Ratio (RR) and 95% Confidence Interval (CI) among Four Neoadjuvant Therapies. (*The number of events in the calculation of the RR value is the number of survivors rather than the number of deaths. RR and 95% CI > 1 indicates that treatment is more conducive to survival, while RR and 95% CI < 1 indicates that treatment is more detrimental to survival.) Pathological Complete Response (pCR), Major Pathological Response (mPR), Overall Survival (OS), Disease Free Survival (DFS).

To compare different neoadjuvant treatment modalities with each other, we included 16 dual-arm trials in the network meta-analysis. Network evidence plots and contribution plots are shown in **Supplementary Figures 1**, **2**. The network estimates are shown in **Figure 5** and **Supplementary Figures 3**, **4**. There were no significant differences in pCR and mPR rates between NICRT and NCRT (pooled RR and 95% CI of pCR rate: NICRT *vs.* NCRT - 1.39 [0.82,2.37]; pooled RR and 95% of mPR rate: NICRT *vs.* NCRT - 1.02[0.87,1.19]). Both were superior to NICT (pooled RR and 95% CI of pCR rate: NICRT *vs.* NICT - 1.83 [1.10,3.05], NCRT *vs.* NICT - 1.32 [1.00,1.74]; pooled RR and 95% CI of mPR rate: NICRT *vs.* NICT - 1.17[1.05,1.31], NCRT *vs.* NICT - 1.15[1.01,1.31]), and NCT had the poorest results (pooled RR and 95% CI of pCR rate: NICRT *vs.* NCT - 5.43 [2.80,10.51], NCRT *vs.* NCT - 3.90 [2.36,6.47], NICT *vs.* NCT - 2.96 [1.93,4.54]; pooled RR and 95% CI of mPR rate: NICRT *vs.* NCT - 1.93 [1.56,2.39], NCRT *vs.* NCT - 1.90 [1.52,2.37], NICT *vs.* NCT - 1.65[1.35,2.00]). For 1-year OS and DFS rates, NICT showed the best rates compared to other three treatments (pooled RR and 95% CI of 1-year OS rate: NICT *vs.* NICRT - 1.10 [1.01,1.19], NICT *vs.* NCRT - 1.10 [1.01,1.20], NICT *vs.* NCT - 1.11[1.00,1.26]; pooled RR and 95% CI of 1-year DFS rate: NICT *vs.* NICRT - 1.16 [1.05,1.27], NICT *vs.* NCRT - 1.22 [1.08,1.38], NICT *vs.* NCT - 1.16 [1.00,1.37]), with the other three treatments not having any statistically significant difference in these parameters amongst each other. None of the treatment modalities stood out from the others in terms of R0 rates or death within 30 days after surgery.

### 3.3 Neoadjuvant immunotherapy (NICRT and NICT) versus traditional neoadjuvant therapy (NCRT and NCT)

**Figure 6:**
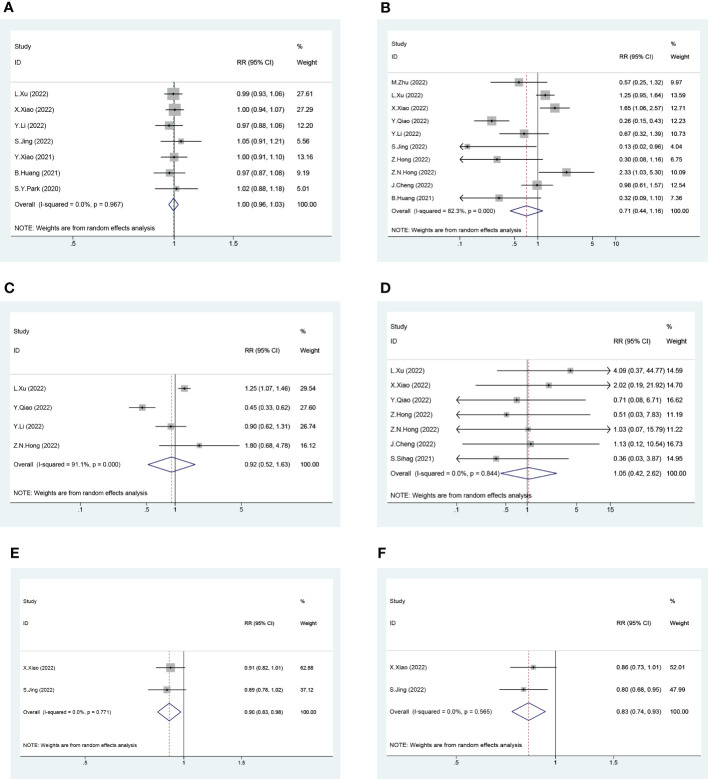
Forest Plot of Traditional Neoadjuvant Therapy (left) and Neoadjuvant Immunotherapy (right). **(A)** R0, **(B)** Pathological Complete Response (pCR), **(C)** Major Pathological Response (mPR), **(D)** Death within 30 Days after Surgery, **(E)** 1-year Overall Survival (OS) and **(F)** 1-year Disease Free Survival (DFS). (For 1-year OS and 1-year DFS, the number of events in the calculation of the RR value is the number of survivors rather than the number of deaths. RR and 95% CI > 1 indicates that treatment is more conducive to survival, while RR and 95% CI < 1 indicates that treatment is more detrimental to survival.).

Next, we pooled the data for the NICRT and NICT cases into the neoadjuvant immunotherapy group and the NCRT and NCT cases into the traditional neoadjuvant therapy group. A total of 16 trials were included in this head-to-head pairwise meta-analysis. Patients in the neoadjuvant immunotherapy group exhibited significantly higher 1-year OS and DFS rates than those in the traditional neoadjuvant therapy group (pooled RR and 95% CI of the traditional group *vs.* immunotherapy group: 1-year OS rate - 0.90 [0.83-0.98]; 1-year DFS rate - 0.83 [0.74-0.93]) (**Figure 6**). However, there were no significant differences between the two groups in terms of R0, pCR, mPR, or death within 30 days after surgery (**Figure 6**).

### 3.4 Exploratory subgroup analysis

To explore the potential association of immunotherapy between NICRT and NICT, we conducted exploratory subgroup analysis based on study type (prospective or retrospective), intervention model (single-arm or dual-arm), immunotherapy drugs (PD-1 or PD-L1 inhibitors), and cancer type (SCC or AC), respectively. The results of the subgroup NICRT and NICT analyses were generally consistent with the above results in terms of R0, pCR, mPR, death within 30 days after surgery, 1-year OS, and 1-year DFS (**Supplementary Figures 5**–**10**).

### 3.5 Quality evaluation, sensitivity analysis and publication bias

The details of the risk of bias are provided in **Supplementary Tables 9**, **10**. The MINORS was used to evaluate the 81 non-randomized studies. All the 81 studies were of high or intermediate quality. The Cochrane Risk of Bias tool was used to evaluate the five randomized studies, and it indicated that there was no high risk of bias in any of the evaluated categories among the five RCTs in our data set. **Supplementary Tables 11**, **12** show the quality evaluation of the present study using the PRISMA 2020 Checklist and AMSTAR-2 Checklist. Sensitivity analysis, conducted by omitting each study, indicated that all results were stable except for death within 30 days after surgery (**Supplementary Figure 11**). Similarly, there was no significant publication bias except for death within 30 days after surgery (**Supplementary Figure 12**).

## 4 Discussion

To the best of our knowledge, the present study is the first systematic review and meta-analysis to explore the effectiveness of four different neoadjuvant therapies (NICRT, NICT, NCRT, and NCT) followed by curative surgery for esophageal cancer, and then compare them with each other by not only postoperative outcome results but also survival-related efficacy outcomes. Drawing from data taken from 86 different studies, collectively describing 5,034 patients, we explored the comparison of R0, pCR, mPR, OS, DFS, and death within 30 days after surgery outcomes across treatment modalities. There were no significant differences in pCR and mPR rates between NICRT and NCRT; both were superior to NICT, and NCT had the poorest results. For 1-year OS and DFS rates, NICT showed the best rates compared to the other three treatments, with the other three treatments not having any statistically significant difference in these parameters amongst each other. No significant differences were observed among any of the four examined treatment modalities in terms of R0 rates or death within 30 days after surgery. As for the subgroup analyses based on the study type, intervention model, immunotherapy drugs, and cancer type, there were no significant differences between the subgroups, which is consistent with the above findings.

Although this is, to date, the largest meta-analysis to examine the role of four different neoadjuvant therapies after curative resection for esophageal cancer, previous studies on this subject have been conducted. A meta-analysis conducted by Wang et al. (103), which included 20 studies with 621 patients, explored the clinical outcomes of NICRT *vs.* NICT. Consistent with our findings, they reported that NICRT had an advantage over NICT in terms of mPR rates, but found no significant differences in R0 rates. However, they reported no significant differences in pCR rates between NICRT and NICT, whereas we found that NICRT had superior pCR rates to NICT (pooled RR and 95% CI: 1.83 [1.10, 3.05]). This discrepancy may be explained by Wang et al.’s smaller sample size, which included only two studies that involved NICRT. In contrast, our study included 14 studies of NICRT, including the two used by Wang et al. Additionally, the patients in the NCRT and NCT groups in their study were obtained from a meta-analysis by Li et al. (104), whereas the patients in our NCRT and NCT groups were extracted from dual-arm studies with direct head-to-head comparisons with NICRT or NICT. This significantly reduced error, increased comparability, and provided assurance of the quality of the results and conclusions. In addition, with the addition of follow-up parameters (OS and DFS), our study included more survival-related outcomes than previous studies. Our study showed greater 1-year OS and 1-year DFS rates in the NICT group, while the NICRT group showed no such results. This difference might be explained by the fact that concurrent administration of all three treatment modalities in the NICRT group significantly increased treatment-related adverse effects, resulting in patients showing no advantage in terms of survival. Wang et al. (103) reported that the incidence of preoperative grade 3-4 treatment-related adverse events was 51.2% in NICRT, which was much higher than the 19.4% in NICT. A multicenter dual-arm study conducted by Yang et al. (29) directly compared the safety of NICRT and NICT, noting that treatment-related adverse events, immune-related adverse events, and post-operative complications all had higher incidences in the NICRT group than in the NICT group. The toxicity of this treatment may ultimately result in a failure of NICRT to provide long-term survival benefits. Although this review concluded that there were no significant differences among the four different neoadjuvant treatments in terms of death within 30 days after surgery, this could be attributed to three reasons. First, the incidence of mortality within 30 days after surgery was low – close to zero, in fact – regardless of the treatment type, the differences they exhibited may not be statistically evident; second, the toxic effects of the treatment did not appear in such a short period of time and needed some time to manifest; And third, this outcome showed unstable results in both sensitivity analysis and publication bias analysis. A meta-analysis by Ge et al. (105) included 27 single-arm studies with 815 patients to explore the clinical outcomes of NICT. They reported pooled rates of R0, pCR and mPR were 98.6%, 31.4% and 48.9%, respectively, largely similar to the results obtained in our study (97.5%, 27.2%, and 51.8%, respectively). Compared to CROSS (3), which received NCRT, their R0 rate was 92.0%, which was not significantly different from the results obtained in our study and those of Ge et al. (105); however, their pCR rate was 49.0%, which was significantly higher than our results or those of Ge et al. That being said, this result is consistent with our conclusion that the pCR rate in the NCRT group was higher than that in the NICT group, while there were no significant differences in terms of R0 rate. A randomized controlled multicenter study conducted by Liu et al. (106) in 2022 reported that patients receiving NCT experienced a pCR rate of 20.8% and an mPR rate of 33.3%, consistent with our findings that the NCT group had the lowest pCR and mPR rates among the all four neoadjuvant treatments.

When we combined treatment modalities based on the inclusion or absence of immunotherapy (regardless of the presence of radiation therapy), we found that the neoadjuvant immunotherapy group had a significant advantage over the traditional neoadjuvant therapy group in terms of 1-year OS and DFS rates, while there were no significant differences between the two groups in other outcomes. It can be seen that the addition of immunotherapy can significantly prolong the survival of patients. This adds further evidence to the growing pile attesting to the benefit of immunotherapy in neoadjuvant therapy. As for the other results of the same, because the results obtained in this study were that there were no significant differences among the four different neoadjuvant treatments in R0 rates and death within 30 days after surgery, there were also no differences in the comparison between the combined groups. Regarding pCR and mPR rates, since the incidences were highest in the NCRT cohort and lowest in the NCT cohort, when these two were combined together in the traditional group, it canceled out the difference that had been seen when the four cohorts were being compared individually. This systematic review and meta-analysis also had limitations. First, as most of the studies included in this review were single-armed, potential bias may arise; second, since immunotherapy is still in the process of exploration, some studies have not yet released their final results. Moreover, survival endings could only be extracted for 1 year, as for the follow-up of long-term survival, follow-up studies are needed to report; third, there were only five RCTs in this review, and the lack of RCTs may potentially lead to bias; fourth, as previously noted, both sensitivity and publication bias analysis indicated instability in the data used for death within 30 days after surgery in this review, which prohibits rigorous conclusions from being drawn therefrom; more studies and data will be needed to verify the relevant findings of this study; fifth, due to the lack of available data, the role of effective biomarkers, for instance, combined positive score (CPS) and tumor proportion score (TPS), in neoadjuvant immunotherapy could not be investigated; Sixth, due to the inconsistent guidelines for and definitions of treatment-related adverse events in the different studies used in this meta-analysis, we were unable to properly compare them, instead focusing on the endpoints of efficacy and survival.

## 5 Conclusion

In conclusion, among the four neoadjuvant treatment modalities NICRT, NICT, NCRT, and NCT, NICRT and NCRT had the highest pCR and mPR rates. There were no significant differences in R0 rates among the four neoadjuvant treatment modalities. Adding immunotherapy to neoadjuvant therapy improved 1-year OS and DFS, with the NICT group having significantly higher longer survival according to both these metrics than any of the other three modalities. The results of this review provide a basis for future studies. Further, large multicenter RCTs and longer-term follow-ups are needed to refine these findings.

## Data availability statement

The original contributions presented in the study are included in the article/**Supplementary Material**. Further inquiries can be directed to the corresponding author.

## Author contributions

All authors read and approved the final manuscript prior to submission. WS are responsible for the conception and design of the study. HW are responsible for analysis and interpretation of data, and drafting the article and revising it. CS and XZ are responsible for acquisition of data. WD and JD is responsible for data check. All authors contributed to the article and approved the submitted version.
