# Supplementary material for: Evaluation of neoadjuvant immunotherapy and traditional neoadjuvant therapy for resectable esophageal cancer: a systematic review and single-arm and network meta-analysis
Source: Front Immunol. 2023 May 12;14:1170569. doi: 10.3389/fimmu.2023.1170569 (PMC10213267; doi:10.3389/fimmu.2023.1170569)
Supplement: Supplementary file 1 [file DataSheet_1.zip › Supplementary Materials.doc]

**Supplementary Materials**

**Supplementary Table 1.** Search Strategy

**Supplementary Table 2.** Population, Intervention, Comparison, Outcome and Study design (PICOS)

**Supplementary Table 3.** Inclusion and exclusion criteria

**Supplementary Table 4.** Data Synthesis

**Supplementary Table 5.** Clinical Characteristics

**Supplementary Table 6.** Treatment-related adverse events during neoadjuvant therapy

**Supplementary Table 7.** Surgery and postoperative complications

**Supplementary Table 8.** Therapeutic efficacy and survival outcomes

**Supplementary Table 9.** Methodological Index for Non-randomized Studies (MINORS)

**Supplementary Table 10.** Cochrane Risk of Bias for Randomized Controlled Trials

**Supplementary Table 11.** PRISMA 2020 Checklist

**Supplementary Table 12.** AMSTAR-2 Checklist

**Supplementary Table 13.** References

**Supplementary Figure 1.** Network Evidence Plot of (A) R0, (B) Pathological Complete Response (pCR), (C) Major Pathological Response (mPR), (D) Death within 30 Days after Surgery, (E) 1-year Overall Survival (OS) and (F) 1-year Disease Free Survival (DFS).

**Supplementary Figure 2.** Contribution Plot of (A) R0, (B) Pathological Complete Response (pCR), (C) Major Pathological Response (mPR), (D) Death within 30 Days after Surgery, (E) 1-year Overall Survival (OS) and (F) 1-year Disease Free Survival (DFS).

**Supplementary Figure 3.** Forest Plot of Network Meta-analysis. (A) R0, (B) Pathological Complete Response (pCR), (C) Major Pathological Response (mPR), (D) Death within 30 Days after Surgery, (E) 1-year Overall Survival (OS) and (F) 1-year Disease Free Survival (DFS).

**Supplementary Figure 4.** Surface Under the Cumulative Ranking Curve of Network Meta-analysis. (A) R0, (B) Pathological Complete Response (pCR), (C) Death within 30 Days after Surgery.

**Supplementary Figure 5.** Subgroup Analysis of R0. (A) analysis based on prospective study, (B) analysis based on retrospective study, (C) analysis based on single-arm study, (D) analysis based on dual-arm study, (E) analysis based on PD-1 drugs, (F) analysis based on PD-l1 drugs, (G) analysis based on ESCC, (H) analysis based on EAC.

**Supplementary Figure 6.** Subgroup Analysis of Pathological Complete Response (pCR). (A) analysis based on prospective study, (B) analysis based on retrospective study, (C) analysis based on single-arm study, (D) analysis based on dual-arm study, (E) analysis based on PD-1 drugs, (F) analysis based on ESCC.

**Supplementary Figure 7.** Subgroup Analysis of Major Pathological Response (mPR). (A) analysis based on prospective study, (B) analysis based on retrospective study, (C) analysis based on single-arm study, (D) analysis based on dual-arm study, (E) analysis based on PD-1 drugs, (F) analysis based on ESCC.

**Supplementary Figure 8.** Subgroup Analysis of Death within 30 Days after Surgery. (A) analysis based on prospective study, (B) analysis based on retrospective study, (C) analysis based on single-arm study, (D) analysis based on dual-arm study, (E) analysis based on PD-1 drugs, (F) analysis based on ESCC.

**Supplementary Figure 9.** Subgroup Analysis of 1-year Overall Survival (OS). (A) analysis based on prospective study, (B) analysis based on retrospective study, (C) analysis based on single-arm study, (D) analysis based on PD-1 drugs, (E) analysis based on ESCC.

**Supplementary Figure 10.** Subgroup Analysis of 1-year Disease Free Survival (DFS). (A) analysis based on prospective study, (B) analysis based on retrospective study, (C) analysis based on single-arm study, (D) analysis based on dual-arm study, (E) analysis based on PD-1 drugs, (F) analysis based on ESCC.

**Supplementary Figure 11.** Sensitivity Analysis by Omitting Each Trial One by One. (A) for the analysis of R0, (B) for the analysis of pathological complete response (pCR), (C) for the analysis of major pathological response (mPR), (D) for the analysis of death within 30 days after surgery, (E) for the analysis of 1-year overall survival (OS) and (F) for the analysis of 1-year disease free survival (DFS).

**Supplementary Figure 12.** Begg’s Test Funnel Plot of the Publication Bias. (A) for R0, (B) for pathological complete response (pCR), (C) for major pathological response (mPR), (D) for death within 30 days after surgery, (E) for 1-year overall survival (OS) and (F) for 1-year disease free survival (DFS).

**Supplementary Table 1. Search Strategy**

| **Database** | **Search Strategy** |
| --- | --- |
| Pubmed | ((((((((((((((((((((((((((((immunotherapy[Title/Abstract]) OR ("immune checkpoint inhibitors"[Title/Abstract])) OR ("programmed cell death 1 receptor"[Title/Abstract])) OR ("programmed cell death ligand 1"[Title/Abstract])) OR ("cytotoxic T lymphocyte-associated antigen-4 antigen"[Title/Abstract])) OR ("CTLA-4 antigen"[Title/Abstract])) OR (anti-CTLA-4[Title/Abstract])) OR (CTLA-4[Title/Abstract])) OR (anti-PD-1[Title/Abstract])) OR (anti-PD-L1[Title/Abstract])) OR (PD-1[Title/Abstract])) OR (PD-L1[Title/Abstract])) OR (Durvalumab[Title/Abstract])) OR (Atezolizumab[Title/Abstract])) OR (avelumab[Title/Abstract])) OR (Pembrolizumab[Title/Abstract])) OR (Nivolumab[Title/Abstract])) OR (Toripalimab[Title/Abstract])) OR (Tislelizumab[Title/Abstract])) OR (Camrelizumab[Title/Abstract])) OR (Sintilimab[Title/Abstract])) OR (Tremelimumab[Title/Abstract])) OR (Ipilimumab[Title/Abstract])) OR (Lambrolizumab[Title/Abstract])) OR (cemiplimab[Title/Abstract])) OR (PDCD1[Title/Abstract])) OR (CD274[Title/Abstract])) AND ((((Neoadjuvant[Title/Abstract]) OR (Preoperative[Title/Abstract])) OR (Perioperative[Title/Abstract])) OR (before[Title/Abstract]))) AND ((((((((((((((((((("Esophageal Neoplasms"[MeSH Terms]) OR ("Cancer of Esophagus"[Title/Abstract])) OR ("Cancer of the Esophagus"[Title/Abstract])) OR ("Esophageal Cancer"[Title/Abstract])) OR ("Esophagus Cancer"[Title/Abstract])) OR ("esophageal neoplasm"[Title/Abstract])) OR ("Esophagus Neoplasm"[Title/Abstract])) OR ("carcinoma of esophagus"[Title/Abstract])) OR ("Esophageal squamous cell carcinoma"[Title/Abstract])) OR ("Esophageal squamous carcinoma"[Title/Abstract])) OR ("Esophageal squamous cell cancer"[Title/Abstract])) OR ("Esophageal carcinoma"[Title/Abstract])) OR ("esophageal adenocarcinoma"[Title/Abstract])) OR ("esophageal malignancy"[Title/Abstract])) OR (ESCC[Title/Abstract])) OR (Esophagus[Title/Abstract])) OR (Esophageal[Title/Abstract])) OR (oesophagus[Title/Abstract])) OR (oesophageal[Title/Abstract])) |
| Embase | (immunotherapy:ti,ab,kw OR 'immune checkpoint inhibitors':ti,ab,kw OR 'programmed cell death 1 receptor':ti,ab,kw OR 'programmed cell death ligand 1':ti,ab,kw OR 'cytotoxic t lymphocyte-associated antigen-4 antigen':ti,ab,kw OR 'ctla-4 antigen':ti,ab,kw OR 'anti ctla 4':ti,ab,kw OR 'ctla 4':ti,ab,kw OR 'anti pd 1':ti,ab,kw OR 'anti pd l1':ti,ab,kw OR 'pd 1':ti,ab,kw OR 'pd l1':ti,ab,kw OR durvalumab:ti,ab,kw OR atezolizumab:ti,ab,kw OR avelumab:ti,ab,kw OR pembrolizumab:ti,ab,kw OR nivolumab:ti,ab,kw OR toripalimab:ti,ab,kw OR tislelizumab:ti,ab,kw OR camrelizumab:ti,ab,kw OR sintilimab:ti,ab,kw OR tremelimumab:ti,ab,kw OR ipilimumab:ti,ab,kw OR lambrolizumab:ti,ab,kw OR cemiplimab:ti,ab,kw OR pdcd1:ti,ab,kw OR cd274:ti,ab,kw) AND (neoadjuvant:ti,ab,kw OR preoperative:ti,ab,kw OR perioperative:ti,ab,kw OR before:ti,ab,kw) AND ('esophageal neoplasms':ti,ab,kw OR 'cancer of esophagus':ti,ab,kw OR 'cancer of the esophagus':ti,ab,kw OR 'esophageal cancer':ti,ab,kw OR 'esophagus cancer':ti,ab,kw OR 'esophageal neoplasm':ti,ab,kw OR 'esophagus neoplasm':ti,ab,kw OR 'carcinoma of esophagus':ti,ab,kw OR 'esophageal squamous cell carcinoma':ti,ab,kw OR 'esophageal squamous carcinoma':ti,ab,kw OR 'esophageal squamous cell cancer':ti,ab,kw OR 'esophageal carcinoma':ti,ab,kw OR 'esophageal adenocarcinoma':ti,ab,kw OR 'esophageal malignancy':ti,ab,kw OR escc:ti,ab,kw OR esophagus:ti,ab,kw OR esophageal:ti,ab,kw OR oesophagus:ti,ab,kw OR oesophageal:ti,ab,kw) |
| Web of Science | (((((((((((((((((((((((((((TS=(immunotherapy)) OR TS=(“immune checkpoint inhibitors”)) OR TS=(“programmed cell death 1 receptor”)) OR TS=(“programmed cell death ligand 1”)) OR TS=(“cytotoxic T lymphocyte-associated antigen-4 antigen”)) OR TS=(“CTLA-4 antigen”)) OR TS=(anti-CTLA-4)) OR TS=(CTLA-4)) OR TS=(anti-PD-1)) OR TS=(anti-PD-L1)) OR TS=(PD-1)) OR TS=(PD-L1)) OR TS=(Durvalumab)) OR TS=(Atezolizumab)) OR TS=(avelumab)) OR TS=(Pembrolizumab)) OR TS=(Nivolumab)) OR TS=(Toripalimab)) OR TS=(Tislelizumab)) OR TS=(Camrelizumab)) OR TS=(Sintilimab)) OR TS=(Tremelimumab)) OR TS=(Ipilimumab)) OR TS=(Lambrolizumab)) OR TS=(cemiplimab)) OR TS=(PDCD1)) OR TS=(CD274)) AND ((((TS=(Neoadjuvant)) OR TS=(Preoperative)) OR TS=(Perioperative)) OR TS=(before)) AND (((((((((((((((((((TS=("Esophageal Neoplasms")) OR TS=("Cancer of Esophagus")) OR TS=("Cancer of the Esophagus")) OR TS=("Esophageal Cancer")) OR TS=("Esophagus Cancer")) OR TS=(“esophageal neoplasm”)) OR TS=("Esophagus Neoplasm")) OR TS=("carcinoma of esophagus")) OR TS=("Esophageal squamous cell carcinoma")) OR TS=("Esophageal squamous carcinoma")) OR TS=(“Esophageal squamous cell cancer”)) OR TS=("Esophageal carcinoma")) OR TS=(“esophageal adenocarcinoma”)) OR TS=(“esophageal malignancy”)) OR TS=(ESCC)) OR TS=(Esophagus)) OR TS=(Esophageal)) OR TS=(oesophagus)) OR TS=(oesophageal)) |
| Cochrane Library | ((immunotherapy):ti,ab,kw OR (“immune checkpoint inhibitors”):ti,ab,kw OR (“programmed cell death 1 receptor”):ti,ab,kw OR (“programmed cell death ligand 1”):ti,ab,kw OR (“cytotoxic T lymphocyte-associated antigen-4 antigen”):ti,ab,kw OR (“CTLA-4 antigen”):ti,ab,kw OR (anti-CTLA-4):ti,ab,kw OR (CTLA-4):ti,ab,kw OR (anti-PD-1):ti,ab,kw OR (anti-PD-L1):ti,ab,kw OR (PD-1):ti,ab,kw OR (PD-L1):ti,ab,kw OR (Durvalumab):ti,ab,kw OR (Atezolizumab):ti,ab,kw OR (avelumab):ti,ab,kw OR (Pembrolizumab):ti,ab,kw OR (Nivolumab):ti,ab,kw OR (Toripalimab):ti,ab,kw OR (Tislelizumab):ti,ab,kw OR (Camrelizumab):ti,ab,kw OR (Sintilimab):ti,ab,kw OR (Tremelimumab):ti,ab,kw OR (Ipilimumab):ti,ab,kw OR (Lambrolizumab):ti,ab,kw OR (cemiplimab):ti,ab,kw OR (PDCD1):ti,ab,kw OR (CD274):ti,ab,kw) AND ((Neoadjuvant):ti,ab,kw OR (Preoperative):ti,ab,kw OR (Perioperative):ti,ab,kw OR (before):ti,ab,kw) AND (("Esophageal Neoplasms"):ti,ab,kw OR ("Cancer of Esophagus"):ti,ab,kw OR ("Cancer of the Esophagus"):ti,ab,kw OR ("Esophageal Cancer"):ti,ab,kw OR ("Esophagus Cancer"):ti,ab,kw OR (“esophageal neoplasm”):ti,ab,kw OR ("Esophagus Neoplasm"):ti,ab,kw OR ("carcinoma of esophagus"):ti,ab,kw OR ("Esophageal squamous cell carcinoma"):ti,ab,kw OR ("Esophageal squamous carcinoma"):ti,ab,kw OR (“Esophageal squamous cell cancer”):ti,ab,kw OR ("Esophageal carcinoma"):ti,ab,kw OR (“esophageal adenocarcinoma”):ti,ab,kw OR (“esophageal malignancy”):ti,ab,kw OR (ESCC):ti,ab,kw OR (Esophagus):ti,ab,kw OR (Esophageal):ti,ab,kw OR (oesophagus):ti,ab,kw OR (oesophageal):ti,ab,kw) |

**Supplementary Table 2. Population, Intervention, Comparison, Outcome and Study design (PICOS)**

| **PICOS** | **Description** |
| --- | --- |
| Population | Patients with histologically-confirmed resectable esophageal carcinoma. |
| Intervention | Neoadjuvant immunotherapy combined with chemotherapy or neoadjuvant immunotherapy combined with chemoradiotherapy followed by surgery. |
| Control | Neoadjuvant chemotherapy or neoadjuvant chemoradiotherapy followed by surgery. |
| Outcomes | R0 resection, pathological complete response (pCR), major pathological response (mPR), postoperative mortality, overall survival (OS), disease free survival (DFS). |
| Study Design | All prospective and restrospective studies. |

**Supplementary Table 3. Inclusion and exclusion criteria**

| **Inclusion criteria** | **Exclusion criteria** |
| --- | --- |
| (1) patients with resectable esophageal cancer confirmed histopathologically. | (1) unpublished data. |
| (2) studies that included patients who received neoadjuvant Immunotherapy combined with chemotherapy or chemoradiotherapy. | (2) case reports, meta-analysis, academic papers, editorials, letters, review papers, comments, and basic science articles. |
| (3) patients reveived radical surgery after neoadjuvant immune combined therapy. | (3) no correlation results. |
| (4) studies were published in any languages. | (4) studies that included other types of treatment. |
|  | (5) sample size was less than 10. |
|  | (6) patients had distant metastasis. |

**Supplementary Table 4. Data Synthesis**

R0, pathological complete response (pCR), major pathological response (mPR), overall survival (OS), disease free survival (DFS) and death within 30 days after surgery were used to evaluate the efficacy of neoadjuvant therapies. For cases where the same study was published not only in a journal but also at a conference, we combined their data as the result of the study published last in time. Heterogeneity was evaluated by I^2^ statistic. The calculation of the effect index (P) and its standard error SE (P) of binary outcomes was performed according to the formula: P=X/n; SE(P)= (P(1–P)/n)^0.5 (X is the number of occurrences of an event, n is the sample size). The incidence of events between any two groups was compared by risk ratio (RR) and 95% confidence interval (CI). The combined risk ratio (RR) and 95% confidence interval (CI) need to be converted into the incidence rate. RR=(X1/n1)/(X2/n2); CI= sqrt((1/X1)+(1/X2)-(1/n1)-(1/n2))*1.96; LCI= exp(ln(RR)-CI); UCI= exp(ln(RR)+CI). During our statistical analysis with STATA software, we perform pre-processing for event number correction. If the number of events X was 0, then both the number of events X and the sample size n were added by 0.5 or 1. If the number of events X was equal to the sample size n, then the sample size n was added by 0.5 or 1. In other cases, the data did not change. We compared every two groups through network meta-analysis, and if the two groups lack relevant data, a post-hoc two-by-two comparison of single group rates would be used. For OS and DFS rates, the number of events in the calculation of the RR value was the number of survivors rather than the number of deaths. Therefore, in the present study, for R0, pCR, mPR, OS and DFS rates, RR and 95% CI > 1 indicated that treatment was more conducive to efficacy, while RR and 95% CI < 1 indicated that treatment was more detrimental to efficacy. And for death within 30 days after surgery, the interpretation of the results was the opposite. The significance level of the results was set to P <0.05.

**Supplementary Table 5. Clinical Characteristics**

| **Author** | **Year** | **Treatment** | **Sample Size, No** | **Time Range** | **Follow up (months)** | **Location** | **TNM Stage** | **Clinical Stage** | **ICI Drugs** | **Target of action** | **ICI Dose** | **CT Regimen** | **CT Drugs Dose** | **RT Dose, Gy** | **CPS** | **TPS** |
| --- | --- | --- | --- | --- | --- | --- | --- | --- | --- | --- | --- | --- | --- | --- | --- | --- |
| M.Zhu | 2022 | NICRT | 31 | 2016.7- 2021.2 | 24.7 | EGJ:29  (93.5%) | T2:2(6.5%)  T3:29(93.5%) N0:4(12.9%)  N1:13(41.9%)  N2:12(38.7%)  N3:2(6.5%) | - | Pembrolizumab | PD-1 | At the beginning of the phase 1b portion, it was administered on day 1, 22, and 43. In the subsequent phase 2 portion, it was initiated two weeks before the initiation of chemoradiation (day -14 and 8). | TC | weekly carboplatin (AUC 2 mg/ mL/ min) and paclitaxel (50 mg/m2) on day 1, 8, 15, 22, and 29. | 41.4 | <1:11  (35.5%)  1-9:11  (35.5%)  ≥10:8  (25.8%)  uk:1  (3.2%) | - |
|  |  | NCRT | 93 | 2016.7- 2021.2 | 22.5 | - | - | - | - |  | - | TC | weekly carboplatin and paclitaxel. | 41.1- 50.4 | - | - |
| Y.Zhou | 2022 | NICT | 14 | 2017- 2021 | - | ut:1(7.1%)  mt:12(85.7%)  lt:1(7.1%) | T2:0(0%)  T3:14(100%)  T4:0(0%)  N0:1(7.1%)  N1:8(57.1%)  N2:4(28.6%)  N3:1(7.1%) | - | Toripalimab | PD-1 | 240 mg, 3 weeks a cycle,2 cycles. | TC | paclitaxel 210 mg ivgtt d1+carboplatin 500 mg ivgtt d1,most 2 cycles. | - | <10:14  (100%) | - |
|  |  | NCRT | 14 | 2017- 2021 | - | ut:1(7.1%)  mt:12(85.7%)  lt:1(7.1%) | T2:1(7.1%)  T3:9(64.3%)  T4:4(28.6%)  N0:0(0%)  N1:3(21.4%)  N2:9(64.3%)  N3:2(14.3%) | - | - | - |  | TP | paclitaxel 210 mg d1 ivgtt+cisplatin 40 mg d1-3 ivgtt,2 cycles. | 40.0 | - | - |
| Zh.Zhang | 2022 | NICT | 47 | 2019.7- 2021.3 | 14.6  (11.3-24.0) | mt:19(40.4%)  lt:25(53.2%)  both:3(6.4%) | N1:28(59.6%)  N2:19(40.6%) | 2:9  (19.1%)  3:33  (70.2%)  4:5  (10.7%) | Sintilimab | PD-1 | 200 mg, 3 weeks a cycle, 2 cycles. | TC | paclitaxel liposomes (135 mg/m²) on day 1, and carboplatin (area under the curve [AUC] of 5 mg/mL per min) on day 1,2 cycles. | - | - | - |
| Y.Zhang | 2022 | NICT | 166 | 2020.10.20- 2021.8.26 | - | - | - | - | Camrelizumab | PD-1 | - | TP | - | - | - | - |
| X.K.Zhang | 2022 | NICT | 64 | 2019.6- 2020.10 | - | ut:3(4.7%)  mt:30(46.9%)  lt:31(48.4%) | T1-2:8  (12.5%)  T3-4:56  (87.5%)  N0:20  (31.3%)  N1-2:44  (68.7%) | 2:19  (29.7%)  3:33  (51.6%)  4:12  (18.7%) | Camrelizumab | PD-1 | 200 mg, 3 weeks a cycle, 2-3 cycles. | TP/FP | Paclitaxel or Paclitaxel for Injection (Albumin Bound), which doses are generally 135–175  mg/ m2 and 260  mg/m2, respectively and platinum like Cisplatin ( 75  mg/ m2) or Lobaplatin (25–30  mg/m2) or Fluorouracils such as Xeloda (1000  mg/m2, bid) and Tegafur (40  mg, bid, as less than 1.25  m2; 50  mg, bid, as between 1.25 and 1.5  m2; 60  mg, bid, as more than 1.5  m2) every three weeks,2-3 cycles. | - | - | - |
| G.Q.Zhang | 2022 | NICT | 54 | - | - | - | - | - | Toripalimab | PD-1 | 240 mg, 2 cycles. | T+S1 | nab-paclitaxel 260 mg/ m2 for 2 cyclesand oral S-1 (d1-14, bid) for two weeks. | - | - | - |
| Y.Yu | 2022 | NICT | 79 | 2019.11- 2021.8 | - | ut:13(16.5%)  mt:53(67.0%)  lt:13(16.5%) | T2:12(15.2%)  T3:55(69.2%)  T4:12(15.2%)  N0:27(34.2%)  N+:52(65.8%) | 2:3  (3.8%)  3:74  (93.7%)  4:2  (2.5%) | Tislelizumab  Camrelizumab  Toripalimab  Keytruda  Sintilimab | PD-1 | 200 mg/m2, 2 cycles. | TP | paclitaxel (135–175 mg/m2), cisplatin (80–120 mg/m2, D1 or D1–4), or nedaplatin (80–100 mg/m2, D1 or D1–4), 2 cycles. | - | - | - |
| G.Yin | 2022 | NICT | 34 | 2019.6- 2021.1 | 14.8  (3.8-26.5) | ut:4(11.8%)  mt:18(52.9%)  lt:12(35.3%) | T2:4(11.8%)  T3:27(79.4%)  T4:3(8.8%)  N0:3(8.8%)  N1:18(52.9%)  N2:12(35.3%)  N3:1(2.9%) | 2:5  (14.7%)  3:26  (76.5%)  4:3  (8.8%) | Camrelizumab | PD-1 | 200 mg, 3 weeks a cycle, 2-5 cycles. | TC | paclitaxel was administered intravenously at a dose of 100 mg/m2 of body-surface area on days 1 and 8, and the carboplatin was administered intravenously at an area under the curve of 5 mg/mL per minute on day 1,2-5 cycles. | - | <10:23  (67.6%)  ≥10:9  (26.5%)  uk:2  (5.9%) | - |
| Y.Yang | 2022 | NICRT | 30 | 2019.5- 2020.12 | - | ut:7(23.3%)  mt:16(53.3%)  lt:5(16.7%)  EGJ:2(6.7%) | T2:1(3.3%)  T3:21(70.0%)  T4:8(26.7%)  N0:3(10.0%)  N1:8(26.7%)  N2:13(43.3%)  N3:6(20.0%) | 1:1  (3.3%)  2:3  (10.0%)  3:16  (53.3%)  4:10  (33.3%) | Pembrolizumab  Camrelizumab  Toripalimab  Tislelizumab | PD-1 | pembrolizumab 2 mg/kg or 200 mg, camrelizumab, sintilimab, and tislelizumab 200 mg, toripalimab 240 mg, 3 weeks a cycle, most 2 cycles. | TP/TC/FP | - | 41.4 | - | - |
|  |  | NICT | 299 | 2019.5- 2020.12 | - | ut:41(13.7%)  mt:129(43.1%)  lt:119(39.8%)  EGJ:10(3.3%) | T1:5(1.7%)  T2:31(10.4%)  T3:229(76.6%)  T4:34(11.3%)  N0:29(9.7%)  N1:117(39.1%)  N2:142(47.5%)  N3:11(3.7%) | 1:5  (1.7%)  2:40  (13.4%)  3:206  (68.9%)  4:48  (16.1%) | Pembrolizumab  Camrelizumab  Sintilimab  Toripalimab  Tislelizumab | PD-1 | pembrolizumab 2 mg/kg or 200 mg, camrelizumab, sintilimab, and tislelizumab 200 mg, toripalimab 240 mg, 3 weeks a cycle, most 2 cycles. | TP/TC/FP | - | - | - | - |
| W.Yang | 2022 | NICT | 23 | 2020.1.19- 2020.9.12 | 13.8  (9.7-17.6) | ut:1(4.3%)  mt:9(39.1%)  lt:13(56.5%) | - | 2:8  (34.8%)  3:15  (65.2%) | Camrelizumab | PD-1 | 200 mg, 2cycles. | TC | nab-paclitaxel (260 mg/m2) on day 1, and carbop, 2 cycles. | - | <1:7  (30.4%)  ≥1:12  (52.2%)  uk:4  (17.4%) | - |
| G.Yang | 2022 | NICT | 47 | 2020.5- 2021.12 | 12.0  (3.0-22.0) | - | - | - | Camrelizumab | PD-1 | 200 mg , 3 weeks a clcle, 3 cycles. | T+capecitabine | nab-paclitaxel 260 mg/m2 and capecitabine 1250 mg/m2, 3 weeks a clcle, 3 cycles. | - | -- | - |
| X.Yan | 2022 | NICT | 45 | 2020.9- 2021.3 | - | - | T2:7(15.6%)  T3:34(75.6%)  T4:4(8.9%)  N0:20(44.4%)  N1:15(33.3%)  N2:8(17.8%)  N3:2(4.4%) | 2:18  (40.0%)  3:24  (53.3%)  4:3  (6.7%) | Tislelizumab | PD-1 | 200 mg; BeiGene Co. Ltd, Shanghai, China), 3 weeks a cycle, 3 cycles. | TC | carboplatin (area under the curve = 5 on Day 1; Qilu Pharmaceutical Co. Ltd, China), and albumin-bound paclitaxel (130 mg/m2 on Days 1 and 8; Qilu Pharmaceutical Co. LTD, China), 3 weeks a cycle, 3 cycles. | - | <1:22  (48.9%)  ≥1,<10:11  (24.4%)  ≥10,<50:7  (15.6%)  ≥50:4  (8.9%)  uk:1  (2.2%) | <1%:36  (80.0%)  ≥1%  <50%:4  (8.9%)  ≥50%:4  (8.9%)  un:1  (2.2%) |
| X.Xu | 2022 | NICRT | 20 | 2020.7- 2022.1 | 6.0 | - | - | - | Toripalimab | PD-1 | 240 mg, 3 weeks a cycle, 2 cycles. | TP | 5 cycles of paclitaxel/cisplatin (paclitaxel 45mg/m2 and cisplatin  25 mg/m2) on days 1, 8, 15, 22, 29. | 41.4 | - | - |
| W.Xu | 2022 | NICT | 46 | - | - | t:46(100.0%) | - | 2:11  (23.9%)  3:33  (71.7%)  4:2  (4.3%) | Camrelizumab | PD-1 | 200mg, 3 weeks a cycle, 2 cycles. | TC | carboplatin (AUC = 5, day 1) and  albumin paclitaxel (100 mg/m , day 1,8), 3 weeks a cycle, 2 cycles. | - | - | - |
| L.Xu | 2022 | NICT | 314 | 2018.6- 2022.3 | - | ut:44(14.0%)  mt:102(32.5%)  lt:168(53.5%) | T1:2(0.6%)  T2:47(15.0%)  T3:248(79.0%)  T4:17(5.4%)  N0:61(19.4%)  N1:172(54.8%)  N2:76(24.2%)  N3:5(1.6%) | 2:98  (31.2%)  3:195  (62.1%)  4:21  (6.7%) | Camrelizumab  Sintilimab  Tislelizumab  Pembrolizumab | PD-1 | 200 mg 3 weeks a cycle, 2-4 cycles. | TP/TC/DP/FP | The TP regimen comprised platinum-based drugs (cisplatin or  nedaplatin: 75 mg/m2 IV Q3W; carboplatin: area under the curve (AUC) 5 Q3W) and paclitaxel (175 mg/m2 IV Q3W) or docetaxel (70 mg/m2 IV Q3W). The PF regimen consisted of platinum-based drugs (50 mg/m2 IV Q2W) and 5-fluorouracil (1000 mg/m2 IV Q2W), at least 2 cycles. | - | - | - |
|  |  | NCRT | 154 | 2018.6- 2022.3 | - | ut:22(14.3%)  mt:69(44.8%)  lt:63(40.9%) | T2:14(9.1%)  T3:126(81.8%)  T4:14(9.1%)  N0:44(28.6%)  N1:84(54.5%)  N2:26(16.9%) | 2:54  (35.1%)  3:86  (55.8%)  4:14  (9.1%) | - | - |  | TP/TC/DP/FP | The TP regimen comprised platinum-based drugs (cisplatin or nedaplatin: 25 mg/m2 IV Q1W; carboplatin: AUC 2 Q1W) and paclitaxel (50 mg/m2 IV Q1W) or docetaxel (30 mg/m2 IV Q1W), and the PF regimen consisted of platinum-based drugs (75 mg/m2 IV Q3W) and 5-fluorouracil (800 mg/m2 IV Q3W), at least 2 cycles. | 32.4- 50.4 | - | - |
| L.W.Xu | 2022 | NICT | 46 | 2020.8.11- 2021.2.8 | - | t:46(100.0%) | - | 2:13  (28.3%)  3-4:33  (71.7%) | Camrelizumab | PD-1 | - | - | - | - | <10:30  (65.2%)  ≥10:16  (34.8%) | <1%:9  (19.6%)  ≥1%:37  (80.3%) |
| X.Xiao | 2022 | NICT | 55 | 2019.9- 2021.9 | 13.0  (6.0-20.0) | ut:11(20.0%)  mt:29(52.7%)  lt:15(27.3%) | T2:2(3.6%)  T3:42(76.4%)  T4:11(20.0%)  N0:7(12.7%)  N1:36(65.5%)  N2:11(20.0%)  N3:1(1.8%) | 2:8  (14.5%)  3:36  (65.5%)  4:11  (20.0%) | Pembrolizumab  Sintilimab  Tislelizumab  Camrelizumab | PD-1 | 200 mg, 2 cycles. | TP | cisplatin (75 mg/m2 of body-surface area) and paclitaxel (175 mg/m2 body-surface area), 3 weeks a cycle, 2 cycles. | - | - | - |
|  |  | NCRT | 94 | 2019.9- 2021.9 | 15.0  (11.0-22.0) | ut:13(13.8%)  mt:62(66.0%)  lt:19(20.2%) | T2:3(3.2%)  T3:74(78.7%)  T4:17(18.1%)  N0:13(13.8%)  N1:56(59.6%)  N2:22(23.4%)  N3:3(3.2%) | 2:13  (13.8%)  3:61  (64.9%)  4:20  (21.3%) | - | - | - | TP | cisplatin (75 mg/m2 of body-surface area) and paclitaxel (175 mg/m2 body-surface area), 3 weeks a cycle, 2 cycles. | 40-50.4 | - | - |
| P.Xia | 2022 | NICT | 66 | 2019.5- 2021.5 | - | ut:12(18.2%)  mt:32(48.5%)  lt:22(33.3%) | T2:8(12.1%)  T3:29(43.9%)  T4:29(44.0%)  N0:9(13.6%)  N1:20(30.3%)  N2:35(53.0%)  N3:2(3.0%) | 2:8  (12.1%)  3:29  (43.9%)  4:29  (43.9%) | Camrelizumab | PD-1 | 200 mg, 3 weeks a cycle, 2-4 cycles. | TP/TC | The platinum-based chemotherapy regimen was 75 mg/m2 of cisplatin, area under the curve (AUC) of the plasma concentration-time curve after a single dose =5 of carboplatin, or 80 mg/m2 of nedaplatin. The paclitaxel regimen was 260 mg/m2 of albumin-bound paclitaxel,3 weeks a cycle, 2-4 cycles. | - | - | - |
| X.Wang | 2022 | NICT | 59 | 2020.1-  2021.11 | - | ut:7(12.1%)  mt:28(48.3%)  lt:23(39.7%) | T2:7(12.1%)  T3:51(87.9%)  N0:25(43.1%)  N1:25(43.1%)  N2:8(13.8%) | 2:25  (43.1%)  3:33  (56.9%) | Camrelizumab | PD-1 | 200 mg, 3 weeks a cycle, 2 cycles. | TC | carboplatin (area under the curve [AUC]=5 mg/mL/min), and nab- paclitaxel (260 mg/m2) were administered intravenously on day 1 and day 22, 2 cycles. | - | - | - |
| W.Wang | 2022 | NICT | 22 | 2020.8- 2022.1 | - | - | - | - | Pembrolizumab | PD-1 | 200 mg, 3 weeks a cycle, 4 cycles. | DP | docetaxel 75mg/m2 plus cisplatin 75mg/m2 or carboplatin area under curve 4-5,3 weeks a cycle,4 cycles. | - | - | - |
| R.Wang | 2022 | NICT | 30 | 2019.10- 2022.2 | - | - | - | - | Camrelizumab | PD-1 | 200mg, 3 weeks a cycle, 2 cycles. | DP | docetaxel (75 mg/m2 IV q3w) and carboplatin (AUC¼5w6 IV q3w), 3 weeks a cycle, 2 cycles. | - | - | - |
| N.V.Uboha | 2022 | NICRT | 22 | 2018.6- 2021.10 | - | - | - | - | Avelumab | PD-L1 | 10 mg/kg, 2 weeks a cycle, 3 cycles. | TC | 2 weeks a cycle, 2 cycles. | 41.4 | - | - |
| Y.Qiao | 2022 | NICT | 48 | 2019.1- 2021.12 | - | ut:8(16.7%)  mt:26(54.2%)  lt:14(29.2%) | - | - | Camrelizumab | PD-1 | 200 mg, 3 weeks a cycle, 2 cycles. | - | - | - | - | - |
|  |  | NCT | 206 | 2019.1- 2021.12 | - | ut:37(18.0%)  mt:101(49.0%)  lt:68(33.0%) | - | - | - | - | - | - | - | - | - | - |
| Y.Qi | 2022 | NICT | 62 | 2020.2- 2021.10 | - | - | - | - | Camrelizumab | PD-1 | 200 mg, 3 weeks a cycle, 3 cycles. | DP | - | - | - | - |
| S. Matsuda | 2022 | NICT | 12 | - | - | - | - | - | Nivolumab | PD-1 | 6 received 3 courses of Nivo 360 mg/body) on day 1 every three weeks, the other 6 received one administration of Nivo (240 mg/body) 2 weeks before chemotherapy followed by the same regimen as the first six. | DCF | DTX (70 mg/m2), CDDP (70 mg/m2) and 5-FU (750 mg/m2) on days 1–5, 3 weeks a cycle, 3 cycles. | - | - | - |
| X.Ma | 2022 | NICT | 34 | 2020.1.1- 2020.12.31 | 9.5 | mt:25(73.5%)  lt:9(26.5%) | T3:26(76.5%)  T4:8(23.5%)  N2:16(47.1%)  N3:18(52.9%) | 3:14  (41.2%)  4:20  (58.8%) | Pembrolizumab  Camrelizumab  Sintilimab  Toripalimab  Tislelizumab | PD-1 | 200 mg, 3 weeks a cycle, 2 cycles. | TP | paclitaxel (260 mg/m2) and cisplatin (75 mg/m2), 3 weeks a cycle, 2 cycles. | - | - | - |
| H.Lv | 2022 | NICT | 96 | 2019.7- 2021.8 | 8.9  (6.2-14.3) | ut:14(14.6%)  mt:46(47.9%)  lt:36(37.5%) | T2:5(5.2%)  T3:86(89.6%)  T4:5(5.2%)  N0:37(38.5%)  N1:54(56.3%)  N2:5(5.2%) | 2:40  (41.7%)  3:52  (54.2%)  4:4  (4.2%) | Sintilimab | PD-1 | 200 mg, 3 weeks a cycle, 2-4 cycles. | TP/DP | Albumin-bound Paclitaxel 260 mg/m2, Nedaplatin 80 mg/m2, Cisplatin 80 mg/m2, Docetaxel 75 mg/m2, 3 weeks a cycle, 2-4 cycles. | - | - | - |
| Jun.Liu | 2022 | NICT | 60 | 2019.11.2- 2020.12.22 | - | ut:9(15.0%)  mt:36(60.0%)  lt:15(25.0%) | T2:9(15.0%)  T3:47(78.3%)  T4:4(6.7%)  N2:55(91.7%)  N3:5(8.3%) | 3:51  (85.0%)  4:9  (15.0%) | Camrelizumab | PD-1 | 200 mg, 3 weeks a cycle, 2 cycles. | TC | the dose of nab-paclitaxel was 100 mg/m2 (day 1, 8, 15) and carboplatin (day 1) targeted at area under the curve of 5 mg/mL/min, with a 3-week administration cycle, 2 cycles. | - | <1:6  (10.0%)  ≥1:44  (73.3%)  uk:10  (16.7%) | <50%:40  (66.7%), ≥50%:10  (16.7%)  uk:10  (16.7%) |
| J.Liu | 2022 | NICT | 56 | 2020.1.17- 2020.12.8 | - | ut:1(1.8%)  mt:27(48.2%)  lt:28(50.0%) | T1:1(1.8%)  T2:14(25.0%)  T3:38(67.9%)  T4:2(3.6%)  N0:9(16.1%)  N1:22(39.3%)  N2:21(37.5%)  N3:4(7.1%) | 2:13  (23.2%)  3:38  (67.9%)  4:5  (8.9%) | Camrelizumab | PD-1 | 200 mg, 3 weeks a cycle, 2 cycles. | TP | nab-paclitaxel at 260 mg/m2 in  total on day 1 and day 8 (ie, 130 mg/m2 per day) and cisplatin at  75 mg/m2 in total on days 1 to 3 (ie, 25 mg/m2 per day) of each 21-day cycle, 2 cycles. | - | <1:15  (26.8%)  ≥1,<10:6  (10.7%)  ≥10:7  (12.5%)  uk:28  (50.0%)" | - |
| Z.Li | 2022 | NICT | 20 | 2020.1- 2021.12 | - | ut:2(10.0%)  mt:15(75.0%)  lt:3(15.0%) | T2:2(10.0%)  T3:16(80.0%)  T4:2(10.0%)  N0:5(25.0%)  N1:12(60.0%)  N2:2(10.0%)  N3:1(5.0%) | 2:3  (15.0%)  3:16  (80.0%)  4:1  (5.0%) | Sintilimab | PD-1 | 200mg for 60kg, 3mg/kg for body weight < 60kg, 3 weeks a cycle, 2-4 cycles. | TP | pacilitaxel 175mg/m2 plus cisplatin 75mg/m2, 3 weeks a cycle, 2-4 cycles.  " | - | - | - |
| Y.Li | 2022 | NICT | 32 | - | - | - | - | - | Socazolimab | PD-L1 | 5mg/kg IV, 3 weeks a cycle, 4 cycles. | TP | nab-paclitaxel (125mg/m2 IV, day 1/8) and cisplatin (75mg/m2 IV, day 1), 3 weeks a cycle, 4 cycles. | - | - | - |
|  |  | NCT | 32 | - | - | - | - | - | - | - | - | TP | nab-paclitaxel (125mg/m2 IV, day 1/8) and cisplatin (75mg/m2 IV, day 1), 3 weeks a cycle, 4 cycles. | - | - | - |
| A.H.Ko | 2022 | NICRT | 34 | - | - | mt+lt:18(52.9%)  EGJ:16(47.1%) | - | 2:3  (8.8%)  3:23  (67.6%)  4:8  (23.6%) | Sotigalimab | CD40 | 4 doses of sotiga 0.3mg/kg IV. | TC | carboplatin (AUC2)/ paclitaxel (PTX) (50 mg/m2) weekly * 5 cycles. | 50.4 | - | - |
| R.J.Kelly | 2022 | NICRT | 32 | 2017.8- 2021.7 | 30.0 | - | - | 2-3:32  (100.0%) | Nivolumab  Relatlimab | PD-1  LAG-3 | 16 received 2 cycles of induction nivolumab (240mg q2 wks) plus three additional cycles of nivolumab on week 1, 3  and 5 of CRT. The other 16 patients received 2 cycles of induction nivolumab (240mg q2 wks) and relatlimab (80mg q 2 wks) plus three additional cycles of nivolumab (240mg q 2 wks) plus relatlimab (80mg q 2 wks) on week 1, 3 and 5 of CRT. | TC | carboplatin (AUC2), paclitaxel (50mg/m2). | 41.1 | - | - |
| S.Jing | 2022 | NICT | 47 | 2013.3- 2021.4 | - | ut:4(8.5%)  mt:36(76.6%)  lt:7(14.9%) | T3:47(100%)  N0:26(55.3%)  N1:15(31.9%)  N2:6(12.8%) | 2:26  (55.3%)  3:21  (44.7%) | Pembrolizumab  Camrelizumab  Toripalimab  Sintilimab | PD-1 | 200mg, 3 weeks a cycle, 1-3 cycles. | TP/FP | 3 weeks a cycle, 1-3 cycles. | - | - | - |
|  |  | NCT | 47 | 2013.3- 2021.4 | - | ut:1(2.1%)  mt:43(91.5%)  lt:3(6.4%) | T3:47(100%)  N0:30(63.8%)  N1:13(27.7%)  N2:4(8.5%) | 2:30  (63.8%),  3:17  (36.2%) | - | - | - | TP/FP | 3 weeks a cycle, 1-3 cycles. | - | - | - |
| N.Jiang | 2022 | NICRT | 23 | 2021.1.29- 2021.11.3 | - | t:23(100.0%) | T1-2:1(4.4%)  T3-4:22(95.6%)N1-2:14(62.5%) | 2:5  (21.8%)  3-4:18  (78.2%) | Toripalimab | PD-1 | 240mg, 3 weeks a cycle, 3 cycles. | TC | paclitaxel (135 mg/m2) and carboplatin (AUC=5) on D1 every 3 weeks for two cycles. | 30.0 | - | - |
| B.Jiang | 2022 | NICT | 10 | 2020.8.1- 2021.10.1 | 5.0  (2.0-31.0) | - | - | 3:10  (100.0%) | Camrelizumab  Sintilimab  Tislelizumab | PD-1 | 200mg, 2 cycles. | TP | 2 cycles. | - | - | - |
| S.J.Huang | 2022 | NICT | 51 | 2019.3- 2021.1 | - | ut:6(11.8%)  mt:23(45.1%)  lt:21(41.2%)  EGJ:1(2.0%) | T3:31(60.8%)  T4:20(39.2%)  N0:20(39.2%)  N1:29(56.9%)  N2:2(3.9%) | 2:13  (25.5%)  3:18  (35.3%)  4:20  (39.2%) | Camrelizumab  Nivolumab  Pembrolizumab  Sintilimab  Tislelizumab | PD-1 | 3 weeks a cycle, 2-4 cycles. | TP/DP | 3 weeks a cycle, 2-4 cycles. | - | - | - |
| S.Huang | 2022 | NICT | 155 | 2019.11- 2021.6 | - | ut:19(12.3%)  mt:75(48.4%)  lt:61(39.4%) | T2:1(0.6%)  T3:24(15.5%)  T4:130(83.9%)  N0:60(38.7%)  N1:65(41.9%)  N2:24(15.5%)  N3:6(3.9%) | 3:21  (13.5%)  4:134  (86.5%) | Camrelizumab  Pembrolizumab  Sintilimab  Tislelizumab  Toripalimab  Nivolumab | PD-1 | 200 mg, 3 weeks a cycle, 2 cycles. | TP/DP | 2 cycles. | - | - | - |
| Z.Hong | 2022 | NICT | 26 | 2017.1- 2021.4 | - | ut:0(0%)  mt:17(65.4%)  lt:9(34.6%) | - | 2:5  (19.2%)  3:12  (46.2%)  4:9  (34.6%) | Camrelizumab  Pembrolizumab  Sintilimab | PD-1 | 200 mg, 3 weeks a cycle, 2 cycles. | TP | - | - | - | - |
|  |  | NCT | 52 | 2017.1- 2021.4 | - | ut:5(9.6%)  mt:28(53.8%)  lt:19(36.6%) | - | 2:10  (19.2%)  3:24  (46.2%)  4:18  (34.6%) | - | - | - | TP/FP | - | - | - | - |
| Z.N.Hong | 2022 | NICT | 32 | 2017.1- 2021.7 | - | ut:2(6.3%)  mt:29(90.6%)  lt:1(3.1%) | - | 1-2:10  (31.2%)  3-4:22  (68.8%) | Camrelizumab  Pembrolizumab  Sintilimab | PD-1 | sintilimab, pembrolizumab, camrelizumab 200 mg,  toripalimab 240 mg, 3 weeks a cycle, 2-4 cycles. | TP | cisplatin (60 mg/m2) on day 1, albumin-bound paclitaxel (125 mg/m2) on days 1 and 8, every 3 weeks (day 1), 2-4 cycles. | - | - | - |
|  |  | NCRT | 32 | 2017.1- 2021.7 | - | ut:1(3.1%)  mt:28(87.5%)  lt:3(9.4%) | - | 1-2:13  (40.6%)  3-4:19  (59.4%) | - | - | - | TP/FP | - | 40.0- 56.0 | - | - |
| W.He | 2022 | NICT | 20 | 2020.1- 2020.6 | - | mt:14(70.0%)  lt:6(30.0%) | - | 3:16  (80.0%)  4:4  (20.0%) | Toripalimab | PD-1 | 240 mg, 3 weeks a cycle, 2 cycles. | TC | - | - | - | - |
| J.Guo | 2022 | NICT | 15 | 2020.11- 2021.12 | - | - | - | - | Sintilimab | PD-1 | 200 mg, 3 weeks a cycle, 2 cycles. | TP | nab-paclitaxel 260 mg/m2, d1 and cisplatin 75 mg/m2, d1-3,3 weeks a cycle, 2 cycles. | - | - | - |
| Y.M.Gu | 2022 | NICT | 38 | 2020.1- 2021.4 | - | - | T2:3(7.9%)  T3:35(92.1%)  NO:9(23.7%)  N1:5(13.2%)  N2:24(63.2%) | 2:9  (23.7%)  3:29  (76.3%) | Pembrolizumab  Tislelizumab  Camrelizumab  Sintilimab  Toripalimab | PD-1 | pembrolizumab (2 mg/kg, i.v., q3w), tislelizumab  (200 mg, i.v., q3w), camrelizumab (200 mg, i.v., q3w), sintilimab (200 mg, i.v., q3w) and toripalimab (3 mg/kg, i.v., q2w), 2-4 cycles. | TP | paclitaxel (175 mg/m2 i.v., d1, q3w) plus cisplatin (75 mg/m2 i.v., d1, q3w), 2-4 cycles. | - | - | - |
| T.Gong | 2022 | NICT | 37 | 2019.12- 2022.2 | - | - | - | 3:30  (81.1%)  4:7  (18.9%) | Sintilimab | PD-1 | 2-3 cycles. | - | 2-3 cycles. | - | - | - |
| L.Gao | 2022 | NICT | 20 | 2019.10- 2020.10 | - | ut:2(10.0%)  mt:13(65.0%)  lt:5(25.0%) | - | - | Toripalimab | PD-1 | 240 mg, 3 weeks a cycle, 2 cycles. | DP | docetaxel (75 mg/m2) and cisplatin (60 mg/m2) were administrated  intravenously on days 1 and 22,3 weeks a cycle, 2 cycles. | - | - | - |
| J.Feng | 2022 | NICT | 285 | 2019.6- 2021.12 | - | - | T2:44(15.4%)  T3:188(66.0%)  T4:53(18.6%)  N0:50(17.5%)  N1:161(56.5%)  N2:63(22.1%)  N3:11(3.9%) | 2:80  (28.1%)  3:152  (53.3%)  4:53  (18.6%) | Nivolumab  Pembrolizumab  Camrelizumab  Tislelizumab  Sintilimab | PD-1 | nivolumab 3mg/Kg, pembrolizumab 2mg/Kg, camrelizumab, tislelizumab or sintilimab 200 mg, 3 weeks a cycle, 2 cycles. | TC | albumin paclitaxel 100mg/m2 on days 1 and 8 and carboplatin targeted at an area under the curve (AUC) of 5 mg/mL per minute on day 1, 3 weeks a cycle, 2 cycles. | - | - | - |
| H.Duan | 2022 | NICT | 18 | 2019.4- 2020.8 | 23.0  (17.8-29.9) | mt:15(83.3%)  lt:3(16.7%) | T3:17(94.4%)  T4:1(5.6%)  N0:7(38.9%)  N1:9(50.0%)  N2:2(11.1%) | 2:7  (38.9%)  3:10  (55.6%)  4:1  (5.5%) | Pembrolizumab | PD-1 | 200 mg, 3 weeks a cycle, 3 cycles. | TP/DP | Docetaxel (75m/m2, D1), Nedaplatin (80m/m2, D1), Nab-paclitaxel (260 mg/m²,D1) or Nab-paclitaxel (130 mg/m²,D1,8), 3 weeks a cycle, 3 cycles. | - | <1:4  (22.2%)  ≥1,<10:1  (5.6%)  ≥10:6  (33.3%)  uk:7  (38.9%) | - |
| Y.Dong | 2022 | NICT | 28 | 2021.8.13- 2022.2.12 | - | - | - | - | Camrelizumab | PD-1 | 200 mg, 3 weeks a cycle, 2 cycles. | TP | nab-paclitaxel (100 mg/m2, d1 and d8) and nedaplatin (75 mg/m2, d1), 3 weeks a cycle, 2 cycles. | - | - | - |
| J.Cheng | 2022 | NICT | 40 | 2020.6- 2021.9 | - | ut:12(30.0%)  mt:17(42.5%)  lt:11(27.5%) | T2:3(5.0%)  T3:37(92.5%)  T4:1(2.5%)  NO:1(2.5%)  N1:13(32.5%)  N2:25(62.5%)  N3:1(2.5%) | 2:2  (5.0%)  3:37  (92.5%)  4:1  (2.5%) | Pembrolizumab  Tislelizumab  Camrelizumab  Sintilimab  Toripalimab | PD-1 | sintilimab, pembrolizumab, camrelizumab, tislelizumab 200 mg Q3W, toripalimab 300 mg Q3W, 2-4 cycles. | TP/FP | paclitaxel (135–175 mg/m2 i.v, d1, q3w) plus cisplatin (75 mg/m2 i.v, d1, q3w), fluorouracil (at 750–1000 mg/m2 i.v, d1–4, q3w) plus cisplatin  (75–100 mg/m2 i.v, d1, q3w), 2-4 cycles. | - | - | - |
|  |  | NCRT | 109 | 2020.6- 2021.9 | - | ut:24(22.0%)  mt:55(50.5%)  lt:30(27.5%) | T2:3(2.8%)  T3:103(94.5%)  T4:3(2.8%)  NO:9(8.3%)  N1:54(49.5%)  N2:45(47.0%)  N3:1(0.9%) | 2:9  (8.3%)  3:97  (89.0%)  4:3  (2.8%) | - | - | - | TP/FP | paclitaxel (135–175 mg/m2 i.v, d1, q3w) plus cisplatin (75 mg/m2 i.v, d1, q3w), fluorouracil (at 750–1000 mg/m2 i.v, d1–4, q3w) plus cisplatin  (75–100 mg/m2 i.v, d1, q3w), 2-4 cycles. | 40.0-50.0 | - | - |
| F.Chen | 2022 | NICRT | 38 | 2019.5- 2021.9 | 18.5  (3.4-28.5) | ut:8(21.1%)  mt:16(42.1%)  lt:14(36.8%) | T2:4(10.5%)  T3:30(78.9%)  T4:4(10.5%)  NO:4(10.5%)  N1:20(52.6%)  N2:13(34.2%)  N3:1(2.6%) | 2:7  (18.4%)  3:27  (71.1%)  4:4  (10.5%) | Camrelizumab | PD-1 | 200 mg, 3 weeks a cycle, 2 cycles. | TC | paclitaxel was administered intravenously at a dose of 100 mg/m2 of body surface area on days 1 and 8, and the carboplatin was  administered intravenously at an area under the curve of 5 mg/ml per minute on day 1, 3 weeks a cycle, 2 cycles. | - | <1:22  (57.9%)  ≥1:12  (31.6%)  uk:4  (10.5%) | - |
| L.Zhao | 2021 | NICT | 30 | 2019.7- 2020.9 | - | - | - | - | Toripalimab | PD-1 | 240 mg, 2 cycles. | TP | paclitaxel was given at the dose of 150-175mg/m on day 1 and cisplatin was given at the dose of 70-75mg/m on day 1, 2 cycles. | - | <1:13  (43.3%)  ≥1:1  (3.3%)  uk:16  (53.4%) | - |
| Z.Zhang | 2021 | NICT | 40 | 2019.10.1- 2021.4.1 | 7.7  (2.0-21.0) | - | - | - | Sintilimab | PD-1 | 200 mg, 2 cycles. | TC | paclitaxel (135 mg/m2) and carboplatin (area under the curve ¼  5), 2 cycles. | - | - | - |
| Z.Y.Zhang | 2021 | NICT | 30 | 2020.7- 2021.6 | 6.0  (1.0-11.0) | ut:2(6.7%)  mt:18(60.0%)  lt:10(33.3%) | T3:27(90.0%)  T4:3(10.0%)  N1:12(40.0%)  N2:18(60.0%) | 3:27  (90.0%)  4:3  (10.0%) | Sintilimab | PD-1 | 200 mg, 3 weeks a cycle, 2 cycles. | TP | Cisplatin (60 mg/m2) on day 1, albumin-bound paclitaxel (125 mg/m2) on days 1 and 8, 3 weeks a cycle, 2 cycles. | - | - | - |
| X.Zhang | 2021 | NICT | 25 | - | - | - | - | - | Camrelizumab | PD-1 | - | T+S1 | - | - | - | - |
| P.Yang | 2021 | NICT | 16 | 2019.7.1- 2020.12.31 | - | ut:3(18.7%)  mt:8(50.0%)  lt:5(31.3%) | T2:2(12.5%)  T3:13(81.3%)  T4:1(6.3%)  NO:2(12.5%)  N1:10(62.5%)  N2:3(18.8%)  N3:1(6.3%) | 2:4  (25.0%)  3:10  (62.5%)  4:2  (12.5%) | Camrelizumab | PD-1 | 200 mg, 3 weeks a cycle, 2 cycles. | TC | paclitaxel was administered by intravenous drip at a dose of 100 mg/m2 of body-surface area on days  1 and 8, and the carboplatin was administered by intravenous drip and targeted at an area under the curve of 5 mg/ml per minute on day 1, 3 weeks a cycle, 2 cycles. | - | - | - |
| G.Z.Yang | 2021 | NICT | 12 | 2019.11- 2020.6 | - | ut:1(8.3%)  mt:6(50.0%)  lt:5(41.7%) | - | 2:2  (16.7%)  3:8  (66.6%)  4:2  (16.7%) | Camrelizumab | PD-1 | 200 mg, 3 weeks a cycle, 3 cycles. | T+S1 | nab-paclitaxel (260 mg/m2, IVGTT) on day 1 and S1 administered twice daily [40 mg for body surface area (BSA) <1.25 m2, 50 mg for BSA ≥1.25 to <1.50 m2, or 60 mg for  BSA ≥1.50 m2] on days 1 to 14, 3 weeks a cycle, 3 cycles. | - | <10:9  (75.0%)  ≥10:2  (16.7%)  uk:1(8.3%) | - |
| S.Yamamoto | 2021 | NICT | 13 | - | - | - | - | 1:2  (15.4%)  2:1  (7.7%)  3:10  (76.9%) | Nivolumab | PD-1 | 6 received 360 mg/body on day 1, 3 weeks a cycle, 2 cycles, the other 7 received one administration 240 mg/body 2 weeks before chemotherapy, followed by the same treatments as the first 6. | CF | CDDP at 80 mg/m2, 5-FU at 800 mg/m2 on days 1–5, 3 weeks a cycle, 2 cycles. | - | - | - |
| W.Xing | 2021 | NICT | 30 | 2019.7- 2020.10 | - | - | T3:22(73.3%)  T4:8(26.7%)  NO:7(23.3%)  N1:16(53.4%)  N2:7(23.3%) | 2:6  (20.0%)  3:17  (56.7%)  4:7  (23.3%) | Toripalimab | PD-1 | 240 mg, 3-4 weeks a cycle, 2 cycles. | TP | Paclitaxel was administered at a dose of 150–175 mg/m2 every cycle, and cisplatin was administered at a dose of 70–75 mg/m2, 3-4 weeks a cycle, 2 cycles. | - | <1:12  (40.0%)  ≥1:2  (6.7%)  uk:16  (53.3%)" | - |
| Y.Xiao | 2021 | NICT | 30 | 2019.11- 2020.6 | - | - | - | - | Camrelizumab | PD-1 | carrilizumab was given at 200 mg per time, 4 weeks after the first dose and then once every 2 weeks for a total of 4 doses. | Oxaliplatin + docetaxel | 50 mg • m2 oxaliplatin, intravenous injection, once every 2 weeks + 75 mg • m2 （locetaxel intravenous injection， once every 2 weeks， for a total of 4 chemotherapy sessions. | - | - | - |
|  |  | NCT | 30 | 2019.11- 2020.6 | - | - | - | - | - | - | - | Oxaliplatin + docetaxel | 50 mg • m oxaliplatin, intravenous injection, once every 2 weeks + 75 mg • m （locetaxel， intravenous injection， once every 2 weeks， for a total of 4 chemotherapy sessions. | - | - | - |
| Z.Wu | 2021 | NICT | 38 | 2019.11- 2020.12 | - | ut:3(7.9%)  mt:21(55.3%)  lt:14(36.8%) | T3:18(95.0%)  T4:1(5.3%)  N1:13(68.4%)  N2:5(26.3%)  N3:1(5.3%) | 3:13  (68.4%)  4:6  (31.6%) | Pembrolizumab  Camrelizumab  Sintilimab | PD-1 | - | TP/TC | - | - | <10:10  (26.3%)  ≥10:13  (34.2%)  uk:15  (39.5%)" | - |
| P.Wu | 2021 | NICT | 20 | 2020.4- 2020.12 | - | - | - | 1-2:3  (15.0%)  3-4:17  (85.0%) | Toripalimab  Sintilimab  Pembrolizumab  Camrelizumab  Tislelizumab | PD-1 | 2-4 cycles. | TP | 2 to 4 cycles of paclitaxel liposome (135-175 mg/m2) or albumin paclitaxel (180mg/m2) plus nedaplatin (75mg/m2) or lobaplatin (30mg/m ). | - | - | - |
| F.Wang | 2021 | NICT | 26 | 2020.2- 2020.9 | - | - | - | - | Camrelizumab | PD-1 | stage1: we administered 1  cycle of Camrelizumab for induction therapy (200 mg q2 weeks); stage2: pts  received 2 cycles of Camrelizumab (200 mg every 3 weeks) plus docetaxel and nedaplatin | DP | 2 cycles. | - | - | - |
| T.V.D.Ende | 2021 | NICRT | 40 | 2017.7- 2019.3 | 24.0 | mt:2(5.0%)  lt:31(77.5%)  EGJ:7(17.5%) | T2:10(25.0%)  T3:29(72.5%)  T4:1(2.5%)  NO:13(32.5%)  N1:18(45.0%)  N2:9(22.5%) | - | Atezolizumab | PD-L1 | intravenous atezolizumab  (1,200 mg) in the first and 4th week of nCRT. Atezolizumab monotherapy was administered after nCRT in week 7, 10, and 13. | TC | five cycles once a week, intravenous carboplatin (AUC 2 mg/mL/ minute), intravenous paclitaxel (50 mg/minute). | 41.4 | ≥1:26  (66.7%)  ≥10:17  (43.6%)  ≥25:15  (38.5%) | - |
| S.Sihag | 2021 | NICRT | 25 | 2017-2020 | - | - | T1:1(4.0%)  T2:2(8.0%)  T3:21(84.0%)  T4:1(4.0%)  NO:10(40.0%)  N1:14(56.0%)  N2:1(4.0%) | 1:1  (0.7%)  2:6  (4.2%)  3:108  (75.5%)  4:28  (19.6%) | Durvalumab | PD-L1 | 2 cycles. | FOLFOX | - | - | - | - |
|  |  | NCRT | 143 | 2017-2020 | - | - | T1:2(1.4%)  T2:12(8.4%)  T3:127(88.8%)  T4:2(1.4%)  NO:35(24.5%)  N1:80(55.9%)  N2:27(18.9%)  N3:1(0.7%) | 2:2  (8.0%)  3:22  (88.0%)  4:1  (4.0%) | - | - | - | - | - | - | - | - |
| D.Shen | 2021 | NICT | 28 | 2019.6.1- 2020.6.30 | - | ut:3(10.7%)  mt:14(50.0%)  lt:8(28.6%)  EGJ:3(10.7%) | T2:3(10.7%)  T3:23(82.1%)  T4:2(7.1%)  NO:2(7.1%)  N1:15(53.6%)  N2:9(32.1%)  N3:2(7.1%) | - | Nivolumab  Pembrolizumab  Camrelizumab | PD-1 | nivolumab at a dose of 3 mg, pembrolizumab at a dose of 2 mg per kilogram of body weight, and camrelizumab at a dose of 200 mg, 3 weeks a cycle, 2 cycles. | TC | albumin paclitaxel 100 mg per square meter of body-surface area on days 1 and 8, and carboplatin targeted at an area under the curve (AUC) of 5 mg per milliliter per minute on day 1, 3 weeks a cycle, 2 cycles. | - | - | - |
| X.Shang | 2021 | NICT | 42 | - | - | - | - | 3:42  (100%) | Pembrolizumab | PD-1 | 200 mg, 3 weeks a cycle, 3  cycles. | TP | paclitaxel (135mg/m2, IV, D2, Q3W for 3 cycles) ，and cisplatin (20mg/m2, IV, D2-4, Q3W for 3 cycles). | - | - | - |
| M.A.Shah | 2021 | NICRT | 40 | 2017.8.4- 2020.10.26 | - | - | T3-T4Nx or T2N1M0 | - | Pembrolizumab | PD-1 | - | TC | - | 41.4 | - | - |
|  |  | NCRT | 40 | 2017.8.4- 2020.10.26 | - | - | T3-T4Nx or T2N1M0 | - | - | - | - | TC | - | 41.4 | - | - |
| J.Ma | 2021 | NICT | 48 | 2020.6- 2021.8 | - | - | - | - | Camrelizumab | PD-1 | 200 mg, 3 weeks a cycle, 2-4 cycles. | TP | paclitaxel (155mg/m2, iv, q3w) and nedaplatin (80mg/m2, iv, q3w),2-4 cycles. | - | - | - |
| Lv | 2021 | NICT | 28 | 2019.7.27- 2021.1.29 | - | - | - | - | Camrelizumab | PD-1 | 200 mg, 3 weeks a cycle, 2-4 cycles. | TP | albumin paclitaxel (260 mg/m2 IV  q3w) and cisplatin (75 mg/m2 IV q3w), 2-4 cycles. | - | - | - |
| Hui.Lv | 2021 | NICT | 101 | 2019.7- 2021.8 | - | - | - | 2:41  (40.6%)  3:55  (54.5%)  4:5  (4.9%) | - | PD-1 | - | - | - | - | - | - |
| H.L.Lv | 2021 | NICT | 16 | 2019.7.27- 2020.9.26 | - | - | - | - | Camrelizumab | PD-1 | 200 mg, 3 weeks a cycle, 2-4 cycles. | TP | albumin paclitaxel (260 mg/m2 IV q3w) and cisplatin (75 mg/m2 IV q3w), 2-4 cycles. | - | - | - |
| D.Liu | 2021 | NICT | 23 | - | - | - | - | - | Toripalimab | PD-1 | 240 mg, 3 weeks a cycle, 2 cycles. | TP | PTX 175 mg/m2 + DDP 75 mg/m2 q3w, 2 cycles. | - | - | - |
| C.Li | 2021 | NICRT | 20 | 2019.1- 2019.12 | 6.6  (2.2-12.3) | ut:5(25.0%)  mt:11(55.0%)  lt:4(20.0%) | T3:16(80.0%)  T4:4(20.0%)  NO:2(10.0%)  N1:5(25.0%)  N2:10(50.0%)  N3:3(15.0%) | 2:2  (10.0%)  3:13  (65.0%)  4:5  (25.0%) | Pembrolizumab | PD-1 | 2 mg/kg, 3 weeks a cycle, 2 cycles. | TC | carboplatin (area under the curve of 2 mg per milliliter per minute) and  paclitaxel (50 mg/m2), which were administrated intravenously on days 1, 8, 15, 22 and 29. | 41.4 | - | - |
| G.Y.Ku | 2021 | NICRT | 36 | - | - | t:11(30.6%)  GEJ:25(69.4%) | T1-2:4(11.1%)  T3-4:32(88.9%)  N0:13(36.1%)  N+:23(63.9%) | - | Durvalumab | PD-L1 | 1500 mg, 4 weeks a cycle, 2 cycles. | mFOLFOX6 | - | 50.4 | - | - |
| B.Huang | 2021 | NICT | 23 | 2018.1- 2020.12 | - | ut:4(17.4%)  mt:13(56.5%)  lt:6(26.1%) | T2:4(17.4%)  T3:15(65.2%)  T4:4(17.4%)  N1:4(17.4%)  N2:14(60.9%)  N3:5(21.7%) | 2:3  (13.0%)  3:14  (60.8%)  4:6  (34.8%) | Pembrolizumab | PD-1 | 200 mg, 3 weeks a cycle, 2 cycles. | DP | docetaxel 75 mg/m2 + nidaplatin  80 mg/m2, intravenous drip, d1, 3 weeks a cycle, 2 cycles. | - | <1:7  (30.4%)  ≥1:8  (34.8%)  uk:8  (34.8%)" | - |
|  |  | NCT | 31 | 2018.1- 2020.12 | - | ut:2(6.4%)  mt:22(50.0%)  lt:7(22.6%) | T2:3(9.7%)  T3:25(80.6%)  T4:3(9.7%)  N1:10(32.3%)  N2:18(58.1%)  N3:3(9.6%) | 2:4  (12.9%)  3:22  (70.1%)  4:5  (16.1%) | - | - | - | DP | docetaxel 75 mg/m2 + nidaplatin  80 mg/m2, intravenous drip, d1, 3 weeks a cycle, 2 cycles. | - | - | - |
| Hong | 2021 | NICT | 38 | 2017.1- 2021.7 | - | ut:1(2.6%)  mt:21(55.3%)  lt:16(42.1%) | - | 1:19  (50.0%)  2:4  (10.5%)  3:15  (39.5%) | Sintilimab  Pembrolizumab  Camrelizumab | PD-1 | 200 mg, 3 weeks a cycle, 2 cycles. | TP | cisplatin (60 mg/m2) on day 1, and albumin-bound paclitaxel (125 mg/m2) on days 1 and 8, 3 weeks a cycle, 2 cycles. | - | - | - |
| H.T.Duan | 2021 | NICT | 23 | - | - | ut:1(4.3%)  mt:19(82.6%)  lt:3(13.0%) | T2:1(4.3%)  T3:20(87.0%)  T4:2(8.7%)  NO:5(21.7%)  N1:12(52.2%)  N2:6(26.1%) | 2:4  (17.4%)  3:17  (73.9%)  4:2  (8.7%) | Sintilimab | PD-1 | 200 mg, 3 weeks a cycle, 3 cycles. | DP/TP | docetaxel 75 mg/m2 on day 1, albumin-bound paclitaxel 130 mg/m2 on days 1 and 8 or 260 mg/m2 on day 1, plus nedaplatin 80 mg/m2 on day 1, 3 weeks a cycle, 3 cycles. | - | <1:2  (8.7%)  ≥1,<10:3  (13.0%)  ≥10:5  (21.7%)  uk:13  (56.6%) | - |
| C.Cheng | 2021 | NICT | 20 | 2020.1.19- 2020.7-21 | - | - | - | - | Camrelizumab | PD-1 | 200mg, 3 weeks a cycle, 2 cycles. | TC | albumin paclitaxel (260mg/m2) and carboplatin (area under the curve = 5), 3 weeks a cycle, 2 cycles. | - | - | - |
| A.Athauda | 2021 | NICT | 15 | - | - | - | T2:6(40.0%)  T3:9(60.0%)  NO:10(66.7%)  N1:1(6.7%)  N2:4(26.6%) | - | Avelumab | PD-L1 | - | FLOT | - | - | - | - |
| G.Zhang | 2020 | NICT | 24 | - | 7.9  (6.2-10.9) | - | - | - | Toripalimab | PD-1 | 2-4 cycles. | T+S1 | 2-4 cycles. | - | - | - |
| W.Qi | 2020 | NICRT | 40 | 2019.6- 2019.12 | - | ut:2(10.0%)  mt:8(40.0%)  lt:10(50.0%) | T3:14(70.0%)  T4:6(30.0%)  N1:5(25.0%)  N2:8(40.0%)  N3:7(35.0%) | 3:10  (50.0%)  4:10  (50.0%) | Pembrolizumab | PD-1 | 2mg/kg, 3 weeks a cycle, 2 cycles. | TC | carboplatin (AUC Z 2) and paclitaxel (50mg/m2) or nab-paclitaxel (60mg/m2) on day 1,8,15,22,29. | 41.4 | - | - |
| S.Y.Park | 2020 | NICRT | 16 | 2017-2018 | - | ut:4(25.0%)  mt:6(37.5%)  lt:6(37.5%) | - | - | Pembrolizumab | PD-1 | 200 mg, 3 weeks a cycle, 2 cycles. | TC | Paclitaxel at a dose of 45 mg/BSA and carboplatin targeted at an area under the curve of 2 mg per milliliter per minute were administered weekly for a total of  5 doses. | 44.1 | - | - |
|  |  | NCRT | 22 | 2017-2018 | - | ut:4(18.2%)  mt:13(59.1%)  lt:5(22.7%) | T2:5(22.7%)  T3:16(72.7%)  T4:1(4.5%)  NO:2(9.1%)  N1:7(31.8%)  N2:12(54.5%)  N3:1(4.5%) | - | - | - | - | FP | 5-fluorouracil 1000 mg/body surface area (BSA) for 4 days, and cisplatin 75 mg/BSA for 1 day every 4 weeks for two cycles. | 44.1 | - | - |
| K.Li | 2020 | NICT | 17 | - | 4.5 | - | - | - | Toripalimab | PD-1 | 240 mg, 3 weeks a cycle, 2-3 cycles. | TC | nab-paclitaxel (260 mg/m2 d1, Q3W) and carboplatin (AUC=5 d1, Q3W) before surgery, 2-3 cycles. | - | - | - |
| H.Li | 2020 | NICRT | 20 | - | - | - | - | - | Pembrolizumab | PD-1 | 2 mg/kg, 3 weeks a cycle, 2 cycles. | TC | carboplatin (AUC = 2, once a week for 5 weeks), paclitaxel (50 mg/m2, once a week for 5 weeks). | 41.4 | - | - |
| Y.Gu | 2020 | NICT | 17 | 2019.5- 2020.6 | - | - | T2:4(23.5%)  T3:13(76.5%)  NO:16(94.1%)  N1:1(5.9%) | - | Sintilimab | PD-1 | 200mg, 3 weeks a cycle, 2 cycles. | TP+S1 | ipo-paclitaxel (135 mg/m2, iv, d1), cisplatin (25mg/m2, iv, d1-3), S-1 (40mg po, bid, d1-14) were given for 2 cycles every 3wks. | - | - | - |
| S.Lee | 2019 | NICRT | 28 | - | 12.4 | - | - | 1-3 | Pembrolizumab | PD-1 | 200mg, 3 weeks a cycle, 2 cycles. | TC | - | 44.1 | - | - |

**Abbreviations: ICI, immune checkpoint inhibitor; CT, chemotherapy; RT, radiotherapy; CPS, combined positive score; TPS, tumor proportion score; NICRT, neoadjuvant immunotherapy combined with chemoradiotherapy; NICT, neoadjuvant immunochemotherapy; NCRT, neoadjuvant chemoradiotherapy; NCT, neoadjuvant chemotherapy; t, thoracic; ut, upper thoracic; mt, middle thoracic; lt, lower thoracic; EGJ, esophagogastric junction; uk, unknown.**

**Supplementary Table 6. Treatment-related adverse events during neoadjuvant therapy**

| **Author** | **Year** | **Treatment** | **Sample Size, No** | **Anemia** | **Leukopenia** | **Thrombocytopenia** | **Grade≥3**  **AE** | **Myocarditis** | **Immune pneumonia** | **Endocrinopathy** | **Rash** | **Diarrhea/ Colitis** | **RCCEP** | **Liver function injury** |
| --- | --- | --- | --- | --- | --- | --- | --- | --- | --- | --- | --- | --- | --- | --- |
| M.Zhu | 2022 | NICRT | 31 | - | - | - | 17/31 | 1/31 | 0/31 | 1/31 | 5/31 | 7/31 | - | 1/31 |
|  |  | NCRT | 93 | - | - | - | - | - | - | - | - | - | - | - |
| Y.Zhou | 2022 | NICT | 14 | - | - | - | - | - | - | - | - | - | - | - |
|  |  | NCRT | 14 | - | - | - | - | - | - | - | - | - | - | - |
| Zh.Zhang | 2022 | NICT | 47 | 26/47 | 28/47 | 19/47 | - | 1/47 | - | 3/47 | 2/47 | - | - | 5/47 |
| Y.Zhang | 2022 | NICT | 166 | - | - | - | - | - | 14/166 | - | - | - | 75/166 | - |
| X.K.Zhang | 2022 | NICT | 64 | - | - | - | - | - | - | - | - | - | - | - |
| G.Q.Zhang | 2022 | NICT | 54 | - | - | - | 10/54 | - | - | - | - | - | - | - |
| Y.Yu | 2022 | NICT | 79 | - | - | - | - | - | 5/79 | 1/79 | - | - | - | - |
| G.Yin | 2022 | NICT | 34 | 4/34 | 4/34 | 3/34 | - | - | 1/34 | 2/34 | 1/34 | 3/34 | 5/34 | 2/34 |
| Y.Yang | 2022 | NICRT | 30 | - | - | - | - | 2/30 | 5/30 | 2/30 | 5/30 | - | - | 1/30 |
|  |  | NICT | 299 | - | - | - | - | 5/299 | 9/299 | 18/299 | 30/299 | - | - | 11/299 |
| W.Yang | 2022 | NICT | 23 | 13/23 | 14/23 | 7/23 | - | - | - | 1/23 | 14/23 | 3/23 | 9/23 | 10/23 |
| G.Yang | 2022 | NICT | 47 | - | - | - | - | - | - | - | - | - | - | - |
| X.Yan | 2022 | NICT | 45 | 23/45 | 33/45 | 22/45 | 19/45 | - | - | 8/45 | 1/45 | 1/45 | - | 5/45 |
| X.Xu | 2022 | NICRT | 20 | - | 9/13 | - | 7/13 | - | 1/13 | 2/13 | - | - | - | - |
| W.Xu | 2022 | NICT | 46 | 32/46 | 14/46 | 15/46 | 7/46 | - | - | - | 12/46 | - | 7/46 | - |
| L.Xu | 2022 | NICT | 314 | - | - | - | - | - | - | - | - | - | - | - |
|  |  | NCRT | 154 | - | - | - | - | - | - | - | - | - | - | - |
| L.W.Xu | 2022 | NICT | 46 | - | - | - | 7/46 | - | - | - | - | - | - | - |
| X.Xiao | 2022 | NICT | 55 | 22/55 | - | - | 12/55 | - | - | - | - | - | - | - |
|  |  | NCRT | 94 | 21/94 | - | - | 25/94 | - | - | - | - | - | - | - |
| P.Xia | 2022 | NICT | 66 | 48/66 | 14/66 | 7/66 | 14/66 | - | - | 1/66 | 42/66 | - | - | 10/66 |
| X.Wang | 2022 | NICT | 59 | - | - | - | - | - | - | - | - | - | - | - |
| W.Wang | 2022 | NICT | 22 | - | - | - | - | - | - | - | - | - | - | - |
| R.Wang | 2022 | NICT | 30 | - | - | - | - | - | - | - | - | - | 4/30 | - |
| N.V.Uboha | 2022 | NICRT | 22 | - | 13/22 | - | - | - | - | 2/22 | - | - | - | - |
| Y.Qiao | 2022 | NICT | 48 | - | - | - | - | 0/48 | - | - | 2/48 | - | 26/48 | 17/48 |
|  |  | NCT | 206 | - | - | - | - | - | - | - | - | - | - | - |
| Y.Qi | 2022 | NICT | 62 | - | - | - | 9/62 | - | - | - | - | - | - | - |
| S. Matsuda | 2022 | NICT | 12 | - | - | - | - | - | - | - | - | - | - | - |
| X.Ma | 2022 | NICT | 34 | - | 2/34 | - | 0/34 | - | - | - | - | 8/34 | 7/34 | - |
| H.Lv | 2022 | NICT | 96 | 12/96 | 28/96 | 13/96 | 12/96 | 1/96 | 2/96 | 10/96 | - | 10/96 | - | 9/96 |
| Jun.Liu | 2022 | NICT | 60 | 51/60 | 52/60 | 35/60 | 34/60 | - | 4/60 | 5/60 | 10/60 | 4/60 | 16/60 | 25/60 |
| J.Liu | 2022 | NICT | 56 | 3/56 | 20/56 | 2/56 | 6/56 | 1/56 | - | 2/56 | 7/56 | 4/56 | 5/56 | - |
| Z.Li | 2022 | NICT | 20 | 1/20 | - | 2/20 | 2/20 | - | 3/20 | - | - | - | - | - |
| Y.Li | 2022 | NICT | 32 | - | 14/32 | - | 21/32 | - | - | - | - | - | - | - |
|  |  | NCT | 32 | - | 8/32 | - | 20/32 | - | - | - | - | - | - | - |
| A.H.Ko | 2022 | NICRT | 34 | - | - | - | 3/34 | - | - | - | - | - | - | - |
| R.J.Kelly | 2022 | NICRT | 32 | - | - | - | - | 2/9 | 2/9 | - | - | - | - | - |
| S.Jing | 2022 | NICT | 47 | - | - | - | - | - | - | - | 1/47 | - | - | 20/47 |
|  |  | NCT | 47 | - | - | - | - | - | - | - | - | - | - | - |
| N.Jiang | 2022 | NICRT | 23 | - | 21/23 | - | - | - | - | - | - | - | - | - |
| B.Jiang | 2022 | NICT | 10 | 5/10 | - | - | 0/10 | - | - | - | - | - | - | - |
| S.J.Huang | 2022 | NICT | 51 | - | - | - | - | - | - | - | - | - | - | - |
| S.Huang | 2022 | NICT | 155 | 21/155 | 33/155 | 5/155 | 45/155 | - | - | - | 44/155 | 47/155 | - | - |
| Z.Hong | 2022 | NICT | 26 | 10/26 | 4/26 | - | - | - | - | - | 1/26 | - | - | 6/26 |
|  |  | NCT | 52 | - | - | - | - | - | - | - | - | - | - | - |
| Z.N.Hong | 2022 | NICT | 32 | - | - | - | - | - | - | - | 2/32 | - | - | - |
|  |  | NCRT | 32 | - | - | - | - | - | - | - | - | - | - | - |
| W.He | 2022 | NICT | 20 | 14/20 | 9/20 | 4/20 | 4/20 | - | - | - | 2/20 | 8/20 | - | - |
| J.Guo | 2022 | NICT | 15 | - | 9/15 | - | - | - | - | - | - | 3/15 | - | - |
| Y.M.Gu | 2022 | NICT | 38 | - | 4/38 | 2/38 | - | - | - | 2/38 | 1/38 | - | - | 2/38 |
| T.Gong | 2022 | NICT | 37 | - | - | - | 7/37 | - | - | - | - | - | - | - |
| L.Gao | 2022 | NICT | 20 | 2/20 | 3/20 | - | 3/20 | 0/20 | 0/20 | 0/20 | 2/20 | 0/20 | 0/20 | 3/20 |
| J.Feng | 2022 | NICT | 285 | - | - | - | - | - | - | - | - | - | - | - |
| H.Duan | 2022 | NICT | 18 | 2/18 | 7/18 | 1/18 | 5/18 | - | 1/18 | 1/18 | 2/18 | 2/18 | - | - |
| Y.Dong | 2022 | NICT | 28 | 20/28 | - | - | - | - | - | - | - | - | - | - |
| J.Cheng | 2022 | NICT | 40 | - | - | - | 1/40 | - | - | - | - | - | - | - |
|  |  | NCRT | 109 | - | - | - | 4/109 | - | - | - | - | - | - | - |
| F.Chen | 2022 | NICRT | 38 | 25/38 | 26/38 | 13/38 | 8/38 | 0/38 | 6/38 | 6/38 | 6/38 | 8/38 | 17/38 | 12/38 |
| L.Zhao | 2021 | NICT | 30 | - | - | - | - | - | - | - | - | - | - | - |
| Z.Zhang | 2021 | NICT | 40 | 20/40 | - | 9/40 | - | - | - | - | - | - | - | - |
| Z.Y.Zhang | 2021 | NICT | 30 | 15/30 | 8/30 | - | 1/30 | - | 0/30 | - | 0/30 | 5/30 | - | 9/30 |
| X.Zhang | 2021 | NICT | 25 | - | - | - | 2/25 | - | - | - | - | - | - | - |
| P.Yang | 2021 | NICT | 16 | 2/16 | 6/16 | 2/16 | - | - | - | - | 1/16 | 2/16 | 6/16 | - |
| G.Z.Yang | 2021 | NICT | 12 | 2/12 | 0/12 | 0/12 | 0/12 | 0/12 | 0/12 | 0/12 | - | 1/12 | 11/12 | 0/12 |
| S.Yamamoto | 2021 | NICT | 13 | - | - | - | - | - | - | - | - | - | - | - |
| W.Xing | 2021 | NICT | 30 | - | 10/30 | 4/30 | - | 1/30 | - | - | - | 5/30 | - | 1/30 |
| Y.Xiao | 2021 | NICT | 30 | - | 2/30 | - | - | - | - | - | - | - | - | - |
|  |  | NCT | 30 | - | 3/30 | 1/30 | - | - | - | - | - | - | - | - |
| Z.Wu | 2021 | NICT | 38 | - | - | - | - | - | - | - | - | - | - | - |
| P.Wu | 2021 | NICT | 20 | - | - | - | - | - | - | - | - | - | - | - |
| F.Wang | 2021 | NICT | 26 | 8/26 | 2/26 | - | - | - | - | 6/26 | - | - | - | - |
| T.V.D.Ende | 2021 | NICRT | 40 | 1/40 | 2/40 | 1/40 | 17/40 | - | 3/40 | 2/40 | 2/40 | 2/40 | - | 7/40 |
| S.Sihag | 2021 | NICRT | 25 | - | - | - | - | - | 0/25 | - | 1/25 | 4/25 | - | 1/25 |
|  |  | NCRT | 143 | - | - | - | - | - | - | - | - | - | - | - |
| D.Shen | 2021 | NICT | 28 | 3/28 | 5/28 | 3/28 | 2/28 | - | - | 1/28 | 3/28 | 2/28 | 4/28 | - |
| X.Shang | 2021 | NICT | 42 | - | -- |  | 0/42 | - | - | - | - | - | - | - |
| M.A.Shah | 2021 | NICRT | 40 | - | - | -- | - | - | - | - | - | - | - | - |
|  |  | NCRT | 40 | - | - | -- | - | - | - | - | - | - | - | - |
| J.Ma | 2021 | NICT | 48 | 13/48 | - | -- | - | - | - | - | - | - | 27/48 | - |
| Lv | 2021 | NICT | 28 | - | - | -- | - | - | - | 3/28 | - | - | 10/28 | 6/28 |
| Hui.Lv | 2021 | NICT | 101 | - | - | -- | 16/101 | - | - | - | - | - | - | - |
| H.L.Lv | 2021 | NICT | 16 | - | - | -- | 0/16 | - | - | 1/16 | - | - | 3/16 | - |
| D.Liu | 2021 | NICT | 23 | - | - | -- | 2/23 | - | - | - | - | - | - | - |
| C.Li | 2021 | NICRT | 20 | 16/20 | 20/20 | 1/20 | 13/20 | - | 4/20 | - | 1/20 | 2/20 | - | - |
| G.Y.Ku | 2021 | NICRT | 36 | - | - | - | - | - | - | 1/36 | 2/36 | - | - | 1/36 |
| B.Huang | 2021 | NICT | 23 | 19/23 | 19/23 | 13/23 | 11/23 | - | 1/23 | 4/23 | 5/23 | 3/23 | - | 11/23 |
|  |  | NCT | 31 | 18/31 | 19/31 | 12/31 | 8/31 | - | - | - | - | - | - | - |
| Hong | 2021 | NICT | 38 | - | - | - | - | - | - | - | - | - | - | - |
| H.T.Duan | 2021 | NICT | 23 | 3/23 | 12/23 | - | 7/23 | - | - | 2/23 | 4/23 | 3/23 | - | - |
| C.Cheng | 2021 | NICT | 20 | - | 12/20 | - | 2/20 | - | - | - | - | - | - | - |
| A.Athauda | 2021 | NICT | 15 | - | - | - | 9/15 | - | - | - | - | - | - | - |
| G.Zhang | 2020 | NICT | 24 | - | - | - | - | - | - | - | - | - | - | - |
| W.Qi | 2020 | NICRT | 40 | 15/20 | 20/20 | 1/20 | - | - | 4/20 | - | 1/20 | - | - | 1/20 |
| S.Y.Park | 2020 | NICRT | 16 | - | - | - | - | - | - | 4/16 | 1/16 | - | - | 8/16 |
|  |  | NCRT | 22 | - | - | - | - | - | - | - | - | - | - | - |
| K.Li | 2020 | NICT | 17 | - | - | - | 2/17 | - | - | - | - | - | - | - |
| H.Li | 2020 | NICRT | 20 | - | - | - | - | - | - | - | - | - | - | - |
| Y.Gu | 2020 | NICT | 17 | - | - | - | - | - | - | - | 1/17 | - | - | - |
| S.Lee | 2019 | NICRT | 28 | - | 13/26 | - | - | - | - | - | - | - | - | 8/26 |

**Abbreviations: AE, adverse event; NICRT, neoadjuvant immunotherapy combined with chemoradiotherapy; NICT, neoadjuvant immunochemotherapy; NCRT, neoadjuvant chemoradiotherapy; NCT, neoadjuvant chemotherapy.**

**Supplementary Table 7. Surgery and postoperative complications**

| **Author** | **Year** | **Treatment** | **Sample Size, No** | **Surgical rate** | **Method of operation** | **Surgical delay, No** | **Surgical timing (days)** | **Postoperative complications, No** | **Died 30 days after surgery, No** | **Died 90 days after surgery, No** |
| --- | --- | --- | --- | --- | --- | --- | --- | --- | --- | --- |
| M.Zhu | 2022 | NICRT | 31 | 29/31 | - | 1/29 | 24 | - | - | - |
|  |  | NCRT | 93 | - | - | - | - | - | - | - |
| Y.Zhou | 2022 | NICT | 14 | 14/14 | - | - | - | - | - | - |
|  |  | NCRT | 14 | - | - | - | - | - | - | - |
| Zh.Zhang | 2022 | NICT | 47 | 45/47 | MIE (Ivor-Lewis) |  | 29.0  (26.5.0-35.0) | 5/45 | 0/45 | 0/45 |
| Y.Zhang | 2022 | NICT | 166 | 82/166 | - | - | - | - | - | - |
| X.K.Zhang | 2022 | NICT | 64 | - | - | - | - | - | - | - |
| G.Q.Zhang | 2022 | NICT | 54 | - | - | - | - | - | - | - |
| Y.Yu | 2022 | NICT | 79 | - | McKeown:65 (82.3%)  open:14 (17.7%) | - | - | - | - | - |
| G.Yin | 2022 | NICT | 34 | - | McKeown:14 (41.2%)  Ivor Lewis:20 (58.8%) | - | 12.0  (9.0-18.0) | 14/34 | 0/34 | 0/34 |
| Y.Yang | 2022 | NICRT | 30 | 26/30 | McKeown, Ivor Lewis, Sweet | - | - | 15/26 | 0/26 | 1/26 |
|  |  | NICT | 299 | 282/299 | McKeown, Ivor Lewis, Sweet | - | - | 118/282 | 0/282 | 2/282 |
| W.Yang | 2022 | NICT | 23 | 20/23 | McKeown:18 (90.0%)  open:2 (10.0%) | - | 31.0  (24.0-42.0) | 5/20 | 0/20 | 0/20 |
| G.Yang | 2022 | NICT | 47 | 42/47 | - | 0/42 | - | - | 0/42 | 0/42 |
| X.Yan | 2022 | NICT | 45 | 36/45 | McKeown:14 (38.9%)  Ivor Lewis:20 (55.7%)  Sweet:2 (5.6%) | - | 30.0  (21.0-44.0) | 28/36 | 0/36 | 1/36 |
| X.Xu | 2022 | NICRT | 20 | 13/20 | - | - | - | - | 0/13 | 0/13 |
| W.Xu | 2022 | NICT | 46 | 37/46 | - | - | 64.0  (56.0-77.0) | - | - | - |
| L.Xu | 2022 | NICT | 314 | - | McKeown:314 (100%) | - | 28.0-56.0 | 111/314 | 0/314 | - |
|  |  | NCRT | 154 | - | McKeown:154 (100%) | - | 28.0-56.0 | 60/154 | 2/154 | - |
| L.W.Xu | 2022 | NICT | 46 | 37/46 | - | - | - | - | - | - |
| X.Xiao | 2022 | NICT | 55 | - | McKeown:146 (98.0%) | - | 42.0-56.0 | - | 0/55 | - |
|  |  | NCRT | 94 | - | open:3 (2.0%) | - | 42.0-56.0 | - | 2/94 | - |
| P.Xia | 2022 | NICT | 66 | 60/66 | open:26 (43.3%)  minimally invasive:33 (55.0%)  minimally invasive to open:1 (1.7%) | - | 30.0  (28.8-33.0) | 15/60 | - | - |
| X.Wang | 2022 | NICT | 59 | 58/59 | - | - | - | - | - | - |
| W.Wang | 2022 | NICT | 22 | 14/22 | McKeown:14 (100%) | - | 44.0  (29.0-74.0) | - | - | - |
| R.Wang | 2022 | NICT | 30 | 25/30 | McKeown:20 (80.0%)  open:5 (20.0%) | - | 28.0-64.0 | - | - | - |
| N.V.Uboha | 2022 | NICRT | 22 | 19/22 | - | - | - | - | - | - |
| Y.Qiao | 2022 | NICT | 48 | - | McKeown:254(100%) | - | - | - | 0/48 | - |
|  |  | NCT | 206 | - | - | - | - | - | 3/206 | - |
| Y.Qi | 2022 | NICT | 62 | 48/62 | - | - | 28.0-42.0 | - | - | - |
| S. Matsuda | 2022 | NICT | 12 | 12/12 | - | - | - | - | - | - |
| X.Ma | 2022 | NICT | 34 | 34/34 | esophagectomy with 2-field lymphadenectomy:16(47%)  esophagectomy with 3-field lymphadenectomy:18(53%) | 0/34 | 35.0  (28.0-56.0) | 27/34 | - | 0/34 |
| H.Lv | 2022 | NICT | 96 | - | McKeown:90 (93.8%)  Ivor Lewis:6 (6.3%) | - | 37.5  (32.0-41.0) | 47/95 | 0/95 | 0/95 |
| Jun.Liu | 2022 | NICT | 60 | 51/60 | - | 8/51 | 42.0-56.0 | 24/51 | 0/51 | 0/51 |
| J.Liu | 2022 | NICT | 56 | 51/56 | McKeown:50 (98.0%)  open:1 (2.0%) | - | 42.0 | 14/51 | 0/51 | 0/51 |
| Z.Li | 2022 | NICT | 20 | 20/20 | - | - | - | - | - | - |
| Y.Li | 2022 | NICT | 32 | 29/32 | - | - | - | 2/29 | - | - |
|  |  | NCT | 32 | 28/32 | - | - | - | 2/28 | - | - |
| A.H.Ko | 2022 | NICRT | 34 | 28/34 | Ivor Lewis:28 (100.0%) | - | - | - | - | - |
| R.J.Kelly | 2022 | NICRT | 32 | 31/32 | Ivor Lewis:31 (100.0%) | - | - | - | - | - |
| S.Jing | 2022 | NICT | 47 | - | Minimally invasive esophagectomy: 35 (37.2%) | - | 40.0  (28.8-51.2) | 2/47 | - | - |
|  |  | NCT | 47 | - | open:59 (62.8%) | - | 39.5  (29.3-49.7) | 5/47 | - | - |
| N.Jiang | 2022 | NICRT | 23 | 20/23 | - | - | 42.0 | - | - | - |
| B.Jiang | 2022 | NICT | 10 | 10/10 | - | - | 28.0-42.0 | - | -- | - |
| S.J.Huang | 2022 | NICT | 51 | - | transthoracic esophagectomy with two-field lymphadenectomies:51 (100%) | - | - | - | - | - |
| S.Huang | 2022 | NICT | 155 | 116/155 | McKeown， Ivor Lewis | - | - | - | 1/116 | - |
| Z.Hong | 2022 | NICT | 26 | - | McKeown:75 (96.2%) | - | - | - | 0/26 | - |
|  |  | NCT | 52 | - | open:3 (3.8%) | - | - | - | 0/52 | - |
| Z.N.Hong | 2022 | NICT | 32 | - | McKeown:64 (100%) | - | 28.0-56.0 | - | 0/32 | - |
|  |  | NCRT | 32 | - | - | - | 28.0-56.0 | - | 1/32 | - |
| W.He | 2022 | NICT | 20 | 16/20 | McKeown:16 (100%) | 0/16 | 61.5  (55.0-71.0) | - | -- | - |
| J.Guo | 2022 | NICT | 15 | 11/15 | - | 0/11 | - | - | - | - |
| Y.M.Gu | 2022 | NICT | 38 | 37/38 | McKeown:36 (97.3%)  open:1 (2.7%) | - | 63.0  (40.0-147.0) | 12/37 | 0/37 | - |
| T.Gong | 2022 | NICT | 37 | - | - | - | - | - | - | - |
| L.Gao | 2022 | NICT | 20 | 12/20 | McKeown:12 (100%) | 0/12 | 33.2  (24.7-41.7) | - | 0/12 | - |
| J.Feng | 2022 | NICT | 285 | - | McKeown:216 (75.8%)  Ivor Lewis:69 (24.2%) | - | - | - | - | - |
| H.Duan | 2022 | NICT | 18 | 13/18 | McKeown， Ivor Lewis | - | 28.0-42.0 | - | 1/13 | - |
| Y.Dong | 2022 | NICT | 28 | 24/28 | - | - | 28.0-42.0 | - | - | - |
| J.Cheng | 2022 | NICT | 40 | - | McKeown:149 (100%) | - | 67.8  (45.3-90.4) | 14/40 | 0/40 | 0/40 |
|  |  | NCRT | 109 | - | - | - | 80.0  (48.3-111.8) | 68/109 | 3/109 | 5/109 |
| F.Chen | 2022 | NICRT | 38 | - | McKeown:16 (42.1%)  Ivor Lewis:22 (57.9%) | - | 31.0  (18.0-46.0) | - | - | - |
| L.Zhao | 2021 | NICT | 30 | 24/30 | - | - | 28.0-42.0 | - | - | - |
| Z.Zhang | 2021 | NICT | 40 | 40/45 | - | - | - | - | - | - |
| Z.Y.Zhang | 2021 | NICT | 30 | 23/30 | McKeown:23 (100%) | 0/23 | 40.0  (31.5-48.5) | - | 0/23 | - |
| X.Zhang | 2021 | NICT | 25 | 25/25 | - | 0/25 | - | - | - | - |
| P.Yang | 2021 | NICT | 16 | 16/16 | MIE, open | - | - | - | - | - |
| G.Z.Yang | 2021 | NICT | 12 | - | MIE:12 (100%) | - | 8.4  (2.8-20.3) | - | - | - |
| S.Yamamoto | 2021 | NICT | 13 | 13/13 | - | - | - | - | - | - |
| W.Xing | 2021 | NICT | 30 | 24/30 | McKeown， Ivor Lewis | - | - | - | 2/24 | - |
| Y.Xiao | 2021 | NICT | 30 | 30/30 | MIE:30(100%) | - | - | 7/30 | - | - |
|  |  | NCT | 30 | 30/30 | MIE:30(100%) | - | - | 9/30 | - | - |
| Z.Wu | 2021 | NICT | 38 | - | McKeown:17 (44.7%)  Ivor Lewis:21 (55.3%) | - | 35.0  (14.0-91.0) | 10/38 | - | - |
| P.Wu | 2021 | NICT | 20 | 13/20 | - | - | - | - | - | - |
| F.Wang | 2021 | NICT | 26 | 12/17 | - | - | 28.0-42.0 | - | - | - |
| T.V.D.Ende | 2021 | NICRT | 40 | 33/40 | MIE:33 (100%) | 0/33 | 7.0-21.0 | - | 0/33 | 0/33 |
| S.Sihag | 2021 | NICRT | 25 | 25/33 | McKeown:4(2.4%) | - | 54.0  (47.0-61.0) | - | 0/25 | 0/25 |
|  |  | NCRT | 143 | - | Ivor Lewis:164(97.6%) | - | 53.0  (47.0-66.0) | - | 2/143 | 3/143 |
| D.Shen | 2021 | NICT | 28 | 27/28 | MIE:15 (55.6%)  open:4 (14.8%)  both:8 (29.6%) | - | - | - | - | - |
| X.Shang | 2021 | NICT | 42 | 29/42 | McKeown:29 (100%) | 0/29 | 28.0-42.0 | - | - | - |
| M.A.Shah | 2021 | NICRT | 40 | - | - | - | - | - | - | - |
|  |  | NCRT | 40 | - | - | - | - | - | - | - |
| J.Ma | 2021 | NICT | 48 | 20/30 | - | - | - | - | - | - |
| Lv | 2021 | NICT | 28 | - | - | - | - | - | - | - |
| Hui.Lv | 2021 | NICT | 101 | - | - | - | - | - | - | - |
| H.L.Lv | 2021 | NICT | 16 | - | - | 0/16 | 28.0-42.0 | - | - | - |
| D.Liu | 2021 | NICT | 23 | 18/23 | - | - | 28.0-42.0 | - | - | - |
| C.Li | 2021 | NICRT | 20 | 18/20 | MIE:10 (55.6%)  open:8 (44.4%) | 0/20 | 42.5  (32.0-64.0) | - | 0/18 | 0/18 |
| G.Y.Ku | 2021 | NICRT | 36 | 25/36 | - | - | 28.0-42.0 | - | 0/25 | 1/25 |
| B.Huang | 2021 | NICT | 23 | 21/23 | MIE:21 (100%) | - | 28.0-42.0 | - | - | - |
|  |  | NCT | 31 | 27/31 | MIE:27 (100%) | - | 28.0-42.0 | - | - | - |
| Hong | 2021 | NICT | 38 | - | MIE:38 (100%) | - | 46.5  (27.4-65.6) | - | 0/38 | - |
| H.T.Duan | 2021 | NICT | 23 | 17/23 | McKeown:15 (88.2%)  Ivor Lewis:1 (5.9%)  Sweet:1 (5.9%) | 0/17 | 28.0-42.0 | - | 0/17 | 0/17 |
| C.Cheng | 2021 | NICT | 20 | 18/20 | - | - | 42.0 | - | - | - |
| A.Athauda | 2021 | NICT | 15 | 15/15 | - | 0/15 | 44.8 | 8/14 | - | - |
| G.Zhang | 2020 | NICT | 24 | 18/24 | - | - | - | - | - | - |
| W.Qi | 2020 | NICRT | 40 | 14/20 | - | - | 42.0 | - | - | - |
| S.Y.Park | 2020 | NICRT | 16 | 16/16 | - | 0/16 | 42.0-56.0 | 11/16 | - | - |
|  |  | NCRT | 22 | - | - | - | 42.0-56.0 | 13/22 | - | - |
| K.Li | 2020 | NICT | 17 | 12/15 | - | 0/15 | - | - | - | - |
| H.Li | 2020 | NICRT | 20 | 15/20 | - | - | 28.0-42.0 | - | - | - |
| Y.Gu | 2020 | NICT | 17 | 15/17 | - | - | - | - | - | - |
| S.Lee | 2019 | NICRT | 28 | 26/28 | - | - | - | - | - | - |

**Abbreviations: NICRT, neoadjuvant immunotherapy combined with chemoradiotherapy; NICT, neoadjuvant immunochemotherapy; NCRT, neoadjuvant chemoradiotherapy; NCT, neoadjuvant chemotherapy; MIE, minimal invasive esophagectomy.**

**Supplementary Table 8. Therapeutic efficacy and survival outcomes**

| **Author** | **Year** | **Treatment** | **Sample Size, No** | **R0 resection rate** | **pCR** | **mPR** | **ORR** | **DCR** | **mOS (months)** | **1-year OS** | **2-year OS** | **mDFS (months)** | **1-year DFS** | **1-year DFS** |
| --- | --- | --- | --- | --- | --- | --- | --- | --- | --- | --- | --- | --- | --- | --- |
| M.Zhu | 2022 | NICRT | 31 | 28/31 | 7/31 | - | - | - | not reached | - | 21/31 | 19.6 | - | 15/31 |
|  |  | NCRT | 93 | - | 12/93 | - | - | - | not reached | - | 60/93 | 14.6 | - | 30/93 |
| Y.Zhou | 2022 | NICT | 14 | - | 3/14 | - | 9/14 | - | - | - | - | - | - | - |
|  |  | NCRT | 14 | - | - | - | 10/14 | - | - | - | - | - | - | - |
| Zh.Zhang | 2022 | NICT | 47 | 44/45 | 10/45 | 20/45 | - | - | not reached | 41/45 | 41/45 | not reached | 31/45 | 26/45 |
| Y.Zhang | 2022 | NICT | 166 | 79/82 | 15/81 | 51/81 | 100/141 | - | not reached | - | - | - | - | - |
| X.K.Zhang | 2022 | NICT | 64 | - | 27/64 | - | - | - | - | - | - | - | - | - |
| G.Q.Zhang | 2022 | NICT | 54 | - | 16/54 | 26/54 | - | - | - | - | - | - | - | - |
| Y.Yu | 2022 | NICT | 79 | - | - | - | - | - | - | - | - | - | - | - |
| G.Yin | 2022 | NICT | 34 | 32/34 | 12/34 | 17/34 | - | - | not reached | 32/34 | 28/34 | not reached | 29/34 | 24/34 |
| Y.Yang | 2022 | NICRT | 30 | - | 11/26 | - | - | - | - | - | - | - | - | - |
|  |  | NICT | 299 | - | 72/282 | - | - | - | - | - | - | - | - | - |
| W.Yang | 2022 | NICT | 23 | 20/20 | 5/20 | 10/20 | 18/20 | 20/20 | - | - | - | not reached | 16/20 | - |
| G.Yang | 2022 | NICT | 47 | 42/42 | 14/42 | 27/42 | 35/42 | - | - | 41/42 | - | - | 39/42 | - |
| X.Yan | 2022 | NICT | 45 | 29/36 | 18/36 | 26/36 | - | - | - | - | - | - | - | - |
| X.Xu | 2022 | NICRT | 20 | - | 7/13 | 10/13 | - | - | - | - | - | - | - | - |
| W.Xu | 2022 | NICT | 46 | 37/37 | 8/37 | 18/37 | - | - | - | - | - | - | - | - |
| L.Xu | 2022 | NICT | 314 | 285/314 | 90/314 | 166/314 | - | - | - | - | - | - | - | - |
|  |  | NCRT | 154 | 139/154 | 55/154 | 102/154 | - | - | - | - | - | - | - | - |
| L.W.Xu | 2022 | NICT | 46 | - | 8/37 | 18/37 | - | - | - | - | - | - | - | - |
| X.Xiao | 2022 | NICT | 55 | 53/55 | 17/55 | - | - | - | - | 52/55 | 41/55 | - | 47/55 | - |
|  |  | NCRT | 94 | 91/94 | 48/94 | - | - | - | - | 81/94 | 73/94 | - | 71/97 | - |
| P.Xia | 2022 | NICT | 66 | 59/60 | 4/60 | 12/60 | - | - | - | - | - | - | - | - |
| X.Wang | 2022 | NICT | 59 | - | 19/58 | - | - | - | - | - | - | - | - | - |
| W.Wang | 2022 | NICT | 22 | - | 4/14 | - | - | - | - | - | - | - | - | - |
| R.Wang | 2022 | NICT | 30 | 25/25 | 5/25 | - | - | - | - | - | - | - | - | - |
| N.V.Uboha | 2022 | NICRT | 22 | 15/19 | 5/19 | - | - | - | - | - | - | - | - | - |
| Y.Qiao | 2022 | NICT | 48 | - | 20/48 | 29/48 | - | - | - | - | - | - | - | - |
|  |  | NCT | 206 | - | 22/206 | 56/206 | - | - | - | - | - | - | - | - |
| Y.Qi | 2022 | NICT | 62 | 48/48 | 19/48 | 37/48 | - | - | - | - | - | - | - | - |
| S. Matsuda | 2022 | NICT | 12 | 11/12 | 4/12 | - | - | - | - | - | - | - | - | - |
| X.Ma | 2022 | NICT | 34 | 32/34 | 11/34 | - | - | - | - | - | - | - | 27/34 | - |
| H.Lv | 2022 | NICT | 96 | 95/96 | 29/95 | 60/95 | - | - | - | - | - | not reached | 91/95 | 90/95 |
| Jun.Liu | 2022 | NICT | 60 | 50/51 | 20/51 | 35/51 | - | - | - | - | - | - | - | - |
| J.Liu | 2022 | NICT | 56 | 51/51 | 18/51 | 12/51 | 34/51 | 51/51 | not reached | 50/51 | - | not reached | 48/51 | - |
| Z.Li | 2022 | NICT | 20 | 20/20 | 3/20 | 7/20 | - | - | - | - | - | - | - | - |
| Y.Li | 2022 | NICT | 32 | 29/29 | 12/29 | 20/29 | - | - | - | - | - | - | - | - |
|  |  | NCT | 32 | 28/29 | 8/29 | 18/29 | - | - | - | - | - | - | - | - |
| A.H.Ko | 2022 | NICRT | 34 | - | 10/28 | 18/28 | 26/28 | 26/28 | - | - | - | - | - | - |
| R.J.Kelly | 2022 | NICRT | 32 | - | 9/31 | 16/31 | - | - | - | - | - | 35.4 | 25/31 | - |
| S.Jing | 2022 | NICT | 47 | 41/47 | 8/47 | - | - | - | - | 45/47 | 39/47 | - | 45/47 | 38/47 |
|  |  | NCT | 47 | 43/47 | 1/47 | - | - | - | - | 40/47 | 34/47 | - | 36/47 | 30/47 |
| N.Jiang | 2022 | NICRT | 23 | - | 11/20 | 16/20 | 20/20 | 20/20 | - | - | - | - | - | - |
| B.Jiang | 2022 | NICT | 10 | 10/10 | 4/10 | 6/10 | 2/10 | 10/10 | - | - | - | - | - | - |
| S.J.Huang | 2022 | NICT | 51 | - | 11/51 | - | - | - | - | 49/51 | 31/51 | - | - | - |
| S.Huang | 2022 | NICT | 155 | 109/116 | 26/116 | 67/116 | 98/155 | 143/155 | - | - | - | - | 108/116 | 104/116 |
| Z.Hong | 2022 | NICT | 26 | - | 5/26 | - | - | - | - | - | - | - | - | - |
|  |  | NCT | 52 | - | 3/52 | - | - | - | - | - | - | - | - | - |
| Z.N.Hong | 2022 | NICT | 32 | - | 6/32 | 5/32 | - | - | - | - | - | - | - | - |
|  |  | NCRT | 32 | - | 14/32 | 9/32 | - | - | - | - | - | - | - | - |
| W.He | 2022 | NICT | 20 | 14/16 | 3/16 | 7/16 | - | - | - | - | - | - | - | - |
| J.Guo | 2022 | NICT | 15 | - | 5/11 | 6/11 | 5/8 | 8/8 | - | - | - | - | - | - |
| Y.M.Gu | 2022 | NICT | 38 | 36/37 | 9/37 | - | - | - | - | - | - | - | - | - |
| T.Gong | 2022 | NICT | 37 | 36/37 | 5/37 | 18/37 | - | - | - | - | - | - | - | - |
| L.Gao | 2022 | NICT | 20 | 12/12 | 2/12 | 5/12 | - | - | - | - | - | - | - | - |
| J.Feng | 2022 | NICT | 285 | - | 84/285 | - | - | - | - | - | - | - | - | - |
| H.Duan | 2022 | NICT | 18 | 11/13 | 6/13 | 9/13 | 9/18 | 12/13 | not reached | 11/13 | 8/13 | - | - | - |
| Y.Dong | 2022 | NICT | 28 | - | 10/24 | 16/24 | - | - | - | - | - | - | - | - |
| J.Cheng | 2022 | NICT | 40 | - | 15/40 | - | - | - | - | - | - | - | - | - |
|  |  | NCRT | 109 | - | 40/109 | - | - | - | - | - | - | - | - | - |
| F.Chen | 2022 | NICRT | 38 | 36/38 | 15/38 | 19/38 | 24/38 | 38/38 | not reached | 33/38 | 29/38 | not reached | 30/38 | 28/38 |
| L.Zhao | 2021 | NICT | 30 | - | 5/30 | - | - | - | - | - | - | - | - | - |
| Z.Zhang | 2021 | NICT | 40 | 39/40 | 10/40 | 19/40 | 33/40 | 40/40 | - | - | - | - | - | - |
| Z.Y.Zhang | 2021 | NICT | 30 | 23/23 | 4/23 | 12/23 | - | - | - | - | - | - | - | - |
| X.Zhang | 2021 | NICT | 25 | - | 8/25 | 8/25 | - | - | - | - | - | - | - | - |
| P.Yang | 2021 | NICT | 16 | 15/16 | 5/16 | - | 13/16 | 16/16 | 19.2 | 15/16 | - | 18.3 | 13/16 | - |
| G.Z.Yang | 2021 | NICT | 12 | 12/12 | 4/12 | 5/12 | 12/12 | 12/12 | - | - | - | - | - | - |
| S.Yamamoto | 2021 | NICT | 13 | 12/13 | 2/6 | - | - | - | - | - | - | - | - | - |
| W.Xing | 2021 | NICT | 30 | 24/24 | 5/24 | - | - | - | - | - | - | - | - | - |
| Y.Xiao | 2021 | NICT | 30 | 30/30 | - | - | - | - | - | - | - | - | - | - |
|  |  | NCT | 30 | 30/30 | - | - | - | - | - | - | - | - | - | - |
| Z.Wu | 2021 | NICT | 38 | 35/38 | 13/38 | 16/38 | - | - | - | - | - | - | - | - |
| P.Wu | 2021 | NICT | 20 | 12/13 | 2/13 | - | 12/17 | 17/17 | - | - | - | - | - | - |
| F.Wang | 2021 | NICT | 26 | 12/12 | 3/12 | 5/12 | 8/12 | 12/12 | - | - | - | - | - | - |
| T.V.D.Ende | 2021 | NICRT | 40 | 33/33 | 10/33 | 15/33 | - | - | 29.7 | 36/40 | 26/40 | 19.4 | 32/40 | 14/40 |
| S.Sihag | 2021 | NICRT | 25 | - | - | - | - | - | - | - | - | - | - | - |
|  |  | NCRT | 143 | - | - | - | - | - | - | - | - | - | - | - |
| D.Shen | 2021 | NICT | 28 | 26/27 | 9/27 | - | - | - | - | - | - | - | - | - |
| X.Shang | 2021 | NICT | 42 | 29/29 | 12/29 | 21/29 | 28/29 | - | - | - | - | - | - | - |
| M.A.Shah | 2021 | NICRT | 40 | - | - | 16/31 | - | - | - | - | - | - | 21/31 | - |
|  |  | NCRT | 40 | - | - | - | - | - | - | - | - | - | - | - |
| J.Ma | 2021 | NICT | 48 | - | 7/20 | - | 20/30 | 30/30 | - | - | - | - | - | - |
| Lv | 2021 | NICT | 28 | 28/28 | 9/28 | 15/28 | 22/28 | - | - | - | - | not reached | - | - |
| Hui.Lv | 2021 | NICT | 101 | 100/101 | 29/101 | - | - | - | - | - | - | - | - | - |
| H.L.Lv | 2021 | NICT | 16 | 16/16 | 7/16 | 12/16 | 14/16 | - | - | - | - | not reached | - | - |
| D.Liu | 2021 | NICT | 23 | 18/18 | 6/18 | - | - | - | - | - | - | - | - | - |
| C.Li | 2021 | NICRT | 20 | 17/18 | 10/18 | 16/18 | - | - | - | - | - | - | - | - |
| G.Y.Ku | 2021 | NICRT | 36 | - | 6/25 | 20/25 | - | - | - | - | - | - | - | - |
| B.Huang | 2021 | NICT | 23 | 21/21 | 7/23 | - | 20/23 | 22/23 | - | - | - | - | - | - |
|  |  | NCT | 31 | 26/27 | 3/31 | - | 17/31 | 27/31 | - | - | - | - | - | - |
| Hong | 2021 | NICT | 38 | 38/38 | 7/38 | 16/38 | 21/38 | 38/38 | - | - | - | - | - | - |
| H.T.Duan | 2021 | NICT | 23 | 16/17 | 6/17 | 9/17 | 5/8 | 7/8 | - | 17/17 | 16/17 | 13.8 | 16/17 | 15/17 |
| C.Cheng | 2021 | NICT | 20 | 18/18 | 5/18 | 8/18 | 17/20 | 20/20 | - | - | - | - | - | - |
| A.Athauda | 2021 | NICT | 15 | 15/15 | - | - | - | - | - | - | - | - | - | - |
| G.Zhang | 2020 | NICT | 24 | - | 3/18 | 9/18 | 19/24 | 24/24 | - | - | - | - | - | - |
| W.Qi | 2020 | NICRT | 40 | - | 9/14 | - | - | - | - | - | - | - | - | - |
| S.Y.Park | 2020 | NICRT | 16 | 16/16 | - | - | - | - | - | - | - | - | - | - |
|  |  | NCRT | 22 | 22/22 | - | - | - | - | - | - | - | - | - | - |
| K.Li | 2020 | NICT | 17 | 12/12 | 2/16 | 7/12 | - | - | - | - | - | - | - | - |
| H.Li | 2020 | NICRT | 20 | - | 9/17 | - | - | - | - | - | - | - | - | - |
| Y.Gu | 2020 | NICT | 17 | 15/15 | 4/15 | 8/15 | - | - | - | - | - | - | - | - |
| S.Lee | 2019 | NICRT | 28 | - | 12/26 | - | - | - | not reached | 21/26 | - | - | - | - |

**Abbreviations: NICRT, neoadjuvant immunotherapy combined with chemoradiotherapy; NICT, neoadjuvant immunochemotherapy; NCRT, neoadjuvant chemoradiotherapy; NCT, neoadjuvant chemotherapy; pCR, pathological complete response; mPR, major pathological response; ORR, objective response rate; DCR, disease control rate; OS, overall survival; DFS, disease free survival.**

**Supplementary Table 9. Methodological Index for Non-randomized Studies (MINORS)**

| **Study** | **Q1** | **Q2** | **Q3** | **Q4** | **Q5** | **Q6** | **Q7** | **Q8** | **Q9-12** | **Overall Score** |
| --- | --- | --- | --- | --- | --- | --- | --- | --- | --- | --- |
| M.Zhu 2022 | 2 | 1 | 2 | 2 | 2 | 2 | 0 | 2 | 8 | 21 |
| Y.Zhou 2022 | 2 | 2 | 2 | 2 | 1 | 1 | 2 | 2 | 8 | 24 |
| Zh.Zhang 2022 | 2 | 2 | 2 | 2 | 1 | 2 | 2 | 2 | / | 15 |
| Y.Zhang 2022 | 2 | 1 | 2 | 2 | 1 | 1 | 0 | 0 | / | 9 |
| X.K.Zhang 2022 | 2 | 2 | 2 | 2 | 1 | 1 | 0 | 2 | / | 12 |
| G.Q.Zhang 2022 | 2 | 1 | 2 | 2 | 1 | 1 | 0 | 0 | / | 9 |
| Y.Yu 2022 | 2 | 2 | 2 | 2 | 1 | 1 | 0 | 2 | / | 12 |
| G.Yin 2022 | 2 | 2 | 2 | 2 | 1 | 2 | 0 | 0 | / | 11 |
| Y.Yang 2022 | 2 | 2 | 2 | 2 | 1 | 1 | 0 | 0 | 8 | 18 |
| W.Yang 2022 | 2 | 2 | 2 | 2 | 1 | 2 | 2 | 1 | / | 14 |
| G.Yang 2022 | 2 | 1 | 2 | 2 | 1 | 2 | 0 | 0 | / | 10 |
| X.Yan 2022 | 2 | 2 | 2 | 2 | 1 | 1 | 1 | 0 | / | 11 |
| X.Xu 2022 | 2 | 1 | 2 | 2 | 1 | 2 | 1 | 0 | / | 11 |
| W.Xu 2022 | 2 | 1 | 2 | 2 | 1 | 2 | 2 | 0 | / | 11 |
| L.Xu 2022 | 2 | 2 | 2 | 2 | 2 | 1 | 0 | 0 | 8 | 19 |
| L.W.Xu 2022 | 2 | 2 | 2 | 2 | 1 | 2 | 1 | 0 | / | 12 |
| X.Xiao 2022 | 2 | 2 | 2 | 2 | 1 | 2 | 1 | 0 | 8 | 20 |
| P.Xia 2022 | 2 | 1 | 2 | 2 | 1 | 1 | 2 | 0 | / | 11 |
| X.Wang 2022 | 2 | 2 | 2 | 2 | 2 | 1 | 1 | 0 | / | 12 |
| W.Wang 2022 | 2 | 1 | 2 | 2 | 1 | 1 | 1 | 0 | / | 10 |
| R.Wang 2022 | 2 | 1 | 2 | 2 | 1 | 1 | 1 | 0 | / | 10 |
| N.V.Uboha 2022 | 2 | 1 | 2 | 2 | 1 | 1 | 1 | 0 | / | 10 |
| Y.Qiao 2022 | 2 | 2 | 2 | 2 | 1 | 1 | 1 | 0 | 8 | 19 |
| Y.Qi 2022 | 2 | 1 | 2 | 2 | 1 | 1 | 1 | 0 | / | 10 |
| S. Matsuda 2022 | 2 | 1 | 2 | 2 | 1 | 1 | 2 | 0 | / | 11 |
| X.Ma 2022 | 2 | 1 | 2 | 2 | 1 | 2 | 1 | 1 | / | 12 |
| H.Lv 2022 | 2 | 1 | 2 | 2 | 1 | 2 | 1 | 1 | / | 12 |
| Jun.Liu 2022 | 2 | 1 | 2 | 2 | 1 | 1 | 1 | 0 | / | 10 |
| J.Liu 2022 | 2 | 2 | 2 | 2 | 2 | 1 | 1 | 2 | / | 14 |
| Z.Li 2022 | 2 | 1 | 2 | 2 | 1 | 1 | 1 | 0 | / | 10 |
| A.H.Ko 2022 | 2 | 1 | 2 | 2 | 1 | 1 | 1 | 0 | / | 10 |
| R.J.Kelly 2022 | 2 | 1 | 2 | 2 | 1 | 2 | 1 | 2 | / | 13 |
| S.Jing 2022 | 2 | 1 | 2 | 2 | 1 | 1 | 1 | 0 | 8 | 18 |
| N.Jiang 2022 | 2 | 1 | 2 | 2 | 1 | 1 | 1 | 0 | / | 10 |
| B.Jiang 2022 | 2 | 1 | 2 | 2 | 1 | 2 | 1 | 0 | / | 11 |
| S.J.Huang 2022 | 2 | 2 | 2 | 2 | 1 | 1 | 1 | 2 | / | 13 |
| S.Huang 2022 | 2 | 2 | 2 | 2 | 2 | 1 | 1 | 0 | / | 12 |
| Z.Hong 2022 | 2 | 2 | 2 | 2 | 1 | 1 | 1 | 0 | 8 | 19 |
| Z.N.Hong 2022 | 2 | 2 | 2 | 2 | 1 | 1 | 1 | 0 | 8 | 19 |
| W.He 2022 | 2 | 2 | 2 | 2 | 2 | 1 | 1 | 0 | / | 12 |
| J.Guo 2022 | 2 | 1 | 2 | 2 | 1 | 1 | 1 | 0 | / | 10 |
| Y.M.Gu 2022 | 2 | 1 | 2 | 2 | 1 | 1 | 1 | 0 | / | 10 |
| T.Gong 2022 | 2 | 1 | 2 | 2 | 1 | 1 | 1 | 0 | / | 10 |
| L.Gao 2022 | 2 | 2 | 2 | 2 | 1 | 1 | 1 | 0 | / | 11 |
| J.Feng 2022 | 2 | 2 | 2 | 2 | 1 | 1 | 1 | 1 | / | 12 |
| H.Duan 2022 | 2 | 2 | 2 | 2 | 2 | 2 | 2 | 0 | / | 14 |
| Y.Dong 2022 | 2 | 1 | 2 | 2 | 1 | 1 | 1 | 0 | / | 10 |
| J.Cheng 2022 | 2 | 2 | 2 | 2 | 2 | 1 | 1 | 0 | 8 | 20 |
| F.Chen 2022 | 2 | 1 | 2 | 2 | 1 | 2 | 1 | 0 | / | 11 |
| L.Zhao 2021 | 2 | 1 | 2 | 2 | 1 | 1 | 2 | 0 | / | 11 |
| Z.Zhang 2021 | 2 | 1 | 2 | 2 | 1 | 2 | 1 | 0 | / | 11 |
| Z.Y.Zhang 2021 | 2 | 2 | 2 | 2 | 1 | 2 | 1 | 0 | / | 12 |
| X.Zhang 2021 | 2 | 1 | 2 | 2 | 1 | 1 | 1 | 0 | / | 10 |
| P.Yang 2021 | 2 | 1 | 2 | 2 | 1 | 1 | 1 | 2 | / | 12 |
| G.Z.Yang 2021 | 2 | 1 | 2 | 2 | 1 | 1 | 1 | 0 | / | 10 |
| S.Yamamoto 2021 | 2 | 1 | 2 | 2 | 1 | 1 | 1 | 0 | / | 10 |
| Z.Wu 2021 | 2 | 1 | 2 | 2 | 1 | 1 | 1 | 0 | / | 10 |
| P.Wu 2021 | 2 | 1 | 2 | 2 | 1 | 1 | 1 | 0 | / | 10 |
| F.Wang 2021 | 2 | 1 | 2 | 2 | 1 | 1 | 1 | 0 | / | 10 |
| T.V.D.Ende 2021 | 2 | 1 | 2 | 2 | 1 | 2 | 1 | 0 | / | 11 |
| S.Sihag 2021 | 2 | 2 | 2 | 2 | 1 | 2 | 1 | 1 | 8 | 21 |
| D.Shen 2021 | 2 | 1 | 2 | 2 | 1 | 1 | 1 | 0 | / | 10 |
| X.Shang 2021 | 2 | 1 | 2 | 2 | 1 | 1 | 1 | 0 | / | 10 |
| J.Ma 2021 | 2 | 1 | 2 | 2 | 1 | 1 | 1 | 0 | / | 10 |
| Lv 2021 | 2 | 1 | 2 | 2 | 1 | 1 | 1 | 0 | / | 10 |
| Hui.Lv 2021 | 2 | 1 | 2 | 2 | 1 | 1 | 1 | 0 | / | 10 |
| H.L.Lv 2021 | 2 | 1 | 2 | 2 | 1 | 1 | 1 | 0 | / | 10 |
| D.Liu 2021 | 2 | 1 | 2 | 2 | 1 | 1 | 1 | 0 | / | 10 |
| C.Li 2021 | 2 | 2 | 2 | 2 | 2 | 2 | 2 | 0 | / | 14 |
| G.Y.Ku 2021 | 2 | 1 | 2 | 2 | 1 | 1 | 1 | 0 | / | 10 |
| Hong 2021 | 2 | 1 | 2 | 2 | 1 | 1 | 1 | 0 | / | 10 |
| H.T.Duan 2021 | 2 | 1 | 2 | 2 | 1 | 1 | 1 | 0 | / | 10 |
| C.Cheng 2021 | 2 | 1 | 2 | 2 | 1 | 1 | 1 | 0 | / | 10 |
| A.Athauda 2021 | 2 | 1 | 2 | 2 | 1 | 1 | 1 | 0 | / | 10 |
| G.Zhang 2020 | 2 | 1 | 2 | 2 | 1 | 2 | 1 | 0 | / | 11 |
| W.Qi 2020 | 2 | 1 | 2 | 2 | 1 | 1 | 1 | 0 | / | 10 |
| S.Y.Park 2020 | 2 | 2 | 2 | 2 | 2 | 1 | 1 | 0 | 8 | 20 |
| K.Li 2020 | 2 | 1 | 2 | 2 | 1 | 2 | 1 | 0 | / | 11 |
| H.Li 2020 | 2 | 1 | 2 | 2 | 1 | 1 | 1 | 0 | / | 10 |
| Y.Gu 2020 | 2 | 1 | 2 | 2 | 1 | 1 | 1 | 0 | / | 10 |
| S.Lee 2019 | 2 | 1 | 2 | 2 | 1 | 2 | 1 | 1 | / | 12 |

The items are scored 0 (not reported), 1 (reported but inadequate) or 2 (reported and adequate). The global ideal score being 16 for non-comparative studies and 24 for comparative studies. For non-comparative studies, an overall score > 12 = high; 8–12 = intermediate; < 8 = low. For comparative studies, > 18 = high; 12–18 = intermediate; < 12 = low.

**Methodological items for non-randomized studies**

Q1. A clearly stated aim.

Q2. Inclusion of consecutive patients.

Q3. Prospective collection of data.

Q4. Endpoints appropriate to the aim of the study.

Q5. Unbiased assessment of the study endpoint.

Q6. Follow-up period appropriate to the aim of the study.

Q7. Loss to follow up less than 5%.

Q8. Prospective calculation of the study size.

**Additional criteria in the case of comparative study**

Q9. An adequate control group.

Q10. Contemporary groups.

Q11. Baseline equivalence of groups.

Q12. Adequate statistical analyses.

**Supplementary Table 10. Cochrane Risk of Bias for Randomized Controlled Trials**

| **Study** | **Bias arising from the randomization process** | **Bias due to deviations from intended interventions** | **Bias due to missing outcome data** | **Bias in measurement of the outcome** | **Bias in selection of the reported result** | **Overall bias** |
| --- | --- | --- | --- | --- | --- | --- |
| Y.Li 2022 | *+* | *+* | *+* | *+* | *+* | *+* |
| W.Xing 2021 | *+* | *+* | *+* | *+* | *+* | *+* |
| Y.Xiao 2021 | *+* | *+* | *+* | *+* | *+* | *+* |
| M.A.Shah 2021 | *+* | *+* | *+* | *+* | *?* | *?* |
| B.Huang 2021 | *+* | *+* | *?* | *+* | *?* | *?* |

*+ = low risk of bias, ? =some concerns, - = high risk of bias.*

**Overall risk of bias judgment Criteria**

Low risk of bias: The study is judged to be at low risk of bias for all domains for this result.

Some concerns: The study is judged to raise some concerns in at least one domain for this result, but not to be at high risk of bias for any domain.

High risk of bias: The study is judged to be at high risk of bias in at least one domain for this result, or the study is judged to have some concerns for multiple domains in a way that substantially lowers confidence in the result.

**Supplementary Table 11. PRISMA 2020 Checklist**

|  | **Item #** | **Checklist item** | **Location where item is reported** |
| --- | --- | --- | --- |
| **TITLE** | | |  |
| Title | 1 | Identify the report as a systematic review. | Title/page 1 |
| **ABSTRACT** | | |  |
| Abstract | 2 | See the PRISMA 2020 for Abstracts checklist. | Abstract/page 3-4 |
| **INTRODUCTION** | | |  |
| Rationale | 3 | Describe the rationale for the review in the context of existing knowledge. | Introduction/page 5 |
| Objectives | 4 | Provide an explicit statement of the objective(s) or question(s) the review addresses. | Introduction/page 5-6 |
| **METHODS** | | |  |
| Eligibility criteria | 5 | Specify the inclusion and exclusion criteria for the review and how studies were grouped for the syntheses. | Methods/page 7, Supplementary table 2-3/page 7-8 |
| Information sources | 6 | Specify all databases, registers, websites, organisations, reference lists and other sources searched or consulted to identify studies. Specify the date when each source was last searched or consulted. | Methods/page 7 |
| Search strategy | 7 | Present the full search strategies for all databases, registers and websites, including any filters and limits used. | Supplementary table 1/page 3-6 |
| Selection process | 8 | Specify the methods used to decide whether a study met the inclusion criteria of the review, including how many reviewers screened each record and each report retrieved, whether they worked independently, and if applicable, details of automation tools used in the process. | Methods/page 7-8 |
| Data collection process | 9 | Specify the methods used to collect data from reports, including how many reviewers collected data from each report, whether they worked independently, any processes for obtaining or confirming data from study investigators, and if applicable, details of automation tools used in the process. | Methods/page 7-8 |
| Data items | 10a | List and define all outcomes for which data were sought. Specify whether all results that were compatible with each outcome domain in each study were sought (e.g. for all measures, time points, analyses), and if not, the methods used to decide which results to collect. | Methods/page 8-9 |
|  | 10b | List and define all other variables for which data were sought (e.g. participant and intervention characteristics, funding sources). Describe any assumptions made about any missing or unclear information. | Methods/page 7-9 |
| Study risk of bias assessment | 11 | Specify the methods used to assess risk of bias in the included studies, including details of the tool(s) used, how many reviewers assessed each study and whether they worked independently, and if applicable, details of automation tools used in the process. | Methods/page 8-10, Supplementary table 9-10/page 29-33 |
| Effect measures | 12 | Specify for each outcome the effect measure(s) (e.g. risk ratio, mean difference) used in the synthesis or presentation of results. | Methods/page 9, Supplementary table 4/page 9 |
| Synthesis methods | 13a | Describe the processes used to decide which studies were eligible for each synthesis (e.g. tabulating the study intervention characteristics and comparing against the planned groups for each synthesis (item #5)). | Methods/page 7, Supplementary table 2-3/page 7-8 |
|  | 13b | Describe any methods required to prepare the data for presentation or synthesis, such as handling of missing summary statistics, or data conversions. | Methods/page 9, Supplementary table 4/page 9 |
|  | 13c | Describe any methods used to tabulate or visually display results of individual studies and syntheses. | Methods/page 9-10 |
|  | 13d | Describe any methods used to synthesize results and provide a rationale for the choice(s). If meta-analysis was performed, describe the model(s), method(s) to identify the presence and extent of statistical heterogeneity, and software package(s) used. | Methods/page 9-10 |
|  | 13e | Describe any methods used to explore possible causes of heterogeneity among study results (e.g. subgroup analysis, meta-regression). | Methods/page 10 |
|  | 13f | Describe any sensitivity analyses conducted to assess robustness of the synthesized results. | Methods/page 10 |
| Reporting bias assessment | 14 | Describe any methods used to assess risk of bias due to missing results in a synthesis (arising from reporting biases). | Methods/page 8-9 |
| Certainty assessment | 15 | Describe any methods used to assess certainty (or confidence) in the body of evidence for an outcome. | Methods/page 8-10 |
| **RESULTS** | | |  |
| Study selection | 16a | Describe the results of the search and selection process, from the number of records identified in the search to the number of studies included in the review, ideally using a flow diagram. | Results/page 11, Figure 1 |
|  | 16b | Cite studies that might appear to meet the inclusion criteria, but which were excluded, and explain why they were excluded. | Figure 1 |
| Study characteristics | 17 | Cite each included study and present its characteristics. | Results/page 11, Table 1, Supplementary table 5-8/page 10-28 |
| Risk of bias in studies | 18 | Present assessments of risk of bias for each included study. | Results/page 14, Supplementary table 9-10/ page 29-33 |
| Results of individual studies | 19 | For all outcomes, present, for each study: (a) summary statistics for each group (where appropriate) and (b) an effect estimate and its precision (e.g. confidence/credible interval), ideally using structured tables or plots. | Results/page 11 |
| Results of syntheses | 20a | For each synthesis, briefly summarise the characteristics and risk of bias among contributing studies. | Figure 2-6, Supplementary figure 1-4/page 48-51 |
|  | 20b | Present results of all statistical syntheses conducted. If meta-analysis was done, present for each the summary estimate and its precision (e.g. confidence/credible interval) and measures of statistical heterogeneity. If comparing groups, describe the direction of the effect. | Results/page 11-14 |
|  | 20c | Present results of all investigations of possible causes of heterogeneity among study results. | Results/page 14 |
|  | 20d | Present results of all sensitivity analyses conducted to assess the robustness of the synthesized results. | Results/page 14, Supplementary figure 11/page 64 |
| Reporting biases | 21 | Present assessments of risk of bias due to missing results (arising from reporting biases) for each synthesis assessed. | Results/page 14, Supplementary figure 12/page 65 |
| Certainty of evidence | 22 | Present assessments of certainty (or confidence) in the body of evidence for each outcome assessed. | Results/page 13-14, Supplementary figure 5-10/page 52-63 |
| **DISCUSSION** | | |  |
| Discussion | 23a | Provide a general interpretation of the results in the context of other evidence. | Discussion/page 16-20 |
|  | 23b | Discuss any limitations of the evidence included in the review. | Discussion/page 19-20 |
|  | 23c | Discuss any limitations of the review processes used. | Discussion/page 19-20 |
|  | 23d | Discuss implications of the results for practice, policy, and future research. | Conclusion/page 21 |
| **OTHER INFORMATION** | | |  |
| Registration and protocol | 24a | Provide registration information for the review, including register name and registration number, or state that the review was not registered. | Methods/page 7 |
|  | 24b | Indicate where the review protocol can be accessed, or state that a protocol was not prepared. | Methods/page 7 |
|  | 24c | Describe and explain any amendments to information provided at registration or in the protocol. | NA |
| Support | 25 | Describe sources of financial or non-financial support for the review, and the role of the funders or sponsors in the review. | Declarations/page 22 |
| Competing interests | 26 | Declare any competing interests of review authors. | Declarations/page 22 |
| Availability of data, code and other materials | 27 | Report which of the following are publicly available and where they can be found: template data collection forms; data extracted from included studies; data used for all analyses; analytic code; any other materials used in the review. | Methods/page 7-10, References/page 23-32, Supplementary table 5-13/page 10-47 |

*From:* Page MJ, McKenzie JE, Bossuyt PM, Boutron I, Hoffmann TC, Mulrow CD, et al. The PRISMA 2020 statement: an updated guideline for reporting systematic reviews. BMJ 2021;372:n71. doi: 10.1136/bmj.n71

For more information, visit: <http://www.prisma-statement.org/>

**Supplementary Table 12. AMSTAR-2 Checklist**

AMSTAR 2: a critical appraisal tool for systematic reviews that include randomised or non- randomised studies of healthcare interventions, or both

**1.** **Did** **the** **research** **questions** **and** **inclusion** **criteria** **for** **the** **review** **include** **the** **components** **of** **PICO?**

Optional (recommended)

Yes No

For Yes:

 Population

 Timeframe for follow-up

 Intervention

 Comparator group

 Outcome

**2.** **Did** **the** **report** **of** **the** **review** **contain** **an** **explicit** **statement** **that** **the** **review** **methods** **were** **established** **prior** **to** **the** **conduct** **of** **the** **review** **and** **did** **the** **report** **justify** **any** **significant** **deviations** **from** **the** **protocol?**

For Partial Yes:

For Yes:

As for partial yes, plus the protocol should be registered and should also have specified:

The authors state that they had a written

protocol or guide that included ALL the

following:

 review question(s)

Yes	Partial Yes No

 a plan for investigating causes

of heterogeneity

 justification for any deviations

from the protocol

a meta-analysis/synthesis plan, if appropriate, *and*

 a search strategy

 inclusion/exclusion criteria

 a risk of bias assessment

**3.** **Did** **the** **review** **authors** **explain** **their** **selection** **of** **the** **study** **designs** **for** **inclusion** **in** **the** **review?**

For Yes, the review should satisfy ONE of the following:

Yes No

 *Explanationfor* including only RCTs

 OR *Explanationfor* including only NRSI

 OR *Explanationfor* including both RCTs and NRSI

**4.** **Did** **the** **review** **authors** **use** **a** **comprehensive** **literature** **search** **strategy?**

For Yes, should also have (all the

For Partial Yes (all the following):

following):

 searched the reference lists /

Yes	Partial Yes No

 searched at least 2 databases

(relevant to research question)

 provided key word and/or

search strategy

 justified publication restrictions

(e.g. language)

bibliographies of included

studies

 searched trial/study registries

 included/consulted content

experts in the field

 where relevant, searched for

grey literature

 conducted search within 24

months of completion of the

review

**5.** **Did** **the** **review** **authors** **perform** **study** **selection** **in** **duplicate?**

For Yes, either ONE of the following:

 at least two reviewers independently agreed on selection of eligible studies

Yes No

and achieved consensus on which studies to include

 OR two reviewers selected a sample of eligible studies and achieved good

agreement (at least 80 percent), with the remainder selected by one

reviewer.

AMSTAR 2: a critical appraisal tool for systematic reviews that include randomised or non- randomised studies of healthcare interventions, or both

| **6.** **Did** **the** **review** **authors** **perform** **data** **extraction** **in** **duplicate?** For Yes, either ONE of the following:  at least two reviewers achieved consensus on which data to extract from   Yes No included studies  OR two reviewers extracted data from a sample of eligible studies and achieved good agreement (at least 80 percent), with the remainder extracted by one reviewer. **7.** **Did** **the** **review** **authors** **provide** **a** **list** **of** **excluded** **studies** **and** **justify** **the** **exclusions?** For Yes, must also have: Yes Partial Yes No  provided a list of all potentially relevant studies that were read in full-text form but excluded from the review For Partial Yes:     Justified the exclusion from the review of each potentially relevant study **8.** **Did** **the** **review** **authors** **describe** **the** **included** **studies** **in** **adequate** **detail?** For Yes, should also have ALL the For Partial Yes (ALL the following):  described populations  described interventions  described comparators  described outcomes  described research designs following:  described population in detail Yes Partial Yes No     described intervention in detail (including doses where relevant)  described comparator in detail (including doses where relevant)  described study’s setting  timeframe for follow-up **9.** **Did** **the** **review** **authors** **use** **a** **satisfactory** **technique** **for** **assessing** **the** **risk** **of** **bias** **(RoB)** **in** **individual** **studies** **that** **were** **included** **in** **the** **review?** **RCTs** For Partial Yes, must have assessed RoB For Yes, must also have assessed RoB from:  allocation sequence that was  Yes not truly random, *and*  Partial Yes  selection of the reported result  No from among multiple  Includes only measurements or analyses of a NRSI specified outcome from  unconcealed allocation, *and*  lack of blinding of patients and assessors when assessing outcomes (unnecessary for objective outcomes such as all- cause mortality); **NRSI** For Partial Yes, must have assessed For Yes, must also have assessed RoB:  methods used to ascertain  Yes exposures and outcomes, *and*  Partial Yes  selection of the reported result  No from among multiple  Includes only measurements or analyses of a RCTs specified outcome RoB:  from confounding, *and*  from selection bias **10.** **Did** **the** **review** **authors** **report** **on** **the** **sources** **of** **funding** **for** **the** **studies** **included** **in** **the** **review?** | | | |
| --- | --- | --- | --- |
|  | For Yes   Must have reported on the sources of funding for individual studies included  in the review. Note: Reporting that the reviewers looked for this information but it was not reported by study authors also qualifies |   | Yes  No |

AMSTAR 2: a critical appraisal tool for systematic reviews that include randomised or non- randomised studies of healthcare interventions, or both

**11.** **If** **meta-analysis** **was** **performed** **did** **the** **review** **authors** **use** **appropriate** **methods** **for** **statistical**

**combination** **of** **results?**

**RCTs**

For Yes:

 The authors justified combining the data in a meta-analysis

 Yes

 No No meta-analysis

conducted

 AND they used an appropriate weighted technique to combine

study results and adjusted for heterogeneity if present.

 AND investigated the causes of any heterogeneity

**For** **NRSI**

For Yes:

 The authors justified combining the data in a meta-analysis

 Yes

 No No meta-analysis

conducted

 AND they used an appropriate weighted technique to combine

study results, adjusting for heterogeneity if present

 AND they statistically combined effect estimates from NRSI that

were adjusted for confounding, rather than combining raw data,

or justified combining raw data when adjusted effect estimates

were not available

 AND they reported separate summary estimates for RCTs and

NRSI separately when both were included in the review

**12.** **If** **meta-analysis** **was** **performed,** **did** **the** **review** **authors** **assess** **the** **potential** **impact** **of** **RoB** **in**

**individual** **studies** **on** **the** **results** **of** **the** **meta-analysis** **or** **other** **evidence** **synthesis?**

For Yes:

 included only low risk of bias RCTs

 Yes

 No No meta-analysis

conducted

 OR, if the pooled estimate was based on RCTs and/or NRSI at variable

RoB, the authors performed analyses to investigate possible impact of

RoB on summary estimates of effect.

**13.** **Did** **the** **review** **authors** **account** **for** **RoB** **in** **individual** **studies** **when** **interpreting/** **discussing** **the** **results** **of** **the** **review?**

For Yes:

 included only low risk of bias RCTs

Yes No

 OR, if RCTs with moderate or high RoB, or NRSI were included the

review provided a discussion of the likely impact of RoB on the results

**14.** **Did** **the** **review** **authors** **provide** **a** **satisfactory** **explanation** **for,** **and** **discussion** **of,** **any** **heterogeneity** **observed** **in** **the** **results** **of** **the** **review?**

For Yes:

There was no significant heterogeneity in the results

OR if heterogeneity was present the authors performed an investigation of

Yes No

sources of any heterogeneity in the results and discussed the impact of this

on the results of the review

**15.** **If** **they** **performed** **quantitative** **synthesis** **did** **the** **review** **authors** **carry** **out** **an** **adequate** **investigation** **of** **publication** **bias** **(small** **study** **bias)** **and** **discuss** **its** **likely** **impact** **on** **the** **results** **of** **the** **review?**

For Yes:

performed graphical or statistical tests for publication bias and discussed

Yes

No No meta-analysis

conducted

the likelihood and magnitude of impact of publication bias

AMSTAR 2: a critical appraisal tool for systematic reviews that include randomised or non- randomised studies of healthcare interventions, or both

| **16.** **Did** **the** **review** **authors** **report** **any** **potential** **sources** **of** **conflict** **of** **interest,** **including** **any** **funding** **they** **received** **for** **conducting** **the** **review?** | |
| --- | --- |
| For Yes:  The authors reported no competing interests OR Yes  The authors described their funding sources and how they managed No  potential conflicts of interest |  |

**To** **cite** **this** **tool:** Shea BJ, Reeves BC, Wells G, Thuku M, Hamel C, Moran J, Moher D, Tugwell P, Welch V, Kristjansson E, Henry DA. AMSTAR 2: a critical appraisal tool for systematic reviews that include randomised or non-randomised studies of healthcare interventions, or both. BMJ. 2017 Sep 21;358:j4008.

**Supplementary Table 13. References**

1.	Siegel RL, Miller KD, Fuchs HE, Jemal A. Cancer statistics, 2022. CA Cancer J Clin. 2022;72(1):7-33.

2.	Paul S, Altorki N. Outcomes in the management of esophageal cancer. J Surg Oncol. 2014;110(5):599-610.

3.	P. van Hagen MCCMH, J.J.B. van Lanschot, E.W. Steyerberg, , M.I. van Berge Henegouwen BPLW, D.J. Richel, , G.A.P. Nieuwenhuijzen GAPH, J.J. Bonenkamp, M.A. Cuesta, , R.J.B. Blaisse ORCB, F.J.W. ten Kate, G.-J. Creemers, C.J.A. Punt, , J.T.M. Plukker HMWV, E.J. Spillenaar Bilgen, H. van Dekken, , M.J.C. van der Sangen TR, K. Biermann, J.C. Beukema, , et al. Preoperative chemoradiotherapy for esophageal or junctional cancer. N Engl J Med. 2012.

4.	Ajani JA, D'Amico TA, Bentrem DJ, Chao J, Corvera C, Das P, et al. Esophageal and Esophagogastric Junction Cancers, Version 2.2019, NCCN Clinical Practice Guidelines in Oncology. J Natl Compr Canc Netw. 2019;17(7):855-83.

5.	Y. Baba NY, K. Kinoshita, M. Iwatsuki, Y.-I. Yamashita, A. Chikamoto, M. Watanabe, H. Baba. Clinical and Prognostic Features of Patients With Esophageal Cancer and Multiple Primary Cancers: A Retrospective Single-institution Study. Ann Surg. 2018;267(3):478-83.

6.	T.X. Huang LF. The immune landscape of esophageal cancer. Cancer Commun (Lond). 2019;39(1):79.

7.	Y.Y. Janjigian JB, E. Calvo, J.W. Kim, P.A. Ascierto, P. Sharma, P.A. Ott, K. Peltola, D. Jaeger, J. Evans, F. de Braud, I. Chau, C.T. Harbison, C. Dorange, M. Tschaika, D.T. Le. CheckMate-032 Study: Efficacy and Safety of Nivolumab and Nivolumab Plus Ipilimumab in Patients With Metastatic Esophagogastric Cancer. J Clin Oncol. 2018;36(28):2836-44.

8.	T. Kojima MAS, K. Muro, E. Francois, A. Adenis, C.-H. Hsu, T. Doi, T. Moriwaki, S.-B. Kim, S.-H. Lee, J. Bennouna, K. Kato, L. Shen, P. Enzinger, S.-K. Qin, P. Ferreira, J. Chen, G. Girotto, C. de la Fouchardiere, H. Senellart, R. Al-Rajabi, F. Lordick, R. Wang, S. Suryawanshi, P. Bhagia, S.P. Kang, J.-P. Metges. Randomized Phase III KEYNOTE-181 Study of Pembrolizumab Versus Chemotherapy in Advanced Esophageal Cancer. J Clin Oncol. 2020;38(35):4138-48.

9.	A. Ribas JDW. Cancer immunotherapy using checkpoint blockade. Science. 2018;359(6382):1350-5.

10.	Li Y, Zhou A, Liu S, He M, Chen KN, Tian Z, et al. 1207P Neoadjuvant PD-L1 inhibitor (socazolimab) plus chemotherapy in patients with locally advanced esophageal squamous cell carcinoma (ESCC): A multicenter, randomized, double-blind phase II study. Annals of Oncology. 2022;33.

11.	Guo J, Qiao C, Lu J, Yang S, Zhang B. Neoadjuvant sintilimab combined with chemotherapy in patients with resectable esophageal squamous cell carcinoma (ESCC): Preliminary results from a phase II study. Journal of Clinical Oncology. 2022;40(16):E16008-E.

12.	Li C, Zhao S, Zheng Y, Han Y, Chen X, Cheng Z, et al. Preoperative pembrolizumab combined with chemoradiotherapy for oesophageal squamous cell carcinoma (PALACE-1). Eur J Cancer. 2021;144:232-41.

13.	Kelly RJ, Zaidi AH, Van Liere Canzoniero J, Feliciano JL, Hales RK, Voong KR, et al. Multicenter phase II study of neoadjuvant nivolumab or nivolumab plus relatlimab (antiLAG3 antibody) plus chemoradiotherapy in stage II/III esophageal/gastroesophageal junction (E/GEJ) carcinoma. Journal of Clinical Oncology. 2022;40(4 SUPPL).

14.	Page MJ, McKenzie JE, Bossuyt PM, Boutron I, Hoffmann TC, Mulrow CD, et al. The PRISMA 2020 statement: An updated guideline for reporting systematic reviews. J Clin Epidemiol. 2021;134:178-89.

15.	KAREM SLIM, EMILE NINI, DAMIEN FORESTIER, FABRICE KWIATKOWSKI, YVES PANIS, CHIPPONI. J. METHODOLOGICAL INDEX FOR NON-RANDOMIZED STUDIES (MINORS): DEVELOPMENT AND VALIDATION OF A NEW INSTRUMENT. ANZ J Surg. 2003;73(9):712-6.

16.	Malgie J, Schoones JW, Pijls BG. Decreased Mortality in Coronavirus Disease 2019 Patients Treated With Tocilizumab: A Rapid Systematic Review and Meta-analysis of Observational Studies. Clin Infect Dis. 2021;72(11):e742-e9.

17.	Higgins JP, Altman DG, Gotzsche PC, Juni P, Moher D, Oxman AD, et al. The Cochrane Collaboration's tool for assessing risk of bias in randomised trials. BMJ. 2011;343:d5928.

18.	Sterne JAC, Savovic J, Page MJ, Elbers RG, Blencowe NS, Boutron I, et al. RoB 2: a revised tool for assessing risk of bias in randomised trials. BMJ. 2019;366:l4898.

19.	Mazumdar CBBaM. Operating Characteristics of a Rank Correlation Test for Publication Bias. Biometrics. 1994;50(4):1088-101.

20.	Zhu M, Chen C, Foster NR, Hartley C, Mounajjed T, Salomao MA, et al. Pembrolizumab in Combination with Neoadjuvant Chemoradiotherapy for Patients with Resectable Adenocarcinoma of the Gastroesophageal Junction. Clin Cancer Res. 2022;28(14):3021-31.

21.	Zhou YH, Li JY, Yan JX, Guo P, He WW, Liu Y. Alleviation of neoadjuvant immunochemotherapy for esophageal squamous cell carcinoma and its relationship with expression and changes of PD-L1. Neoplasma. 2022;69(4):785-93.

22.	Zhang Z, Ye J, Li H, Gu D, Du M, Ai D, et al. Neoadjuvant sintilimab and chemotherapy in patients with resectable esophageal squamous cell carcinoma: A prospective, single-arm, phase 2 trial. Front Immunol. 2022;13:1031171.

23.	Zhang Y, Shen G, Xu R, Huang G, Yang S, Zheng Q, et al. Real-world effectiveness and safety of camrelizumab-based neoadjuvant therapy in resectable esophageal cancer: Initial results of a prospective multicenter observational study. Journal of Clinical Oncology. 2022;40(4_suppl):250-.

24.	Zhang X, Gari A, Li M, Chen J, Qu C, Zhang L, et al. Combining serum inflammation indexes at baseline and post treatment could predict pathological efficacy to anti‑PD‑1 combined with neoadjuvant chemotherapy in esophageal squamous cell carcinoma. J Transl Med. 2022;20(1):61.

25.	Zhang G, Yuan J, Pan C, Xu Q, Cui X, Zhang J, et al. Abstract CT544: Multi-omics analysis uncovers prognostic biomarkers and tumor ecosystem dynamics during toripalimab combined with nab-paclitaxel and s-1 as neoadjuvant therapy for esophageal squamous carcinoma: A single-center, open-label, single-arm phase 2 trial. Cancer Research. 2022;82(12_Supplement):CT544-CT.

26.	Yu Y, Wang W, Qin Z, Li H, Liu Q, Ma H, et al. A clinical nomogram for predicting tumor regression grade in esophageal squamous-cell carcinoma treated with immune neoadjuvant immunotherapy. Ann Transl Med. 2022;10(2):102.

27.	Yin GQ, Li ZL, Li D. The Safety and Efficacy of Neoadjuvant Camrelizumab Plus Chemotherapy in Patients with Locally Advanced Esophageal Squamous Cell Carcinoma: A Retrospective Study. Cancer Manag Res. 2022;14:2133-41.

28.	Yang Y, Tan L, Hu J, Li Y, Mao Y, Tian Z, et al. Safety and efficacy of neoadjuvant treatment with immune checkpoint inhibitors in esophageal cancer: real-world multicenter retrospective study in China. Dis Esophagus. 2022.

29.	Yang W, Xing X, Yeung SJ, Wang S, Chen W, Bao Y, et al. Neoadjuvant programmed cell death 1 blockade combined with chemotherapy for resectable esophageal squamous cell carcinoma. J Immunother Cancer. 2022;10(1).

30.	Yang G, Sun X, Yang H, Luo G, Zheng Y, Huang M, et al. 1256P Three courses of neoadjuvant camrelizumab combined with chemotherapy in locally advanced esophageal squamous cell carcinoma (ESCC): A prospective phase II clinical trial. Annals of Oncology. 2022;33.

31.	Yan X, Duan H, Ni Y, Zhou Y, Wang X, Qi H, et al. Tislelizumab combined with chemotherapy as neoadjuvant therapy for surgically resectable esophageal cancer: A prospective, single-arm, phase II study (TD-NICE). Int J Surg. 2022;103:106680.

32.	Xu X, Sun Z, Zhang Y, Shen L, Liu Q, Zhang C, et al. Neoadjuvant chemoradiotherapy combined with perioperative toripalimab in locally advanced esophageal cancer. Journal of Clinical Oncology. 2022;40(16):E16065-E.

33.	Xu W, Jiang Y, Wang C, Wu J, Li J, Hu Y, et al. The efficacy and safety of neoadjuvant camrelizumab and chemotherapy for locally advanced thoracic esophageal squamous cell carcinoma. Journal of Clinical Oncology. 2022;40(4_suppl):278-.

34.	Xu L, Wei XF, Li CJ, Yang ZY, Yu YK, Li HM, et al. Pathologic responses and surgical outcomes after neoadjuvant immunochemotherapy versus neoadjuvant chemoradiotherapy in patients with locally advanced esophageal squamous cell carcinoma. Front Immunol. 2022;13:1052542.

35.	Xu L, Qi Y, Jiang Y, Ji Y, Zhao Q, Wu J, et al. Crosstalk between the gut microbiome and clinical response in locally advanced thoracic esophageal squamous cell carcinoma during neoadjuvant camrelizumab and chemotherapy. Ann Transl Med. 2022;10(6):325.

36.	Xiao X, Yang YS, Zeng XX, Shang QX, Luan SY, Zhou JF, et al. The comparisons of neoadjuvant chemoimmunotherapy versus chemoradiotherapy for oesophageal squamous cancer. Eur J Cardiothorac Surg. 2022;62(1).

37.	Xia P, Li P, Wu S, Wang Y, Ye P, Zhang C, et al. Evaluation of the safety and effectiveness of neoadjuvant combined chemoimmunotherapy in the treatment of locally advanced esophageal squamous cell carcinoma: a retrospective single-arm cohort study. Ann Transl Med. 2022;10(18):991.

38.	Wang X, Yang W, Zhou Q, Luo H, Chen W, Yeung SJ, et al. The role of (18)F-FDG PET/CT in predicting the pathological response to neoadjuvant PD-1 blockade in combination with chemotherapy for resectable esophageal squamous cell carcinoma. Eur J Nucl Med Mol Imaging. 2022;49(12):4241-51.

39.	Wang W, Li L. Neoadjuvant pembrolizumab plus chemotherapy for resectable locally advanced esophageal squamous cell carcinoma (ESCC): Interim results. Journal of Clinical Oncology. 2022;40(16):E16011-E.

40.	Wang R, Zhang G, Zhu Q, Ma T, Weng C, Zhang D, et al. 1234P Neoadjuvant camrelizumab plus docetaxel and carboplatin in locally advanced esophageal squamous cell carcinoma (ESCC): A prospective study. Annals of Oncology. 2022;33.

41.	Uboha NV, Eickhoff JC, Maloney JD, McCarthy D, De Camp M, Deming DA, et al. Phase I/II trial of perioperative avelumab in combination with chemoradiation (CRT) in the treatment of stage II/III resectable esophageal and gastroesophageal junction (E/ GEJ) cancer. Journal of Clinical Oncology. 2022;40(16).

42.	Qiao Y, Zhao C, Li X, Zhao J, Huang Q, Ding Z, et al. Efficacy and safety of camrelizumab in combination with neoadjuvant chemotherapy for ESCC and its impact on esophagectomy. Front Immunol. 2022;13:953229.

43.	Qi Y, Meng X, Shan Z, Fan Q, Wang F. CAMRELIZUMAB COMBINED WITH CHEMOTHERAPY OR APATINIB AS NEOADJUVANT THERAPY FOR LOCALLY ADVANCED ESOPHAGEAL SQUAMOUS CELL CARCINOMA: A PHASE 2 TRIAL. Diseases of the Esophagus. 2022;35:62.

44.	Matsuda S, Yamamoto S, Kato K, Daiko H, Kojima T, Hara H, et al. FRONTiER: A feasibility trial of nivolumab with neoadjuvant CF or DCF, FLOT therapy for locally advanced esophageal carcinoma (JCOG1804E)—Short-term results for cohorts C and D. Journal of Clinical Oncology. 2022;40(4_suppl):286-.

45.	Ma X, Zhao W, Li B, Yu Y, Ma Y, Thomas M, et al. Neoadjuvant Immune Checkpoint Inhibitors Plus Chemotherapy in Locally Advanced Esophageal Squamous Cell Carcinoma: Perioperative and Survival Outcomes. Front Oncol. 2022;12:810898.

46.	Lv H, Tian Y, Li J, Huang C, Sun B, Gai C, et al. Neoadjuvant Sintilimab Plus Chemotherapy in Resectable Locally Advanced Esophageal Squamous Cell Carcinoma. Front Oncol. 2022;12:864533.

47.	Liu J, Yang Y, Liu Z, Fu X, Cai X, Li H, et al. Multicenter, single-arm, phase II trial of camrelizumab and chemotherapy as neoadjuvant treatment for locally advanced esophageal squamous cell carcinoma. J Immunother Cancer. 2022;10(3).

48.	Liu J, Li J, Lin W, Shao D, Depypere L, Zhang Z, et al. Neoadjuvant camrelizumab plus chemotherapy for resectable, locally advanced esophageal squamous cell carcinoma (NIC-ESCC2019): A multicenter, phase 2 study. Int J Cancer. 2022;151(1):128-37.

49.	Li Z, Xu PF, Mao WM, Kuang YK, Fan HY, Zou B, et al. A study of neoadjuvant sintilimab combined with chemotherapy TP for locally advanced esophageal squamous cell carcinoma (ESCC). Journal of Clinical Oncology. 2022;40(16).

50.	Ko AH, Noel M, Chao J, Sohal D, Crow M, Oberstein PE, et al. 1229P A multicenter phase II study of sotigalimab (CD40 agonist) in combination with neoadjuvant chemoradiation for resectable esophageal and gastroesophageal junction (GEJ) cancers. Annals of Oncology. 2022;33.

51.	Jing SW, Zhai C, Zhang W, He M, Liu QY, Yao JF, et al. Comparison of neoadjuvant immunotherapy plus chemotherapy versus chemotherapy alone for patients with locally advanced esophageal squamous cell carcinoma: A propensity score matching. Front Immunol. 2022;13:970534.

52.	Jiang N, Jiang M, Zhu X, Ren B, Zhang J, Guo Z, et al. SCALE-1: Safety and efficacy of short course neoadjuvant chemo-radiotherapy plus toripalimab for locally advanced resectable squamous cell carcinoma of esophagus. Journal of Clinical Oncology. 2022;40(16).

53.	Jiang B, Yang X, Zhang J, Huang M. Abstract 5230: Neoadjuvant programmed cell death protein 1 inhibitors combined with chemotherapy in resectable esophageal squamous carcinoma: an open-label, single-arm study. Cancer Research. 2022;82(12_Supplement):5230-.

54.	Huang SJ, Tian D, Wang SC, Zeng RJ, Dong YJ, Hong LL, et al. Pathological Responses of the Primary Tumor and Locoregional Lymph Nodes After Neoadjuvant Immunochemotherapy in Esophageal Squamous Cell Cancer. World J Oncol. 2022;13(4):195-204.

55.	Huang S, Wu H, Cheng C, Zhou M, Xu E, Lin W, et al. Conversion Surgery Following Immunochemotherapy in Initially Unresectable Locally Advanced Esophageal Squamous Cell Carcinoma-A Real-World Multicenter Study (RICE-Retro). Front Immunol. 2022;13:935374.

56.	Hong ZN, Zhang Z, Chen Z, Weng K, Peng K, Lin J, et al. Safety and feasibility of esophagectomy following combined neoadjuvant immunotherapy and chemotherapy for locally advanced esophageal cancer: a propensity score matching. Esophagus. 2022;19(2):224-32.

57.	Hong ZN, Gao L, Weng K, Huang Z, Han W, Kang M. Safety and Feasibility of Esophagectomy Following Combined Immunotherapy and Chemotherapy for Locally Advanced Esophageal Squamous Cell Carcinoma: A Propensity Score Matching Analysis. Front Immunol. 2022;13:836338.

58.	He W, Leng X, Mao T, Luo X, Zhou L, Yan J, et al. Toripalimab Plus Paclitaxel and Carboplatin as Neoadjuvant Therapy in Locally Advanced Resectable Esophageal Squamous Cell Carcinoma. Oncologist. 2022;27(1):e18-e28.

59.	Gu YM, Shang QX, Zhang HL, Yang YS, Wang WP, Yuan Y, et al. Safety and Feasibility of Esophagectomy Following Neoadjuvant Immunotherapy Combined with Chemotherapy for Esophageal Squamous Cell Carcinoma. Front Surg. 2022;9:851745.

60.	Gong T, Song W, Lu J, Liu J, Fan B, Zhao J, et al. EFFICACY AND SAFETY OF NEOADJUVANT CHEMOTHERAPY AND IMMUNOTHERAPY IN LOCALLY RESECTABLE ADVANCED ESOPHAGEAL SQUAMOUS CELL CARCINOMA. Diseases of the Esophagus. 2022;35:108-9.

61.	Gao L, Lu J, Zhang P, Hong ZN, Kang M. Toripalimab combined with docetaxel and cisplatin neoadjuvant therapy for locally advanced esophageal squamous cell carcinoma: a single-center, single-arm clinical trial (ESONICT-2). J Gastrointest Oncol. 2022;13(2):478-87.

62.	Feng J, Wang L, Yang X, Chen Q, Cheng X. Pathologic Complete Response Prediction to Neoadjuvant Immunotherapy Combined with Chemotherapy in Resectable Locally Advanced Esophageal Squamous Cell Carcinoma: Real-World Evidence from Integrative Inflammatory and Nutritional Scores. J Inflamm Res. 2022;15:3783-96.

63.	Duan H, Shao C, Pan M, Liu H, Dong X, Zhang Y, et al. Neoadjuvant Pembrolizumab and Chemotherapy in Resectable Esophageal Cancer: An Open-Label, Single-Arm Study (PEN-ICE). Front Immunol. 2022;13:849984.

64.	Dong Y, Luo J, Liu S, Lin Y, Han H. 1225P Neoadjuvant therapy of camrelizumab combined with chemotherapy in patients (pts) with resectable esophageal squamous cell cancer (ESCC): Preliminary results of a preoperative phase exploratory clinical trial. Annals of Oncology. 2022;33.

65.	Cheng J, Guo M, Yang Y, Liu Y, Hu W, Shang Q, et al. Perioperative Outcomes of Minimally Invasive Esophagectomy After Neoadjuvant Immunotherapy for Patients With Locally Advanced Esophageal Squamous Cell Carcinoma. Front Immunol. 2022;13:848881.

66.	Chen F, Qiu L, Mu Y, Sun S, Yuan Y, Shang P, et al. Neoadjuvant chemoradiotherapy with camrelizumab in patients with locally advanced esophageal squamous cell carcinoma. Front Surg. 2022;9:893372.

67.	Zhao L, Xing W, Yang Y, Zhang Y, Ma B, Fu X, et al. The sequence of chemotherapy and anti-PD-1 antibody influence the efficacy of neoadjuvant immunochemotherapy in locally advanced esophageal squamous cell cancer: A phase II study. Journal of Clinical Oncology. 2021;39(15_suppl):4051-.

68.	Zhang Z, Ye J, Li H, Du M, Gu D, Zhang J, et al. 1378P A single-center, prospective, open-label, single-arm trial of sintilimab with paclitaxel and carboplatin as a neoadjuvant therapy for esophageal squamous carcinoma. Annals of Oncology. 2021;32:S1042-S3.

69.	Zhang Z, Hong ZN, Xie S, Lin W, Lin Y, Zhu J, et al. Neoadjuvant sintilimab plus chemotherapy for locally advanced esophageal squamous cell carcinoma: a single-arm, single-center, phase 2 trial (ESONICT-1). Ann Transl Med. 2021;9(21):1623.

70.	Zhang X, Yang G, Su X, Luo G, Cai P, Zheng Y, et al. Neoadjuvant programmed death1 blockade plus chemotherapy in locally advanced esophageal squamous cell carcinoma. Journal of Clinical Oncology. 2021;39(15 SUPPL).

71.	Yang P, Zhou X, Yang X, Wang Y, Sun T, Feng S, et al. Neoadjuvant camrelizumab plus chemotherapy in treating locally advanced esophageal squamous cell carcinoma patients: a pilot study. World J Surg Oncol. 2021;19(1):333.

72.	Yang G, Su X, Yang H, Luo G, Gao C, Zheng Y, et al. Neoadjuvant programmed death-1 blockade plus chemotherapy in locally advanced esophageal squamous cell carcinoma. Ann Transl Med. 2021;9(15):1254.

73.	Yamamoto S, Kato K, Daiko H, Kojima T, Hara H, Abe T, et al. FRONTiER: A feasibility trial of nivolumab with neoadjuvant CF or DCF therapy for locally advanced esophageal carcinoma (JCOG1804E)-The short-term results of cohort A and B. Journal of Clinical Oncology. 2021;39(3).

74.	Xing W, Zhao L, Zheng Y, Liu B, Liu X, Li T, et al. The Sequence of Chemotherapy and Toripalimab Might Influence the Efficacy of Neoadjuvant Chemoimmunotherapy in Locally Advanced Esophageal Squamous Cell Cancer-A Phase II Study. Front Immunol. 2021;12:772450.

75.	Xiao Y, Huo Q, Yang Y, Lu B, Wang X. Clinical trial of carrelizumab injection combined with operation in the treatment of patients with stage Ⅱ/Ⅲ esophageal squamous cell carcinoma. The Chinese Journal of Clinical Pharmacology. 2021;37(24):3323-5,33.

76.	Wu Z, Zheng Q, Chen H, Xiang J, Hu H, Li H, et al. Efficacy and safety of neoadjuvant chemotherapy and immunotherapy in locally resectable advanced esophageal squamous cell carcinoma. J Thorac Dis. 2021;13(6):3518-28.

77.	Wu P, Wang T, Chen B, Shi M, Zhou Y, Huang B, et al. Preoperative chemotherapy combined with PD-1 inhibitor in locally advanced operable or potentially resectable esophageal squamous cell carcinoma: A real world study. Journal of Clinical Oncology. 2021;39(15_suppl):e16010-e.

78.	Wang F, Qi Y, Meng X, Fan Q. Camrelizumab in combination with preoperative chemotherapy for locally advanced esophageal squamous cell carcinoma: A single-arm, open-label, phase II study. Journal of Clinical Oncology. 2021;39(15).

79.	van den Ende T, de Clercq NC, van Berge Henegouwen MI, Gisbertz SS, Geijsen ED, Verhoeven RHA, et al. Neoadjuvant Chemoradiotherapy Combined with Atezolizumab for Resectable Esophageal Adenocarcinoma: A Single-arm Phase II Feasibility Trial (PERFECT). Clin Cancer Res. 2021;27(12):3351-9.

80.	Sihag S, Ku GY, Tan KS, Nussenzweig S, Wu A, Janjigian YY, et al. Safety and feasibility of esophagectomy following combined immunotherapy and chemoradiotherapy for esophageal cancer. J Thorac Cardiovasc Surg. 2021;161(3):836-43 e1.

81.	Shen D, Chen Q, Wu J, Li J, Tao K, Jiang Y. The safety and efficacy of neoadjuvant PD-1 inhibitor with chemotherapy for locally advanced esophageal squamous cell carcinoma. J Gastrointest Oncol. 2021;12(1):1-10.

82.	Shang X, Zhang C, Zhao G, Zhang W, Liu L, Duan X, et al. LBA3 Safety and efficacy of pembrolizumab combined with paclitaxel and cisplatin as a neoadjuvant treatment for locally advanced resectable (stage III) esophageal squamous cell carcinoma (Keystone-001): Interim analysis of a prospective, single-arm, single-center, phase II trial. Annals of Oncology. 2021;32:S1428-S9.

83.	Shah MA, Almhanna K, Iqbal S, Thakkar P, Schneider BJ, Yantiss R, et al. Multicenter, randomized phase II study of neoadjuvant pembrolizumab plus chemotherapy and chemoradiotherapy in esophageal adenocarcinoma (EAC). Journal of Clinical Oncology. 2021;39(15_suppl):4005-.

84.	Ma J, Zhang J, Yang Y, Zheng D, Wang X, Liang H, et al. Camrelizumab combined with paclitaxel and nedaplatin as neoadjuvant therapy for locally advanced esophageal squamous cell carcinoma (ESPRIT): A phase II, single-arm, exploratory research. Annals of Oncology. 2021;32:S1400-S.

85.	Lv H, Tian Y, Li Z, Huang C, Xu Y, Tian Z. 557 Neoadjuvant Camrelizumab in combination with albumin paclitaxel and cisplatin for patients with locally advanced esophageal squamous cell carcinoma (ESCC). Journal for ImmunoTherapy of Cancer. 2021;9(Suppl 2):A587-A.

86.	Lv H, Tian Y, Huang C, Sun B, Gai C, Li Z, et al. 110P Neoadjuvant PD-1 blockade combined with chemotherapy for patients with resectable locally advanced esophageal squamous cell carcinoma (ESCC): A real world data analysis. Annals of Oncology. 2021;32.

87.	Lv H, Tian Y, Huang C, Li Z, Tian Z. Camrelizumab combined with albumin paclitaxel and cisplatin as neoadjuvant therapy for locally advanced esophageal squamous cell carcinoma (ESCC). Journal of Clinical Oncology. 2021;39(15).

88.	Liu D, Zhang Q, Zhu J, Qian T, Yin R, Fan Z, et al. Phase-II study of toripalimab combined with neoadjuvant chemotherapy for the treatment of resectable esophageal squamous cell carcinoma. Journal of Clinical Oncology. 2021;39(15).

89.	Ku GY, Wu AJ-C, Sihag S, Park BJ, Jones DR, Gu P, et al. Durvalumab (D) and PET-directed chemoradiation (CRT) after induction FOLFOX for esophageal adenocarcinoma. Journal of Clinical Oncology. 2021;39(3_suppl):226-.

90.	Huang B, Shi H, Gong X, Yu J, Xiao C, Zhou B, et al. Comparison of efficacy and safety between pembrolizumab combined with chemotherapy and simple chemotherapy in neoadjuvant therapy for esophageal squamous cell carcinoma. J Gastrointest Oncol. 2021;12(5):2013-21.

91.	Hong ZN, Weng K, Peng K, Chen Z, Lin J, Kang M. Neoadjuvant Immunotherapy Combined Chemotherapy Followed by Surgery Versus Surgery Alone for Locally Advanced Esophageal Squamous Cell Carcinoma: A Propensity Score-Matched Study. Front Oncol. 2021;11:797426.

92.	Duan H, Wang T, Luo Z, Wang X, Liu H, Tong L, et al. A multicenter single-arm trial of sintilimab in combination with chemotherapy for neoadjuvant treatment of resectable esophageal cancer (SIN-ICE study). Ann Transl Med. 2021;9(22):1700.

93.	Cheng C, Yang W, Chen W, Yeung S-CJ, Xing X, Wang X, et al. Neoadjuvant PD-1 blockade in combination with chemotherapy for patients with resectable esophageal squamous cell carcinoma. Journal of Clinical Oncology. 2021;39(3_suppl):220-.

94.	Athauda A, Starling N, Chau I, Cunningham D, Watkins DJ, Rao S, et al. Perioperative FLOT plus anti-PD-L1 avelumab (FLOT-A) in resectable oesophagogastric adenocarcinoma (OGA): Interim safety analysis results from the ICONIC trial. Journal of Clinical Oncology. 2021;39(3_suppl):201-.

95.	Zhang G, Hu Y, Yang B, Xu Q, Li J, Sun S, et al. 1058P A single-centre, prospective, open-label, single-arm trial of toripalimab with nab-paclitaxel and S-1 as a neoadjuvant therapy for esophageal squamous cell carcinoma (ESCC). Annals of Oncology. 2020;31.

96.	Qi WX, Zhao S, Li H, Chen J. Safety And Tolerability Of Neoadjuvant Chemoradiotherapy Combined With Pembrolizumab For Local Advanced, Resectable Esophageal Cancer: Preliminary Results Of A Prospective Phase IB Trial. International Journal of Radiation Oncology*Biology*Physics. 2020;108(3):e576-e7.

97.	Park SY, Hong MH, Kim HR, Lee CG, Cho JH, Cho BC, et al. The feasibility and safety of radical esophagectomy in patients receiving neoadjuvant chemoradiotherapy with pembrolizumab for esophageal squamous cell carcinoma. J Thorac Dis. 2020;12(11):6426-34.

98.	Li K, Yang X, Luo W, Ma Q, Wang Y, Xiong Y, et al. TORIPALIMAB PLUS NAB-PACLITAXEL AND CARBOPLATIN AS NEOADJUVANT THERAPY FOR PATIENTS WITH ESOPHAGEAL SQUAMOUS CELL CARCINOMA AT CLINICAL STAGE T2-T4/N0-N2/M0: A SINGLE-ARM, SINGLE-CENTER CLINICAL STUDY. Journal for Immunotherapy of Cancer. 2020;8:A253-A.

99.	Li H, Li C, Zheng Y, Zhao S, Chen X, Han Y, et al. Preoperative combination ofpembrolizumab withchemoradiation for patients withlocally advanced esophagealsquamous cell carcinoma:mid-termresults of NCT03604991. Diseases of the Esophagus. 2020;33(SUPPL 1).

100.	Gu Y, Chen X, Wang D, Ding M, Xue L, Zhen F, et al. 175P A study of neoadjuvant sintilimab combined with triplet chemotherapy of lipo-paclitaxel, cisplatin, and S-1 for resectable esophageal squamous cell carcinoma (ESCC). Annals of Oncology. 2020;31:S1307-S8.

101.	Lee S, Ahn BC, Park SY, Kim DJ, Lee CG, Cho J, et al. A phase II trial of preoperative chemoradiotherapy and pembrolizumab for locally advanced esophageal squamous cell carcinoma (ESCC). Annals of Oncology. 2019;30.

102.	Wang Z, Shao C, Wang Y, Duan H, Pan M, Zhao J, et al. Efficacy and safety of neoadjuvant immunotherapy in surgically resectable esophageal cancer: A systematic review and meta-analysis. Int J Surg. 2022;104:106767.

103.	Li F, Ding N, Zhao Y, Yuan L, Mao Y. The current optimal multimodality treatments for oesophageal squamous-cell carcinoma: A systematic review and meta-analysis. Int J Surg. 2018;60:88-100.

104.	Ge F, Huo Z, Cai X, Hu Q, Chen W, Lin G, et al. Evaluation of Clinical and Safety Outcomes of Neoadjuvant Immunotherapy Combined With Chemotherapy for Patients With Resectable Esophageal Cancer: A Systematic Review and Meta-analysis. JAMA Netw Open. 2022;5(11):e2239778.

105.	Liu J, Wang Y, Cao B, Zhang S, Cao F, Gao L, et al. A randomized, controlled, multicenter study of nab-paclitaxel plus cisplatin followed by surgery versus surgery alone for locally advanced esophageal squamous cell carcinoma (ESCC). Journal of Clinical Oncology. 2022;40(4_suppl):310-.

**Supplementary Figure 1.** Network Evidence Plot of (A) R0, (B) Pathological Complete Response (pCR), (C) Major Pathological Response (mPR), (D) Death within 30 Days after Surgery, (E) 1-year Overall Survival (OS) and (F) 1-year Disease Free Survival (DFS).

**Supplementary Figure 2.** Contribution Plot of (A) R0, (B) Pathological Complete Response (pCR), (C) Major Pathological Response (mPR), (D) Death within 30 Days after Surgery, (E) 1-year Overall Survival (OS) and (F) 1-year Disease Free Survival (DFS).

**Supplementary Figure 3.** Forest Plot of Network Meta-analysis. (A) R0, (B) Pathological Complete Response (pCR), (C) Major Pathological Response (mPR), (D) Death within 30 Days after Surgery, (E) 1-year Overall Survival (OS) and (F) 1-year Disease Free Survival (DFS).

**Supplementary Figure 4.** Surface Under the Cumulative Ranking Curve of Network Meta-analysis. (A) R0, (B) Pathological Complete Response (pCR), (C) Death within 30 Days after Surgery.

**Supplementary Figure 5.** Subgroup Analysis of R0. (A) analysis based on prospective study, (B) analysis based on retrospective study, (C) analysis based on single-arm study, (D) analysis based on dual-arm study, (E) analysis based on PD-1 drugs, (F) analysis based on PD-l1 drugs, (G) analysis based on ESCC, (H) analysis based on EAC.

**Supplementary Figure 6.** Subgroup Analysis of Pathological Complete Response (pCR). (A) analysis based on prospective study, (B) analysis based on retrospective study, (C) analysis based on single-arm study, (D) analysis based on dual-arm study, (E) analysis based on PD-1 drugs, (F) analysis based on ESCC.

**Supplementary Figure 7.** Subgroup Analysis of Major Pathological Response (mPR). (A) analysis based on prospective study, (B) analysis based on retrospective study, (C) analysis based on single-arm study, (D) analysis based on dual-arm study, (E) analysis based on PD-1 drugs, (F) analysis based on ESCC.

**Supplementary Figure 8.** Subgroup Analysis of Death within 30 Days after Surgery. (A) analysis based on prospective study, (B) analysis based on retrospective study, (C) analysis based on single-arm study, (D) analysis based on dual-arm study, (E) analysis based on PD-1 drugs, (F) analysis based on ESCC.

**Supplementary Figure 9.** Subgroup Analysis of 1-year Overall Survival (OS). (A) analysis based on prospective study, (B) analysis based on retrospective study, (C) analysis based on single-arm study, (D) analysis based on PD-1 drugs, (E) analysis based on ESCC.

**Supplementary Figure 10.** Subgroup Analysis of 1-year Disease Free Survival (DFS). (A) analysis based on prospective study, (B) analysis based on retrospective study, (C) analysis based on single-arm study, (D) analysis based on dual-arm study, (E) analysis based on PD-1 drugs, (F) analysis based on ESCC.

**Supplementary Figure 11.** Sensitivity Analysis by Omitting Each Trial One by One. (A) for the analysis of R0, (B) for the analysis of pathological complete response (pCR), (C) for the analysis of major pathological response (mPR), (D) for the analysis of death within 30 days after surgery, (E) for the analysis of 1-year overall survival (OS) and (F) for the analysis of 1-year disease free survival (DFS).

**Supplementary Figure 12.** Begg’s Test Funnel Plot of the Publication Bias. (A) for R0, (B) for pathological complete response (pCR), (C) for major pathological response (mPR), (D) for death within 30 days after surgery, (E) for 1-year overall survival (OS) and (F) for 1-year disease free survival (DFS).
